# If You Are Old, Videos Look Slow. The Paradoxical Effect of Age-Related Motor Decline on the Kinematic Interpretation of Visual Scenes

**DOI:** 10.3389/fnhum.2021.783090

**Published:** 2022-01-05

**Authors:** Claudio de’Sperati, Marco Granato, Michela Moretti

**Affiliations:** ^1^Laboratory of Action, Perception and Cognition, School of Psychology, Vita-Salute San Raffaele University, Milan, Italy; ^2^Department of Computer Sciences, University of Milan, Milan, Italy

**Keywords:** aging, motion perception, motor slowing, speed bias, videos

## Abstract

Perception and action are tightly coupled. However, there is still little recognition of how individual motor constraints impact perception in everyday life. Here we asked whether and how the motor slowing that accompanies aging influences the sense of visual speed. Ninety-four participants aged between 18 and 90 judged the natural speed of video clips reproducing real human or physical motion (SoS, Sense-of-Speed adjustment task). They also performed a finger tapping task and a visual search task, which estimated their motor speed and visuospatial attention speed, respectively. Remarkably, aged people judged videos to be too slow (speed underestimation), as compared to younger people: the Point of Subjective Equality (PSE), which estimated the speed bias in the SoS task, was +4% in young adults (<40), +12% in old adults (40–70) and +16% in elders. On average, PSE increased with age at a rate of 0.2% per year, with perceptual precision, adjustment rate, and completion time progressively worsening. Crucially, low motor speed, but not low attentional speed, turned out to be the key predictor of video speed underestimation. These findings suggest the existence of a counterintuitive compensatory coupling between action and perception in judging dynamic scenes, an effect that becomes particularly germane during aging.

## Introduction

There is increasing recognition of the reciprocal links between action and perception at both neural and behavioral levels (e.g., Cattaneo and Rizzolatti, 2009; Gallese et al., 2009). However, it is not too clear how individual motor characteristics or constraints impact on perception in everyday life, except perhaps for special populations such as for example sport professionals or people with motor impairments or distinctive movement abilities (Sgouramani and Vatakis, 2014; Bassolino et al., 2015; Voyer and Jansen, 2017; Quarona et al., 2020). A case of a familiar motor constraint in everyday life is the age-dependent motor decline. Perceptual processing in elders might turn out to be affected not only by sensory and cognitive deterioration (Salthouse, 1996; Owsley, 2011; Andersen, 2012), but also by motor deterioration.

Studying aging from the perspective of motion perception seems to be a promising approach (Billino and Pilz, 2019). To this aim, simple stimuli and tasks targeting low-level visual mechanisms have been typically used (e.g., speed and direction discrimination). By contrast, it is much less clear how precisely does aging affect high-level motion processing (e.g., optic flow, heading, collision avoidance, and driving monitoring; Billino and Pilz, 2019). Because high-level vision is more prone than low-level vision to top-down influences, high-level motion perception seems to be a good terrain to investigate possible influences from the motor system and how they change with age. Here we asked whether motor slowing, a typical aspect of age-dependent motor decline (Birren and Fisher, 1995; Salthouse, 2000), influences the sense of visual speed, a capacity lying between perception and cognition.

Humans have a quite poor sense of visual speed. We have recently shown that young adults are very tolerant to video clips reproduced at a speed different from the original speed. For example, a soccer match reproduced at 1.1x does not give rise to an even minimal perception of unnaturalness (de’Sperati and Baud Bovy, 2017). Moreover, previous exposure is capable of altering the judgment of the “natural” speed of a human action such as walking/running (Mather et al., 2017), thus suggesting that the kinematic interpretation is subjected to adaptation. Despite such uncertainty and malleability in speed perception, there can be rather large biases: participants often judge the original video speed to be too slow (speed underestimation, Rossi et al., 2018). Young kids (6–8 years old) show an exaggerated speed underestimation, as compared to older kids (Zuliani et al., 2019).

We posed three questions concerning the sense of speed in elders, namely: (i) whether elders do show a different bias for video speed, as compared to younger people; (ii) whether such speed bias is related to one’s own motor speed; and (iii) whether such motor-rooted bias goes in the direction of perceiving speed contrast (i.e., a tendency to bradykinesia resulting in perceived speediness of the outside world), or in the opposite direction (i.e., a tendency to bradykinesia resulting in perceived slowness of the outside world).

As to the first point, namely, the very presence of a speed bias in elders, as compared to younger people, this study stems from the observation that children show an exaggerated speed underestimation when judging the natural speed of visual events presented in the form of a video clip (Zuliani et al., 2019). Age may be a determinant of dynamic perception not only during development, when sensory systems, motor control and body structure are rapidly growing, but also when these processes begin to decline. For example, general slowing during aging can impact on several processes, especially in the cognitive domain (Salthouse, 1996, 2000). In addition to cognitive aging, which the majority of studies have so far focused on, perceptual aging has recently attracted the attention of cognitive scientists and neuroscientists as a potential marker of aging (Owsley, 2011; Andersen, 2012). As anticipated, motion processing, in particular, “offers an ideal example for perceptual aging that captures fundamental principles of lifespan development and allows insights into functional dynamics”, at the same time challenging the view that a general functional decline underlies healthy aging (Billino and Pilz, 2019). Unraveling the mechanisms that modulate the sense of speed during life time could shed light on how one particular aspect of the sense of reality, namely, kinematic interpretation of motion scenes, evolves with age.

As to the second point, namely, the possibility that the speed bias is correlated to motor speed, there is a large body of evidence that indicates a close interplay between action and perception at both behavioral and neural levels (e.g., Cattaneo and Rizzolatti, 2009; Gallese et al., 2009). The perception of visual kinematics, in particular, has been shown to reflect motor rules (de’Sperati and Stucchi, 1995, 1997, 2000; Viviani et al., 1997; Lacquaniti et al., 2014). Thus, it could be expected that the influence of motor processes on perception extends to speed judgments of observed events. Indeed, it has been reported that one’s own motor speed, as measured in a grasping task, influences visual sensitivity to the speed of observed grasping movements (Macerollo et al., 2015). In this view, elders would be particularly prone to speed biases, as their movements become progressively slower. Such influence could be domain-specific, i.e., the sense of visual speed could depend on motor speed but not necessarily on more general processing speed. For example, the speed at which visual attention can be allocated may have little to do with the kinematic interpretation of visual scenes.

As to the third point, namely, the direction of bias, one hypothesis is that reduced motor speed makes the world to look faster. This hypothesis is rather intuitive, as many events in the world are clearly faster for elders if the reference is one’s own (slower) movement speed—think, e.g., sports scenes or even just normal motor actions of other people. This contrast between one’s own slowness and observed movements would go in the direction of perceiving the world to be too fast. According to this hypothesis—we call it the “*contrast hypothesis*”—tendency to bradykinesia in elders should be associated with visual speed overestimation, as compared to younger people. Alternatively, reduced motor speed could make the world look slower. This hypothesis is somewhat counterintuitive but could result from a tendency to compensate for reduced visual stimulation. Indeed, the optic flow tends to be reduced in elders as compared to younger people, due to their reduced mobility, and therefore the speed of observed events might not be sufficient to restore the normal levels of visual stimulation. Hence, observed events may paradoxically look too slow. According to this hypothesis—we call it the “*compensation hypothesis*”—tendency to bradykinesia in elders should be associated with visual speed underestimation, as compared to younger people. Thus, the direction of speed bias could shed light as to the visuo-motor mechanisms at play when interpreting the kinematics of visual scenes—a mere contrast effect or a compensatory bias.

To address these questions, we asked participants aged between 18 and 90 to judge the natural speed of video clips reproducing real human or physical motion (SoS, Sense-of-Speed task). To estimate motor speed, they also performed a simple finger tapping task, while to estimate attentional speed they performed a visual search task. The relationships between age and the performances in these tasks were studied.

## Methods

### Participants

Due to the pandemic conditions, participants were recruited through chain referral sampling with the help of students of master’s degree in psychology at the Vita-Salute San Raffaele University, who volunteered in the study. Besides serving as participants themselves, students were invited to involve their families, friends, and cohabiting people to take part in the study as participants, targeting especially the elder population. Students were trained at administering the tasks and questionnaires at participants’ homes, and at dealing with the computer task software management, as well as at taking safety measures. Neither the students nor the other participants were aware of the aims of the study, which were presented and discussed only when the entire acquisition phase was over. Nineteen students were involved, who gathered data from 84 people (27 males, 32%) aged between 18 and 90 years old (*M* = 50.58, SD = 23.92), including themselves. Of these, 25 participants belonged to elder population (>70 years-old, *M* = 80.20, SD = 3.31, 28% males), 27 to the old adult population (40–70 years-old, *M* = 55.33, SD = 4.17, 33% males) and 32 to the young adult population (<40 years-old, *M* = 23.44, SD = 2.75, 34% males). Data acquisition was conducted over a 3-month period. Written informed consent was obtained from all participants. The study protocol was approved by the ethical committee of the Università degli Studi di Milano.

### Stimuli and Tasks

The three tasks were administered in a fixed order (first the visual search task, then the tapping task, and lastly the sense of speed task). The visual search task was administered on paper, while the other two tasks were run on participants’ computers. Data were collected individually in a quiet and darkened room at the participant’s home in a single session lasting approximately 40 min, including familiarization.

#### Visual Search Task

Elders tend to be slower in visual search tasks (Mason et al., 1985). To evaluate the speed of selective attention in visual search we used the Spinnler’s matrices task (Abbate et al., 2007). The task is structured as three sets of 11 × 10 matrix of numbers, containing respectively 1, 2 and 3 numbers representing the target(s), with the other numbers representing distractors. Participants have to detect the target numbers while sequentially inspecting each matrix in 45 s, and mark detections (either correct or incorrect) as circles on numbers. Each target appears 10 times in each matrix set and thus participants have to identify 10, 20, and 30 targets in the 1st, 2nd and 3rd matrix set, respectively. To quantify the performance, we used two indexes, one for attentional speed (actually attentional slowness) and the other for attentional accuracy, defined respectively as the total execution time (i.e., the sum of the times taken to complete each matrix set, which could exceed the 135 s total time available for detection) and the overall percentage of hits (i.e., the ratio between the sum of successfully detected targets within 45 s in each matrix set and the total number of targets). The task was administered under the experimenter’s control, which measured the execution time with a stopwatch and, in the end, counted the circled targets (both correct and incorrect).

#### Tapping Task

This is a task that we have implemented to easily and quickly measure motor speed and motor accuracy without requiring *ad-hoc* sensors. Indeed, tapping is often used to study the motor decline in aging (Turgeon et al., 2011), and is a more direct measure than rating motor habits through questionnaires (Zuliani et al., 2019). Participants had to perform alternate finger tapping between two keyboard keys, using the index finger of the dominant hand. Before the beginning of the task, the experimenter identified two keyboard keys aligned at a horizontal distance of about 10 cm (this depended on each participant’s computer, but were mostly the keys “*e”* and “*i”*) and settled the acquisition program accordingly. To facilitate the task, two colored stickers were placed on the two keys. The task consisted of two sessions, each lasting 12 s. In the first session, participants performed finger tapping at a self-paced, relaxed rhythm, while in the second session they had to perform tapping at their maximal speed. To quantify the performance, we used two indexes, one for motor speed (actually motor slowness) and the other for motor accuracy, defined respectively as the median inter-tap interval (i.e., the time elapsed between a key release and the next key pressure) and the complementary percentage of tapping errors (wrong key pressing). This was done separately for each session (speeded tapping and relaxed tapping).

#### SoS Task

The Sense of Speed task was used in previous studies with adults and children and aims at assessing the capacity of participants to adjust the speed of video clips to the speed that they repute to be the original speed (see Rossi et al., 2018; Zuliani et al., 2019 for details). The task is based on the psychophysical adjustment procedure, as exemplified by the instructions to participants: “Now you will view a few short video clips, which will always start at a wrong speed. You will need to adjust their speed with these two keys (showing them to participants) until you reach the speed that you believe is correct. Each time you press one of these two keys, it will either slow the video down a bit (“1” key) or make it a little faster (“9” key). So, whenever you believe the video is going too slow, you should press the “9” key to make it faster, whereas in case the video is going too fast, you should press the “1” key to make it slower. When you think the video speed is correct, press the Enter key and move to the next video. In case you do not press the Enter key, the video will change on its own within 60 s.”

The video clips were displayed centered in the monitor, with the original video aspect ratio (16:9). The stimulus presentation program forced the display area to 13 inches, regardless of the actual monitor. The original speed of the video clips was 30 fps, with a 1,280 × 720 resolution. Each video clip was presented 4 times, alternating trials with an initial reproduction speed lower than the original video speed and trials with an initial reproduction speed higher than the original video speed, randomly selected in the 15–20 and 45–60 fps range, respectively. We used three video clips in looping mode with no audio track, which are briefly described below.

##### Dribbling Video Clip

A 30-s homemade shot of a man dribbling a soccer ball. This video was already used in previous studies with adults and children (Rossi et al., 2018; Zuliani et al., 2019).

##### Water Video Clip

A 30-s homemade shot of an undertow of the sea. This video was already used in previous studies with adults and children (Rossi et al., 2018; Zuliani et al., 2019).

##### Grasping Video Clip

A 12-s homemade shot of a grasping action where the actor starts an arm movement from the body midline, then picks a small object located on a table to the left of the body midline, then releases it to the right of the body midline, and returns to the initial position, from where another grasping cycle begins by picking now the object on the right side and releasing it on the left side, then back again to the initial position. The movement is executed smoothly, with a slight break between grasping cycles. Each grasping cycle lasts about 3 s.

**Figure 1:**
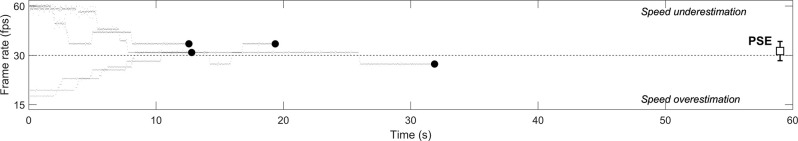
Example of single-trial adjustment traces over time in an individual participant (female, 26 year-old), as assessed through the instantaneous video frame rate (the traces are noisy because it is shown the time stamp of the video frame flipping on the graphics hardware, which is not under full experimental control; however, temporal smoothing in the visual system masks these microscopic frame rate irregularities). Each filled circle marks the video speed at the time of the keypress (actually the median frame rate during the previous 500 ms, to prevent undesired effects of noise), which confirmed the participant’s speed choice and passed to the next trial. Also illustrated is the point of subjective equivalence (PSE, rightward open square, computed as the mean of the final video speed of each trial, which in this case was slightly higher than the original video speed, indicating speed underestimation) together with the standard deviation (error bar, from which the coefficient of variation is computed, see text). The horizontal dashed line indicates the original video clip speed (30 fps).

The primary measure in this task was the Point of Subjective Equality (PSE), which indexed the speed bias, computed as the mean of the final frame rate adjusted by participants for each video clip over the four repetitions (Figure 1). The same results were obtained using the median to compute PSE (data not shown). Values higher than the original video frame rate (30 fps) indicate speed underestimation (the original video speed is reputed to be too low), while values lower than the original video speed indicate speed overestimation (the original video speed is reputed to be too high). We also computed the coefficient of variation (CV), which indexed the precision of speed judgments (actually its opposite, i.e., the uncertainty of speed judgments), computed as the standard deviation of the final frame rate adjusted by participants for each video clip over the four repetitions, divided by the PSE and expressed as a percentage. Additional measures were the adjustment rate (ADJ), i.e., the average number of keystrokes used for adjusting video speed during a trial (but the same results were obtained by measuring the mean video speed change during a trial, data not shown), and the completion time (CT), i.e., the average time participants took to complete a trial. The extended data-set of single-trial adjustment traces is attached as Supplementary Material.

For both the SoS task and the tapping task, the experiment was controlled by compiled Matlab scripts (MathWorks, Nattick, MA, USA) with the Psychtoolbox extension. The scripts were built on participants’ computers (Windows only) through a remote procedure.

### Selection Criteria

In this investigation, we have collected data only from people without a (self-reported) history of disabilities or diseases, who may have had difficulties with the procedure. Nonetheless, prior to the main testing, participants were administered the Mini-Mental State Examination (MMSE) for a quantitative assessment of their general mental state. Only two participants had a score <24 (23 and 22), and only one had a score <20 (17). Because in no case did the MMSE score indicate severe dementia, no participants were excluded on this basis.

Due to the “distributed” nature of the study, the main concern was to exclude dirty data resulting from insufficient hardware performance in the SoS task, which could have been too computationally demanding for running flawlessly on ordinary consumer computers. Indeed, the graphic hardware could not always follow the desired inter-frame interval in real-time as adjusted by the participant, with unpredictable effects on the sense of speed and consequent adjustments (for example, the participant could have experienced a whimsical video speed control with a poor sense of agency).

In order to exclude trials with potentially inappropriate adjustments, we used the following inclusion criteria, elaborated on intuitive ground: completion time of at least 3 s (considered to be a minimal duration to appreciate video speed), at least one adjustment (to avoid erroneous passage to the next trial), final video frame rate between 20 and 55 Hz (to exclude implausible judgments), and at least two repetitions for each video clip (to allow the computation of the coefficient of variation). As a result of this automatic procedure, 8% of participants (7/84) were excluded from the analyses.

Further three participants were excluded on the basis of visual inspection of instantaneous video frame rates in individual trials. This was an entirely subjective evaluation aimed at excluding noisy trials that may have survived the automatic selection (e.g., with highly variable frame rates or in which the final adjustment was not convincingly related to the adjustment process). The effect of this additional arbitrary selection was controlled *a-posteriori* in a trial-wise analysis (see below). Thus, as a result of cumulative trial exclusion (i.e., combining automatic criteria and visual inspection), 12% of participants (10/84) were excluded from SoS data analyses.

By contrast, only four participants did not perform successfully in the tapping task (two participants were wrong about keypresses, and data from the other two participants were missing), and three in the visual search task (one participant went distracted during the task, and data from other two participants were missing).

In the multiple regression analysis (see below), 17 participants were excluded because of the need of having matched data (as anticipated, 10 because of problems with the SoS task, four with the tapping task, and three with the visual search task). This corresponds to a 20% rejection rate. Of the remaining 67 participants, 20 belonged to elder population (*M* = 79.30, SD = 5.09), 23 to the old adult population (*M* = 55.70, SD = 4.31) and 24 to the young adult population (*M* = 24.12, SD = 2.54).

### Data Analyses

For analyzing data from individual tasks, we used one-way ANOVA (Matlab *anova1* function) and both linear and non-linear regression (Matlab *fit* function). When all predictors from the three tasks were considered, we used robust multiple regression (Matlab *fitlm* function). The goodness of fit was controlled by means of diagnostic plots (leverage, Cook’s distance, and covariance ratio), as well as the Durbin-Watson test for autocorrelation in the residuals, and the Variance Inflation Factor (VIF) for multi-collinearity. If not otherwise specified, when the 95% confidence intervals of the regression coefficients did not cross zero or did not overlap, it is implied that *p* < 0.05. For the multiple regression analysis, the point of subjective equivalence, coefficient of variation, adjustments, and completion time were averaged subject-wise in order to match the individual indexes (motor and attentional speed and accuracy, plus age and gender). This resulted in 55 degrees of freedom.

The multiple regression analysis was integrated and extended by a trial-wise analysis based on Linear Mixed Models (LMM), which was performed by means of the MATLAB *fitlme* function. The dependent variable was the final adjusted video speed in each trial. To reduce the complexity of the model, fixed and random factors were kept to a minimum (Zuliani et al., 2019), thus, we excluded the fixed interaction terms and used only the intercepts of the random factors. The model included participant, video clip, and experimenter (the student who administered the tasks) as random factors, and tested also the fixed effects of MMSE score, trial selection (whether or not a trial was initially excluded based on visual inspection: this way, we could rely on a more extended—though noisier—dataset, but controlling for this factor) and initial video clip (because the final video speed for ascending and descending traces did not fully converge, see below). In the Wilkinson notation, the model was [final video speed ~1 + age + gender + MMSE score + adjustment + coefficient of variation + completion time + motor speed + motor accuracy + attentional speed + attentional accuracy + initial video speed + selection + (1 | participant) + (1 | clip) + (1 | experimenter)]. This analysis relied on trial-wise measures (i.e., the final video speed plus the other single-trial measures: initial speed, adjustments, and completion time) coming from 686 trials in 80 participants, as well as clip-wise (clip type and coefficient of variation) and subject-wise (motor and attentional speed and accuracy, plus age, gender, and MMSE score) indexes, for a total of 521 degrees of freedom.

## Results

### Visual Search Task

On an average, participants completed the Spinnler’s matrices task in less than the 135 s maximum allowed time (*M* = 106.793 s, SD = 31.415) and with fairly good accuracy (*M* = 89.5%, SD = 10.6). Both attentional speed and attentional accuracy depended on participants’ age. Namely, older participants tended to be significantly slower (slope = 0.614 s/year, confidence interval = 0.354–0.875) and less accurate (slope = −0.2%/year, confidence interval = −0.3 to −0.1) than younger participants.

### Tapping Task

Participants were quite compliant in performing the finger tapping task, attaining a mean tapping speed of ~3 Hz (actually a tapping rhythm) in the speeded tapping session (inter-tap interval: *M* = 0.327 s, SD = 0.125), and ~1.5 Hz in the relaxed tapping session (inter-tap interval: *M* = 0.613 s, SD = 0.284). Keystroke errors were rare in either session (accuracy: *M* = 97.9%, SD = 4.5, and *M* = 97.6%, SD = 10.1, respectively in the speeded and relaxed session).

However, older participants were slower and somewhat less accurate than younger participants. Indeed, age significantly affected the tapping speed (speeded tapping, slope = 0.004 s/year, confidence interval = 0.003–0.005, *p* < 0.05; relaxed tapping, slope = 0.006 s/year, confidence interval = 0.004–0.008, *p* < 0.05) as well as tapping accuracy, though only in relaxed tapping (slope = −0.1%/year, confidence interval = −0.2 to −0.01, *p* < 0.05).

In addition, we computed the reduction of the tapping speed over the 12-s trial duration. On average, inter-tap interval became longer over time (+11.8%), indicating a tendency to slow down the tapping speed, and this tendency became more marked with age (slope = 0.47%/year, confidence interval = 0.03–0.90, *p* < 0.05).

### SoS Task

Participants took on an average about 20 s to adjust video speed (*M* = 19.110 s, SD = 8.550), hitting on average eight keystrokes (*M* = 7.632, SD = 3.047). However, in adjusting video speed older participants tended to be slower (slope of completion time over age, 0.111 s/year, confidence interval = 0.061–0.160, *p* < 0.05) and less active (slope of adjustments over age, −0.041 keystrokes/year, confidence interval = −0.058 to −0.023, *p* < 0.05). Perceptual precision (indexed by the coefficient of variation) progressively worsened with age (slope = 0.1%/year, confidence interval = 0.045–0.154, *p* < 0.05).

**Figure 2:**
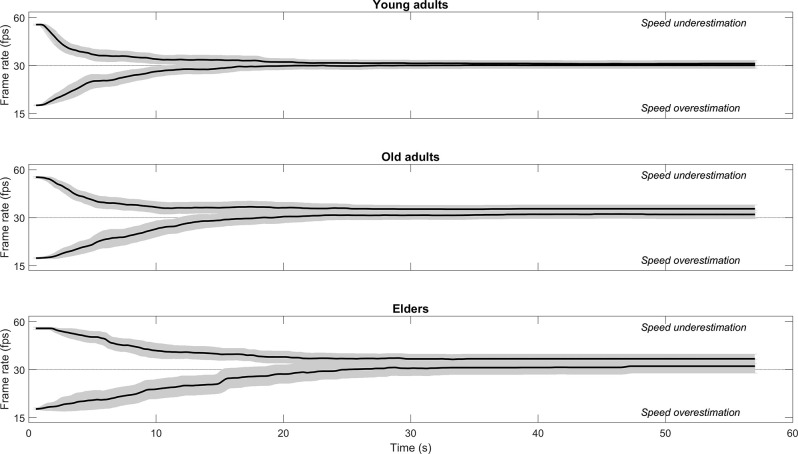
Average adjustment traces in the three age groups. The final video speed tends to be higher than the original video speed, and this tendency was more marked for old adults and elders. The descending and ascending adjustment traces (when the initial video speed was higher or lower than the original video speed, respectively) did not fully converge, remaining always somewhat separated. In general, older participants tended to be slower in adjusting video speed. A 15-points smoothing was applied. The gray region represents the instantaneous 95% confidence band.

**Figure 3:**
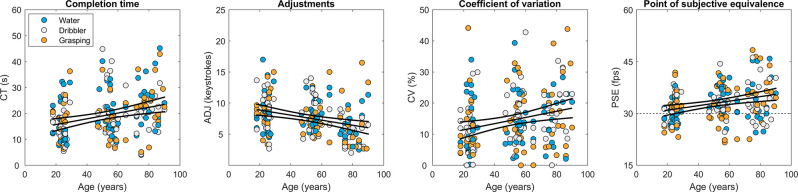
The four indexes of the SoS task (CT, completion time; ADJ, adjustments; CV, coefficient of variation; PSE, Point of subjective equivalence) as a function of age. Each data-point represents the mean value computed over repetitions for each subject and video clip. Colors represent video clips. Also shown are the fitting curves obtained through linear regression (continuous lines), together with the 95% confidence intervals (simultaneous functional bounds). The horizontal dashed line in the PSE plot indicates the original video clip speed.

These aspects of SoS performance can be appreciated in Figure 2, which illustrates the average adjustment behavior in the three age groups, as well as in the scatterplots of Figure 3, where the SoS indexes are plotted in relation to video clip and participant’s age.

However, the most important SoS index is the Point of Subjective Equality (PSE), which estimated the speed bias, and which can be appreciated likewise in Figure 2 (as the average distance of the final video speed from the original video speed) and in Figure 3. PSE was on average higher by about 10% than the original 30 fps video frame speed, indicating an overall tendency to speed underestimation (*M* = 32.945 fps, SD = 5.220, *t*_(201)_ = 8.018, *p* < 0.001). Importantly, PSE increased significantly with age. Indeed, in elders (>70 years old) the mean PSE was 34.799 fps (SD = 5.589), i.e., 16% higher than the original video speed. In old adults (40–70 years old) the mean PSE was 33.629 fps (SD = 3.878), i.e., 12% higher than the original video speed, while in young adults (<40 years old) it was 31.098 fps (SD = 3.509), i.e., 4% higher than the original video speed. A one-way ANOVA revealed a main effect of age group on PSE [*F*_(2, 71)_ = 4.649, *p* = 0.013], with multiple comparisons (Tukey-Kramer test) indicating a significant PSE difference between young adults and elders (*p* = 0.012) but not between young adults and old adults (*p* = 0.104) or between old adults and elders (*p* = 0.625).

The increase of PSE with age was confirmed by the relation between individual participant’s age and PSE (slope = 0.065 fps/year, confidence interval = 0.035–0.096, *p* < 0.05). Taking as reference the original video speed, this corresponds to an average increase of 0.2%/year (0.065/30 * 100). Because of the lack of data-points in the intermediate age range (~30–45 years), we also computed the PSE slope separately in the group of young adults and in the group of old adults plus elders (these two latter groups had an almost continuous age distribution). In both cases, the slope was positive, but was steeper in the young adult group. This may suggest a non-linear change of the sense of speed with age, with PSE tending to reduce its growth rate over time. Therefore, we tested a power law model, which could reasonably accommodate a tendency to a somewhat steepest initial rise. The power law model produced a non-linear curve fit which was very close to the linear curve fit, with almost fully overlapping confidence intervals (Supplementary Figure S1, Supplementary Material). Thus, we opted to describe the PSE growth in the simplest way, i.e., by providing a linear slope, while at the same time recalling the possibility that the increase of PSE could somewhat reduce over time.

No differences in the PSE slopes were found by comparing data of the three video clips, as their confidence intervals were widely overlapping (data not shown).

### Combined Analyses

**Figure 4:**
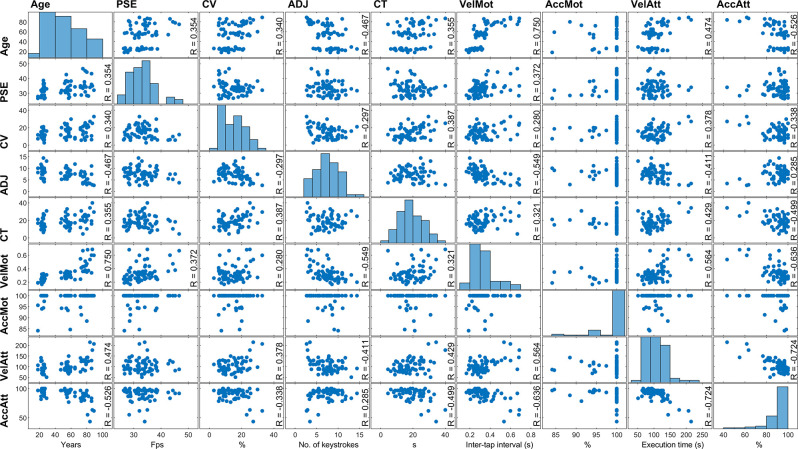
Correlation matrix of the continuous variables considered in this study. Each data-point represents an individual participant. The plots along the diagonal report the frequency distributions. Note that information is repeated in the upper-right and lower-left halves of the figure, but with plots in each pair having inverted X-Y axes, which affords a more comprehensive data visualization. The zero-order correlation coefficient (R) is reported only when *p* < 0.05. PSE, Point of Subjective equivalence; CV, coefficient of variation; ADJ, adjustments; CT, completion time; VelMot, motor speed (speeded tapping); AccMot, motor accuracy (speeded tapping); VelAtt, attentional speed; AccAtt, attentional accuracy. Note that the values for motor and attentional speed represent in fact motor and attentional slowness.

To gauge a more comprehensive picture of the relation of video speed bias with age, motor performance, and attention performance, we plotted the correlation matrix, which depicts the correlation between the pairs of variables (Figure 4). It can be seen that age was significantly correlated with all variables (except motor accuracy), while PSE was significantly correlated with age and motor speed. In order to identify the specific predictors of video speed bias, we ran a multiple regression analysis, entering PSE as a dependent variable and using as predictors the other SoS indexes (coefficient of variation, adjustments, completion time), motor speed and motor accuracy (computed from the speeded tapping task), attentional speed and attentional accuracy (computed from the visual search task) as well as participant’s age and gender. The model accounted for 37% of the total variance, and the Durbin-Watson test was not significant (*p* = 0.186). The variance inflation factor was always very low (VIF ≪ 5).

**Figure 5:**
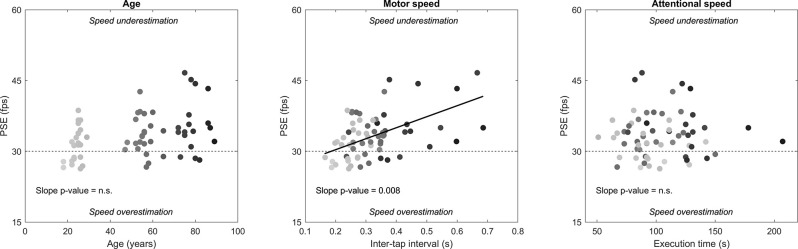
Effects of participants’ age, motor speed (tapping task), and attentional speed (visual search task) on PSE (SoS task), as assessed through multiple regression. Each data-point represents an individual participant, where the greytone represents the participant’s age (darker = older). PSE tended to increase with age, but the effect was carried by motor speed, which was the only significant predictor of PSE (continuous line). Note that the values for motor and attentional speed represent in fact motor and attentional slowness. The horizontal dashed lines indicate the original video clip speed.

When all these variables were included in the model, motor speed emerged as the only significant PSE predictor (Table 1, Figure 5), accounting for almost 8% of the variance (partial correlation coefficient for motor speed = 0.279). Namely, the slower the participants’ in the tapping task, the higher their PSE in the SoS task [slope = 23.312 fps/s, confidence interval = 6.396–40.228, *p* = 0.008]. We recall that a higher PSE goes in the direction of speed underestimation. By contrast, no effect of attentional speed on PSE emerged. The same results were obtained with a lighter model in which the three SoS indexes (coefficient of variation, adjustments, completion time) were not included in order to restrict the predictors to the variables external to the SoS task (data not shown). Therefore, it appears that videos look increasingly slower in older people because of their decreasing motor speed. Indeed, age and motor speed were highly correlated, as shown by the correlation matrix.

**Table 1:** Results of the multiple regression analysis (dependent variable = PSE), together with VIF and accounted variance (squares of the partial correlation coefficients * 100).

Predictor	Slope	Lower bound	Upper bound	*P*-value	VIF	Accounted variance
Age	0.054	−0.019	0.127	0.147	2.489	6.882
Gender	−0.338	−2.994	2.319	0.800	1.355	0.180
CV	−0.085	−0.268	0.097	0.353	1.395	2.610
ADJ	0.196	−0.374	0.767	0.494	1.916	0.212
CT	−0.134	−0.310	0.043	0.135	1.555	2.875
VelMot	23.312	6.395	40.228	0.008	3.427	7.784
AccMot	17.835	−14.627	50.297	0.276	1.164	3.396
VelAtt	−0.026	−0.080	0.028	0.345	2.442	0.144
AccAtt	10.682	−9.705	31.069	0.298	2.946	4.691

The lower and upper bounds indicate the 95% confidence intervals of the slopes. CV, coefficient of variation; ADJ, adjustments; CT, completion time; VelMot, motor speed; AccMot, motor accuracy; VelAtt, attentional speed; AccAtt, attentional accuracy; VIF, variance inflation factor.

Despite the low variance inflation factor, we repeated the analysis by taking collinearity into account (partial least squares approach), which confirmed that only motor slowness was a significant predictor. This held true by using either a commonly-used threshold (variable importance in projection, VIP = 1.00) or a more stringent one (VIP = 1.44, corresponding to the maximum value obtained by running several simulations, see Chong and Jun, 2005). In both cases, only motor slowness and age were retained as predictors in the model, but only the former was statistically significant (*p* = 0.043 vs. *p* = 0.595).

The multiple regression analysis was conducted by averaging data from the three video clips. No significant differences in the slopes of PSE vs. motor speed were found by running the same analysis separately for each video clip, as their confidence intervals were widely overlapping (Water video clip: slope = 24.049 fps/s, confidence interval = 4.970–43.130; Dribbler video clip: slope = 10.549 fps/s, confidence interval = −6.163–27.260; Grasping video clip: slope = 28.560 fps/s, confidence interval = 2.143–54.976).

Interestingly, when the relaxed tapping speed (i.e., measured in the relaxed tapping task) was used instead of the maximal tapping speed (i.e., measured in the speeded tapping task), the significant effect of motor speed on PSE disappeared (slope = 2.158 fps/s, confidence interval = −3.812–8.128), and age returned to be a significant (unspecific) predictor of PSE (slope = 0.116 fps/year, confidence interval = 0.052–0.180).

The same applies when the reduction of maximal tapping speed over time (measured in the speeded tapping task) was used as PSE predictor instead of the maximal tapping speed: the reduction of maximal tapping speed was not a significant predictor of PSE (slope = −1.124 fps/s, confidence interval = −3.731–1.483), and age returned to be a significant (unspecific) predictor of PSE (slope = 0.121 fps/year, confidence interval = 0.054–0.188).

The multiple regression analysis was integrated and extended by a trial-wise LMM analysis. We found the same pattern of results, namely, that maximal motor speed but not relaxed motor speed was a significant predictor of the final video speed judgment, although in the latter case there was no evidence of a trade-off between relaxed motor speed and age. In addition, the initial video speed was a highly significant predictor in both analyses, which is in keeping with the average adjustment traces of Figure 2, where the convergence between the descending trace and the ascending trace was not fully achieved. Fitting produced homogeneous diagnostic plots of residuals and accounted for 55% of the total variance. The results of this analysis are reported in Supplementary Tables S1 and S2 (Supplementary Material).

## Discussion

### Considerations on Data Reliability

Due to the “distributed” nature of the study, the first concern was overall data reliability. We identified three main issues, namely, differences in general set conditions, data loss in the SoS task, and sampling bias. As to the first issue, the luminance and contrast of different computer screens, as well as the characteristics of the testing room, could not be controlled. However, firstly, our students were trained to try to make luminance conditions as similar as possible, and, secondly, image contrast does not affect the sense of video speed (de’Sperati and Moretti, in preparation). Another potentially problematic aspect is screen size, but, as reported in the Methods section, the stimulus presentation program for the SoS task included a function to display the video clips at the same size (13 inches) regardless of the actual screen size. A further potential source of variability is that keyboards were not the same, thus the tapping distance might not be the same in different participants. However, as reported in the Methods section, we adopted a measure to minimize this source of motor variability, ensuring a reasonably uniform inter-key distance. Therefore, we are confident that these aspects, despite potentially adding variability, were not detrimental to the conclusions of the study.

As to the second issue, as much as 12% of participants were excluded from the SoS analyses when strict selection criteria were applied. Although this selection should have ensured that only clean data were entered, yet it might have possibly biased the results. Thus, we repeated the multiple regression analyses using looser exclusion criteria, namely, without applying the subjective criteria based on visual inspection of the adjustment traces. This resulted in only 8% of rejected subjects and yielded the same pattern of results (data not shown). We did not further weaken the rejection criteria because of the increasing risk of including artifacts. However, it is encouraging that we found the same pattern of results by adopting looser selection criteria, as also confirmed by the additional trial-wise LMM analysis on an extended data-set, which did not find evidence that the arbitrary visual selection introduced a bias in the results.

As to the third issue, we note that, while clearly somewhat limiting the generalizability of results, sampling bias (in the present case consisting of recruiting mainly university students’ friends and relatives) does not undermine internal validity.

The choice of the fixed order of task presentation that we have adopted in this study deserves a further, specific comment, as it might have introduced a confound in the results. A fixed order could be associated with increasing tiredness and/or attention decline over time, especially in elders. Because participants were free to take a pause at will between the tasks, we did not consider this aspect to be of particular concern. Nonetheless: (i) even by considering that tiredness and/or attention decline could have made the adjustment task more demanding for older participants (i.e., fewer adjustments and/or longer completion time and/or larger coefficient of variation), we note that none of these indexes were statistically significant PSE predictors; and (ii) as to the theoretical possibility that the prior execution of the attention task could have somehow limited the maximal tapping speed (thus opening to the hypothesis that the PSE decrease in older participant was associated to their higher tiredness impacting on motor speed and not to their slower motor speed *per se*), we note that the slowing of the maximal tapping speed over time was not a significant PSE predictor. Therefore, although we cannot fully rule out some minor carry-over effects due to the fixed order of task presentation, the fact that none of the potential indicators of tiredness and/or attention decline in the SOS task or in the tapping task were associated with PSE suggests that it is unlikely that this aspect of the experimental design created a confound. Conversely, it contributed to keeping similar experimental conditions for all participants.

In sum, although a more controlled study under stringent laboratory conditions would allow to further reduce experimental variability, the above considerations, together with the analyses at multiple levels (ANOVA over age groups, subject-and-clip-wise simple regressions, subject-wise multiple regressions, and trial-wise mixed models), reassure that the conclusions of this study rest on sufficiently solid ground.

### The Sense of Speed in Elders

By considering the data of the SoS task in isolation, i.e., excluding data derived from the other two tasks, a first finding was that the PSE rose significantly in older participants, that is, speed underestimation increased with age. Also, the SoS task appears to be more difficult for older people, as perceptual precision, number of adjustments, and completion time worsened significantly with age. Such general worsening, however, was not responsible for the PSE increase with age, as none of the three indexes (coefficient of variation, adjustments, completion time) was significantly correlated with PSE when considering the SoS data in isolation, or significantly predicted PSE when evaluated through the multiple regression or LMM analyses. This excludes that the PSE increase was merely the result of changes in response variability (coefficient of variation) or adjustment behavior (adjustments and completion time).

Likewise, the results of both the multiple regression and LMM analyses showed that PSE was not predicted by either speed or accuracy of attention allocation, as assessed through the visual search task. This suggests that the capacity of quickly and accurately shifting attention across visual stimuli was not responsible for the PSE increase with age, despite the significant correlation of these two variables with age and the fact that several attention functions decline in elders (Erel and Levy, 2016). The lack of effect of attentional speed on PSE may be taken as a sign that the sense of speed has little to do with processing speed in general, which is known to decrease in elders (Birren and Fisher, 1995; Salthouse, 1996), as also suggested by the lack of effect of MMSE score. This would be in keeping with the notion that “Age-related declines in vision […] occur at multiple levels of the visual system including optics, sensory processing, and perceptual processing, and are not likely due to a systemic change in brain function (e.g., generalized slowing; common cause hypothesis) as a result of normal aging.” (Andersen, 2012).

The only significant predictor of PSE (besides the initial video speed) turned out to be the individual motor speed, as assessed through the tapping task. Interestingly, this seems to be a quite specific effect: the fact that this held true when the tapping maximal speed, but not the relaxed tapping speed, was used as a predictor, points to a relation of speed bias with motor constraints rather than just current motor execution: what counts seems to be the very capability of performing fast movements, not the extemporary tendency to move at a given faster or slower pace. This consideration stems from the difference between the two tapping tasks: the tapping speed in the relaxed tapping task is a matter of choice (participants could speed-up tapping if they wished), whereas the tapping speed that participants could reach in the speeded tapping task is dictated by a motor constraint (it is the maximal tapping speed, participants could not further speed-up tapping in this task). Note that, although tapping does not in itself provide a measure of walking speed or general mobility, it is often used as a proxy of the decline of motor abilities during aging (Turgeon et al., 2011).

Remarkably, the fact that age ceased to be a PSE predictor when tested concurrently with the other predictors indicates that age-related changes other than maximal motor speed (and initial video speed) are not particularly relevant to the sense of speed. These include the specific factors that we have explicitly tested including them as predictors but also other potential age-related factors that we have not tested and which, if relevant, would have contributed to increasing the relevance of the unspecific “age” predictor. Among them, we should mention familiarity with technology/equipment/experimental setup, which could be poorer in older participants. However, age-related non-tested factors, including familiarity, could not have a relevance comparable to that of (maximal) motor speed, for in that case they should have manifested in the form of significant age predictor, which was not the case in this study. Indeed, when maximal tapping speed was removed from the multiple regression model and replaced with the relaxed tapping speed, its effect was taken over by the “age” predictor, which in fact returned to be statistically significant (admittedly, this was not the case with the LMM model, but in that case there was a strong predictor, i.e., initial video speed, that may have masked the effect of the other predictors; despite this, age had a rather low p-value).

Finding specific motor signatures in perceptual processing points to the so-called motor theories of perception, which claim that certain classes of perceptual facts are based upon an internal model of motor acts (Scherer, 1984; Liberman and Mattingly, 1989; Cattaneo and Rizzolatti, 2009; Gallese et al., 2009). A particular aspect of visual perception relevant to the present findings, i.e., perceived kinematics, has indeed been shown to be shaped by motor constraints (e.g., Viviani and Stucchi, 1992; de’Sperati and Stucchi, 1995, 1997, 2000; Viviani et al., 1997; Casile and Giese, 2006), which may be the basis of the observed influence of individual motor styles on action perception (e.g., Wilson and Knoblich, 2005; Koul et al., 2016; Hilt et al., 2020; Vidal and Lacquaniti, 2021). In principle, the links between action and perception can be due to an interplay between visual and motor neural circuits (*à la* Helmholtz) or pass through visual reafference, i.e., optic flow (*à la* Gibson). Current predominant positions based on mirror mechanisms favor a direct neural link (Rizzolatti and Craighero, 2004; Cattaneo and Rizzolatti, 2009; Gallese et al., 2009). As we will see, however, the present findings may indicate a role for visual reafference.

Thus, regarding our first and second questions, i.e., whether elders show a different bias for video speed, as compared to younger people, and whether such speed bias is related to one’s own motor speed, we can answer that elders do show increased speed underestimation and that this underestimation depends specifically on their reduced mobility. For this reason, it may be worth upgrading the previously mentioned statement about age-related decline in vision (Andersen, 2012), which would then “occur at multiple levels of the visual system including optics, sensory processing, and *perceptual-motor* processing”.

It is possible that sports professionals or simply people practicing sports are subjected to the same perceptual bias in their sense of speed, regardless of their age. Indeed, in general the capacity of elaborating visual stimuli, including visual kinematics, is enhanced in sport professionals, and part of their visual characteristics might originate in their special motor repertoire (see e.g., Bläsing et al., 2012; Vidal and Lacquaniti, 2021). However, lacking specific evidence concerning the sense of speed, this remains an open question.

### A Compensatory Bias?

We found that motor slowness was associated with a PSE increase, that is, increased speed underestimation, and not *vice-versa* (i.e., decreased speed underestimation). What could be the reason for such a rather counterintuitive phenomenon? As anticipated in the Introduction, this finding may suggest that the perceived norm for dynamic visual events is biased towards faster speeds because of a tendency to restore normal (i.e., higher) visual speed levels. Being functional to compensating optic flow, this explanation would then not configure as a Helmholtzian mechanism but rather as a Gibsonian mechanism.

The fact that the pattern of results was the same for the three video clips seems to go in this direction: should motor neural circuits impress their signature on the inner working of motion perception, one would expect that this effect is specific to certain motor-movement coupled patterns, for example manifesting as a stronger effect on human actions (e.g., our clips “Dribbling” and especially “Grasping”, with the latter representing an action—repetitive grasping—very similar in structure and rhythm to the tapping task, see Macerollo et al., 2015), as compared to other types of movements (e.g., clip “Water”). On the contrary, because the optic flow is a general visual stimulation, speed underestimation should apply indiscriminately to all visual stimuli, which is what our results indicate.

However, although optic flow compensation could explain increased speed underestimation, it should be recalled that we did not systematically manipulate content or motion/optic flow characteristics of video clips, as we used them only as an arbitrary stimulus sample. Hence, whether indeed the compensatory bias arises from the reduced optic flow is a point that deserves further investigation.

### Fast Is Better?

It may appear that, contrary to diffuse wisdom that slow is better, elders might prefer to watch video clips at a somewhat faster speed than their original speed. Indeed, the results of this study showed that, when requested to adjust videos to their original speed, elders set a speed higher than the original speed (+16% on average), and also higher than young adults (in whom speed underestimation was on average +4%, in line with our previous report, Rossi et al., 2018). Does that mean that the optimal video speed for elders is higher than the original speed? Would elders speed up videos when, say, watching TV if they had a hand-held speed controller? The answer is not straightforward. Clearly, one thing is to adjust the reputed “natural” speed of a video clip, like our participants were asked to do in this study, and another thing is to set the speed in order to optimally follow, say, a film which requires an understanding of the story, plot, dialogues, etc., i.e., a rather more complex condition that we did not target in this study. On intuitive grounds, we speculate that in the latter case a somewhat slower speed—not a higher speed—may be better suited for elders to keep up with event understanding. However, we also submit that for videos reproducing poorly demanding scenes, for example, simple sports scenes with little or no voice-over or also naturalistic documentaries, matching the reproduction speed to the subjective (higher) natural speed may be recommended when no particular scene understanding is required. This does not contradict the possibility of preferring a slower speed when it is important to grasp complex events unfolding, or simply to enjoy relaxed rhythms.

## Conclusions

This study showed that elders tend to judge videos to be too slow, as compared to young adults. It is difficult to tell at which age does this phenomenon precisely arises, as we did not test the intermediate age range (~30–45 years), although old adults would seem to be already biased. Importantly, however, our results suggest that such speed underestimation is not simply the consequence of aging *per se*, but depends on the progressive motor slowing while getting older. The seemingly paradoxical finding that observed scenes look too slow rather than too fast (considering one’s own slow motor speed as a reference) could be explained by positing a visuo-motor mechanism based on optic flow compensation.

## Data Availability Statement

Data and Matlab code are available at: https://data.mendeley.com/datasets/g5dydytnry/1.

## Ethics Statement

The studies involving human participants were reviewed and approved by the Ethical Committee, Università degli Studi di Milano. The patients/participants provided their written informed consent to participate in this study.

## Author Contributions

CS planned the study, performed the analyses, and wrote the article. MG organized and trained the students to perform data acquisition, wrote the software, and performed the analyses. MM organized and trained the students to perform data acquisition. All authors contributed to the article and approved the submitted version.

## Conflict of Interest

The authors declare that the research was conducted in the absence of any commercial or financial relationships that could be construed as a potential conflict of interest.

## Publisher’s Note

All claims expressed in this article are solely those of the authors and do not necessarily represent those of their affiliated organizations, or those of the publisher, the editors and the reviewers. Any product that may be evaluated in this article, or claim that may be made by its manufacturer, is not guaranteed or endorsed by the publisher.
