# Supplementary material for: If You Are Old, Videos Look Slow. The Paradoxical Effect of Age-Related Motor Decline on the Kinematic Interpretation of Visual Scenes
Source: Front Hum Neurosci. 2022 Jan 5;15:783090. doi: 10.3389/fnhum.2021.783090 (PMC8766849; doi:10.3389/fnhum.2021.783090)
Supplement: Supplementary file 1 [file Presentation_1.PPT]

## Slide 1
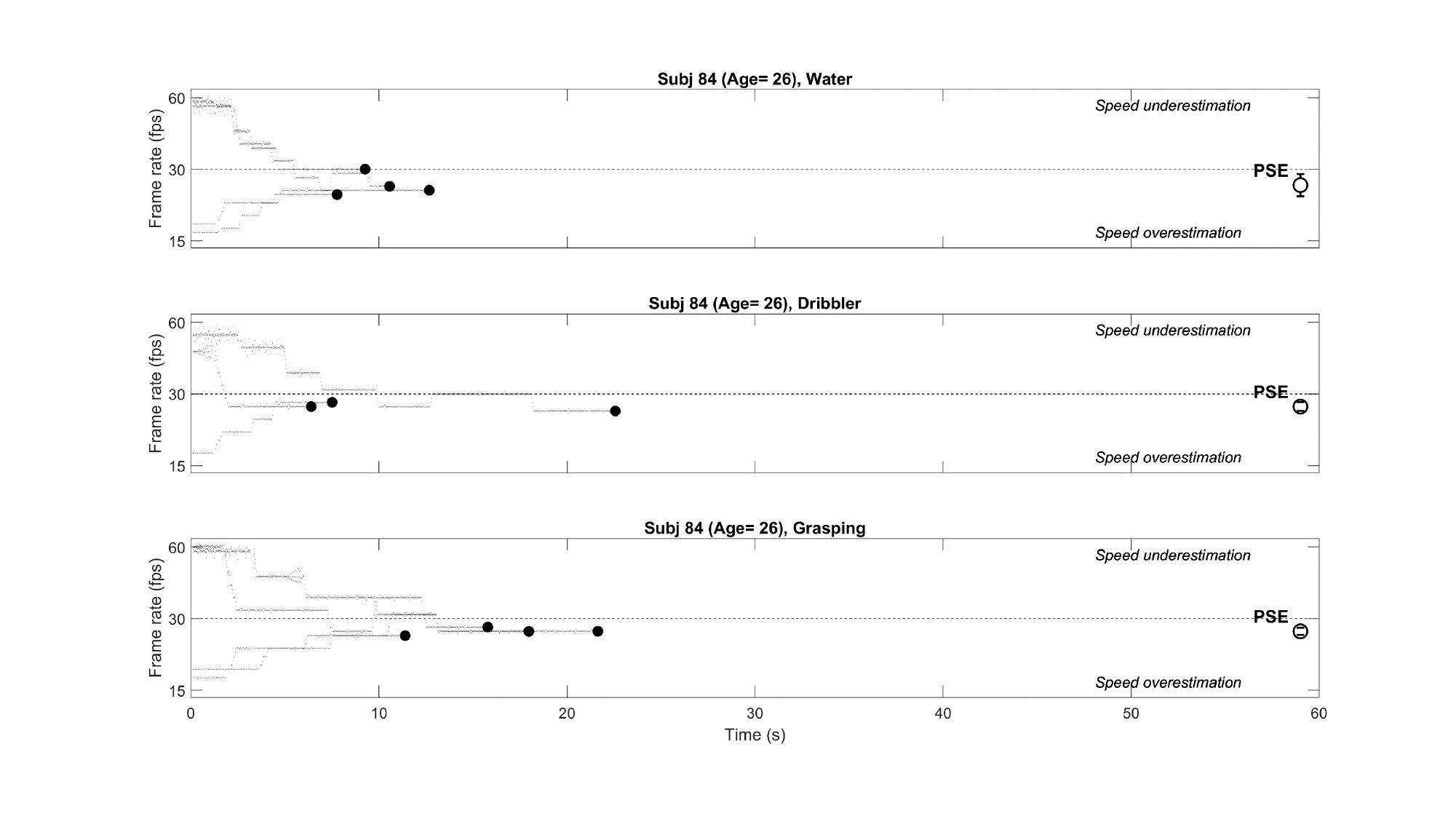

#

## Slide 2
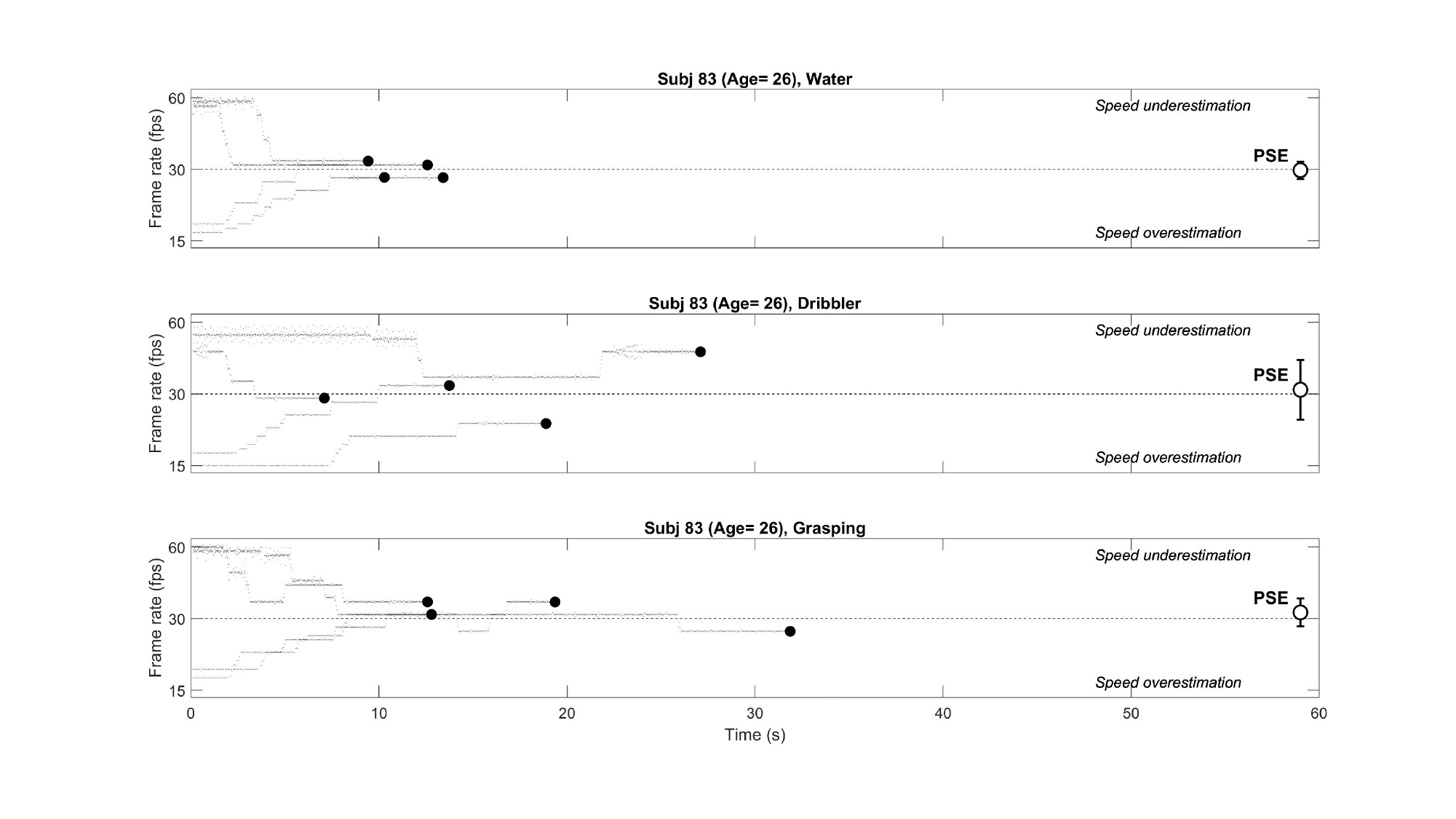

#

## Slide 3
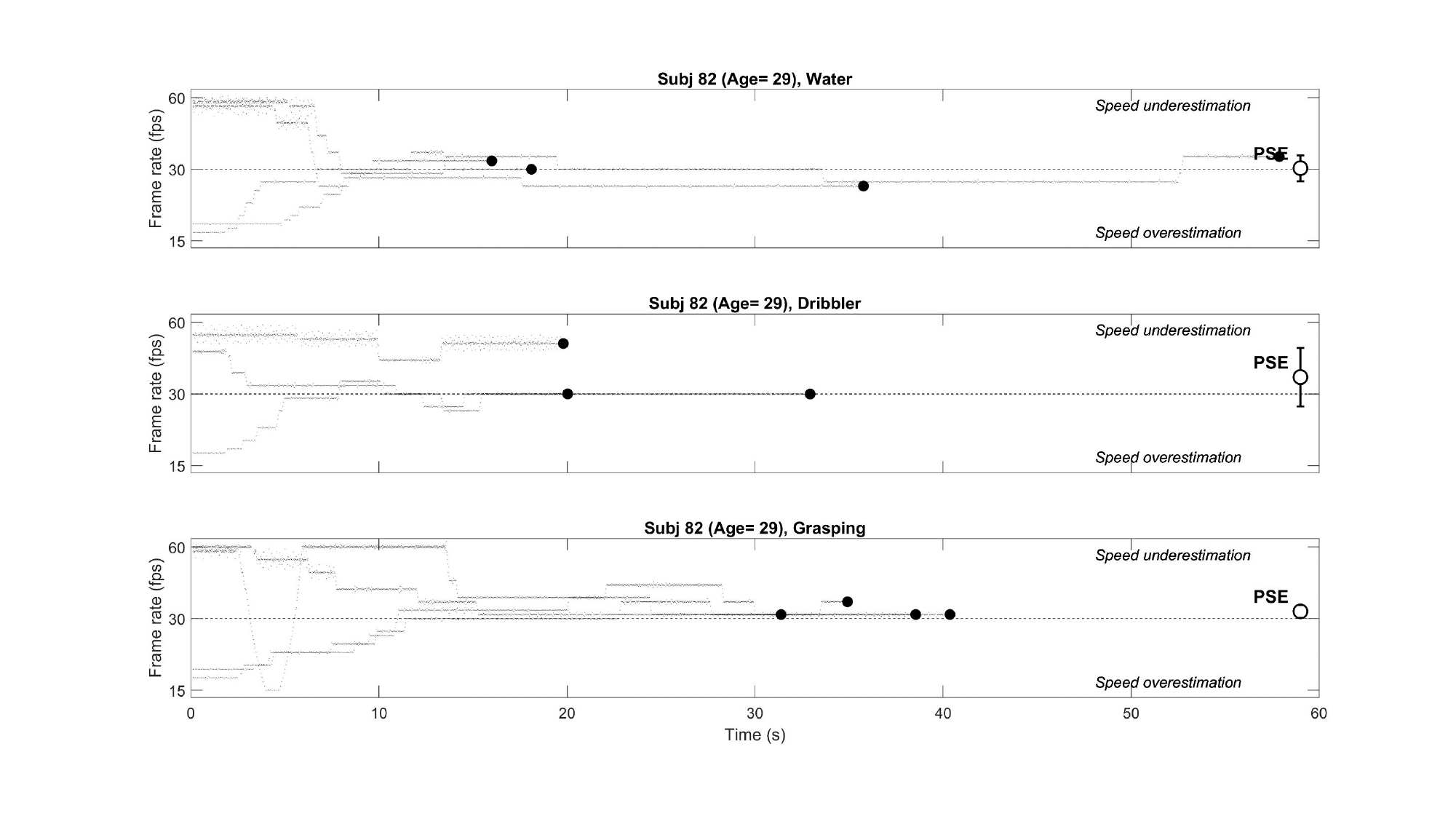

#

## Slide 4
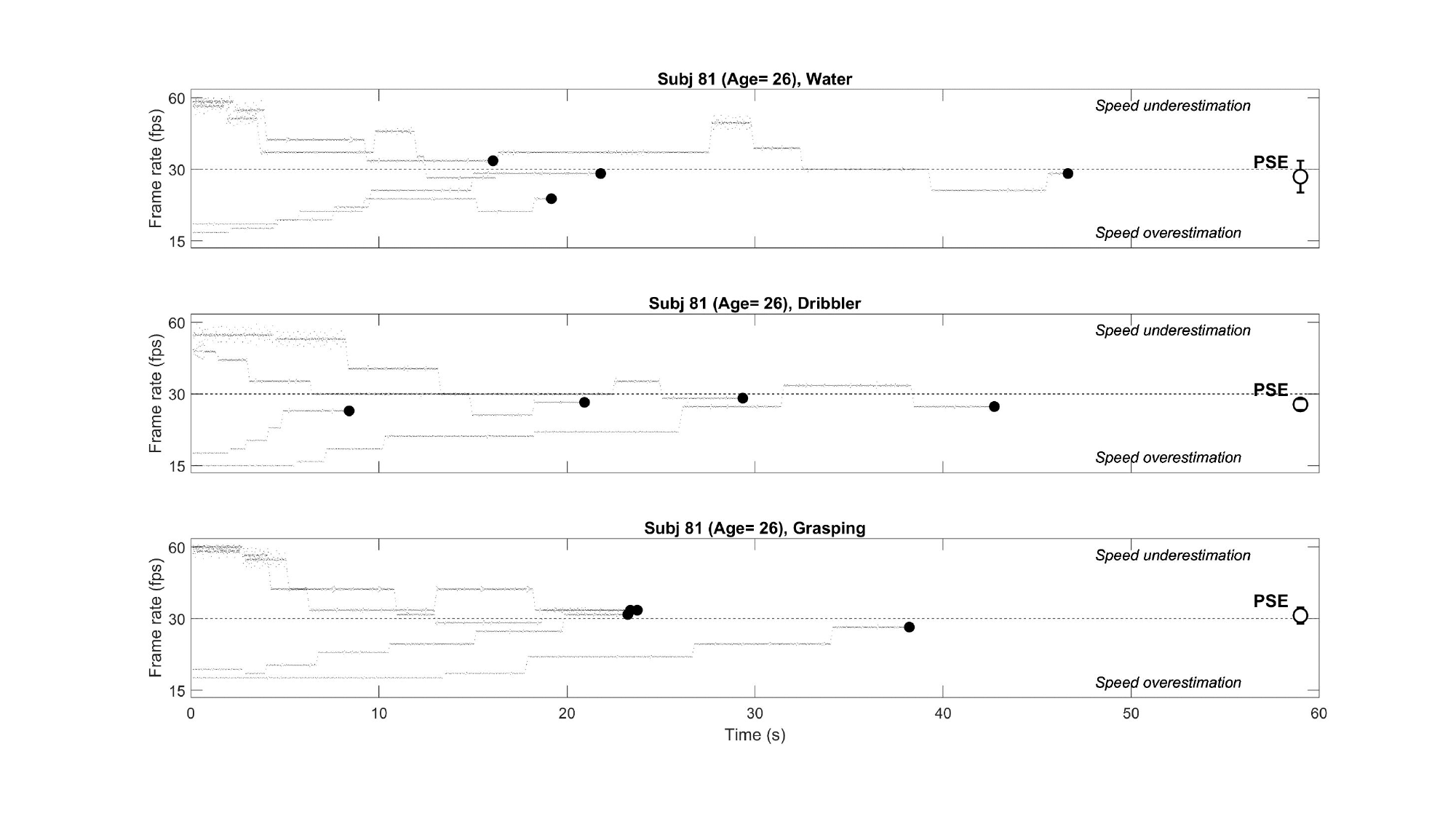

#

## Slide 5
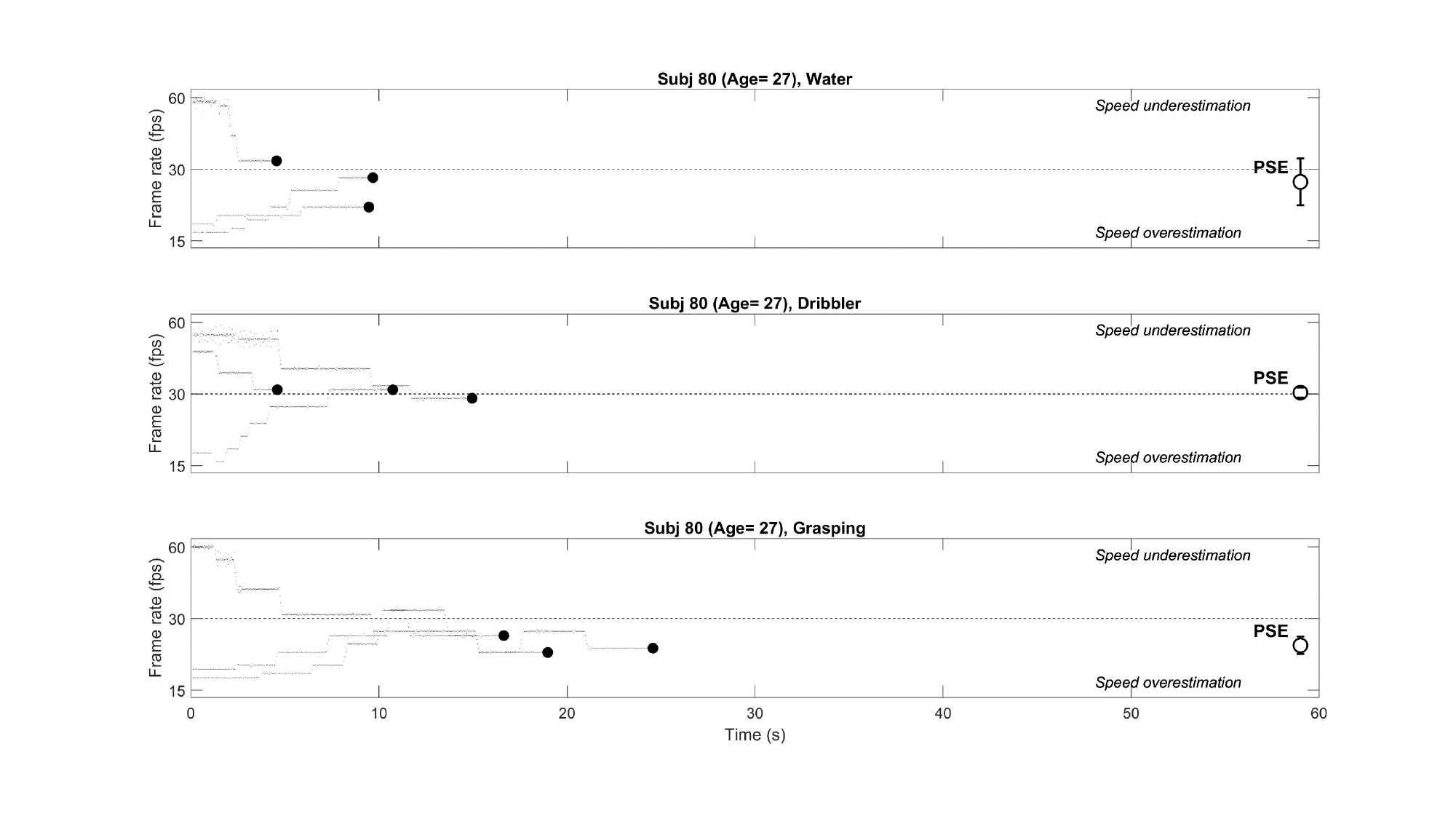

#

## Slide 6
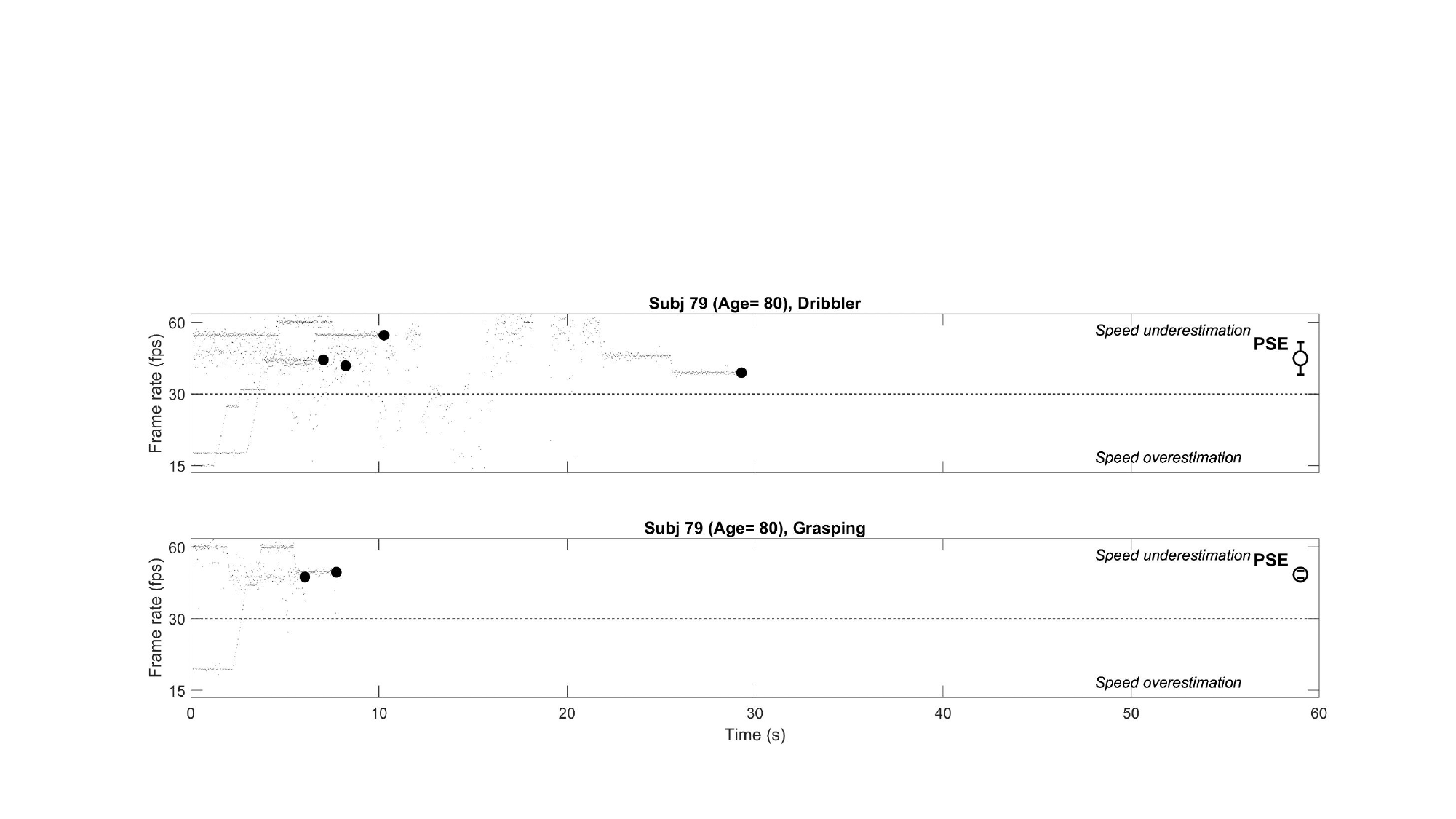

#

## Slide 7
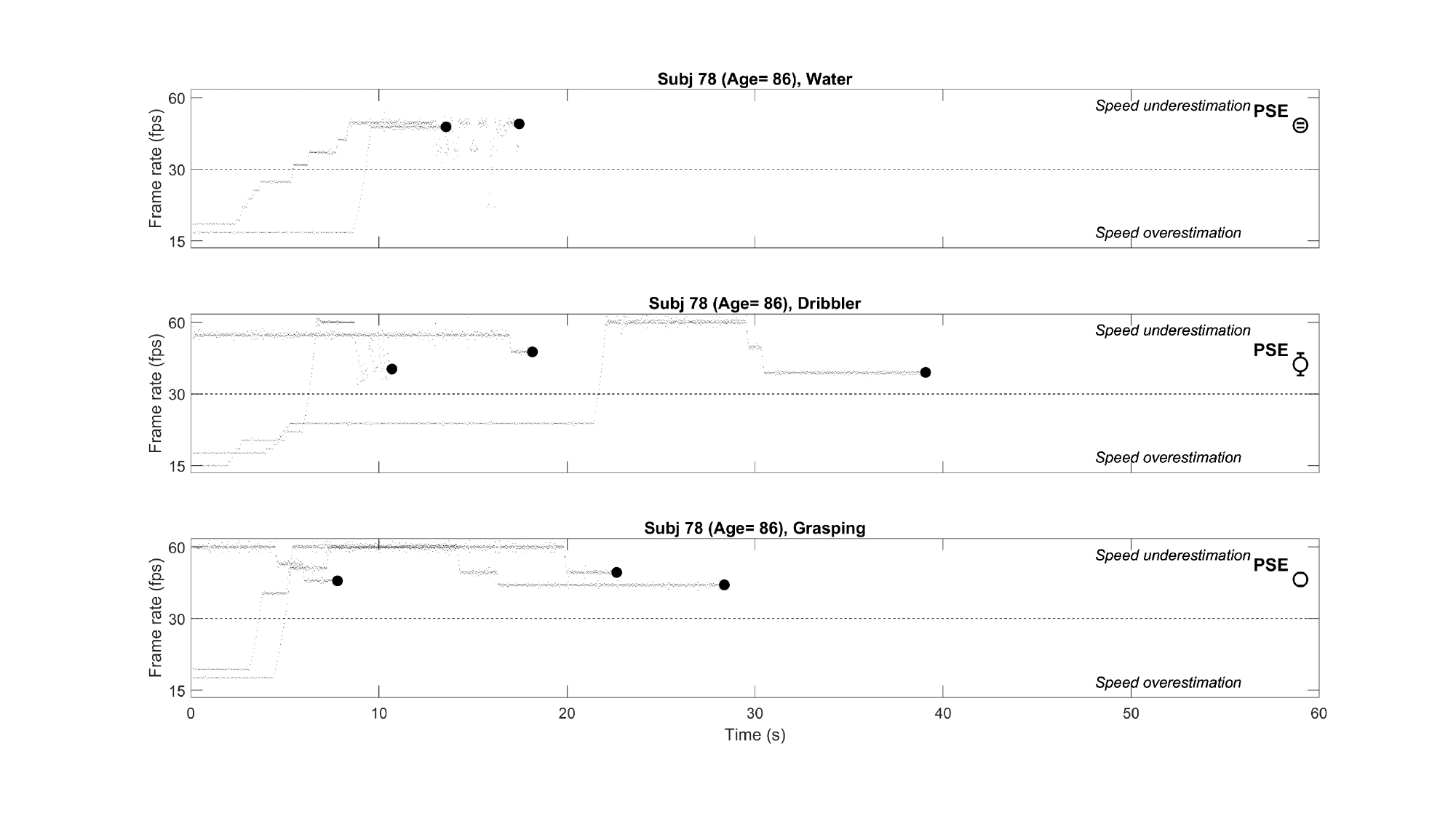

#

## Slide 8
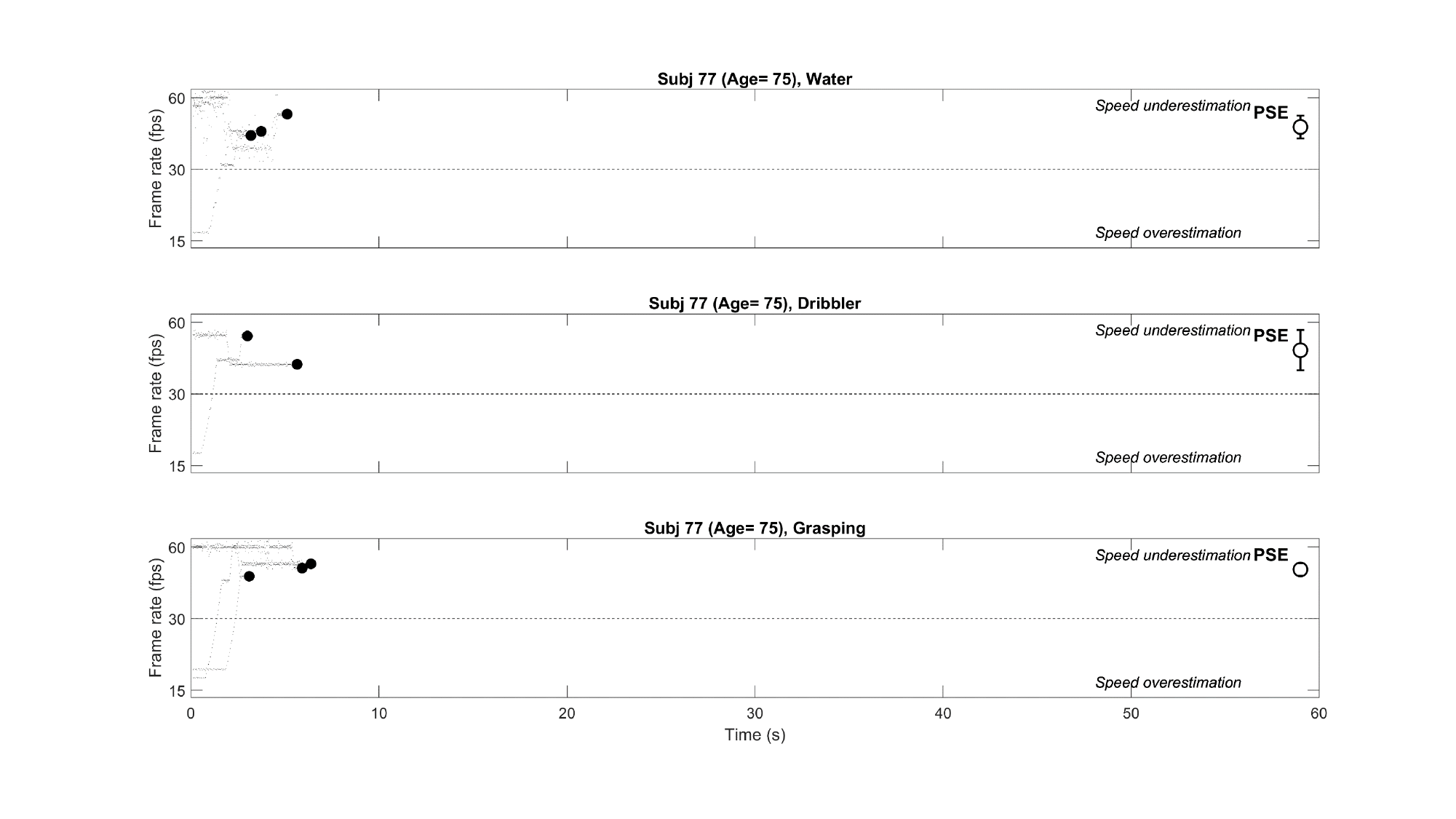

#

## Slide 9
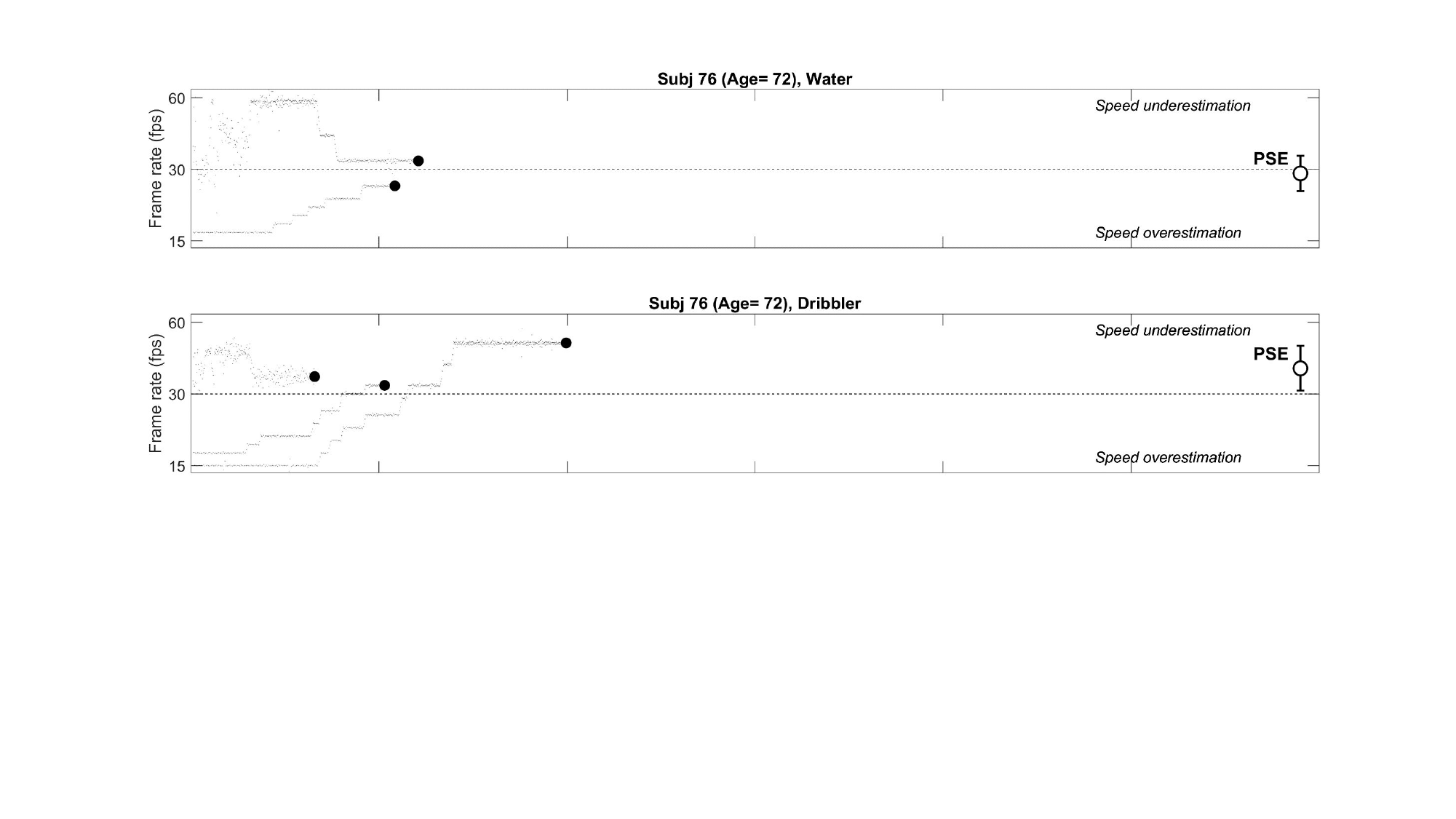

#

## Slide 10
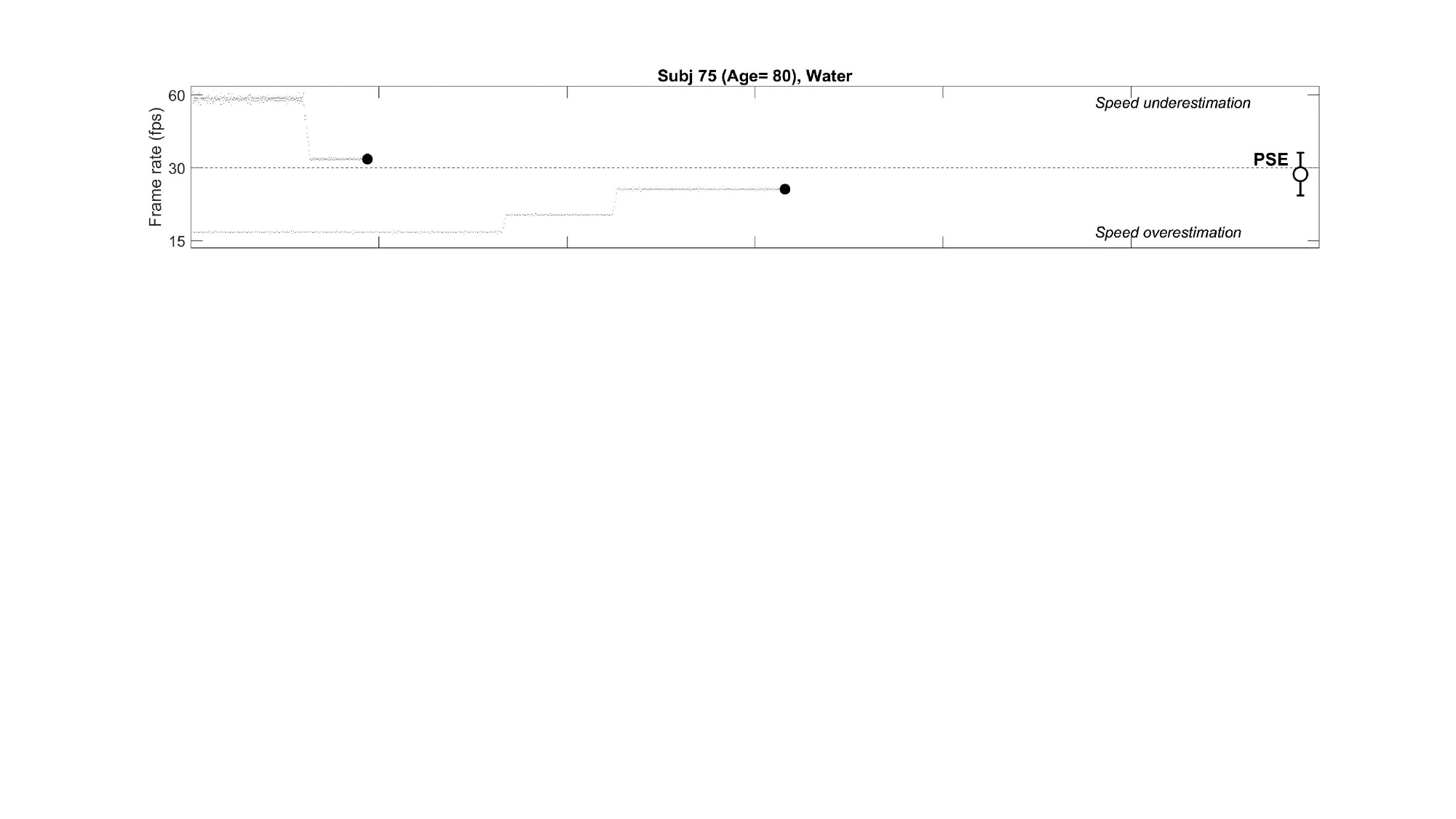

#

## Slide 11
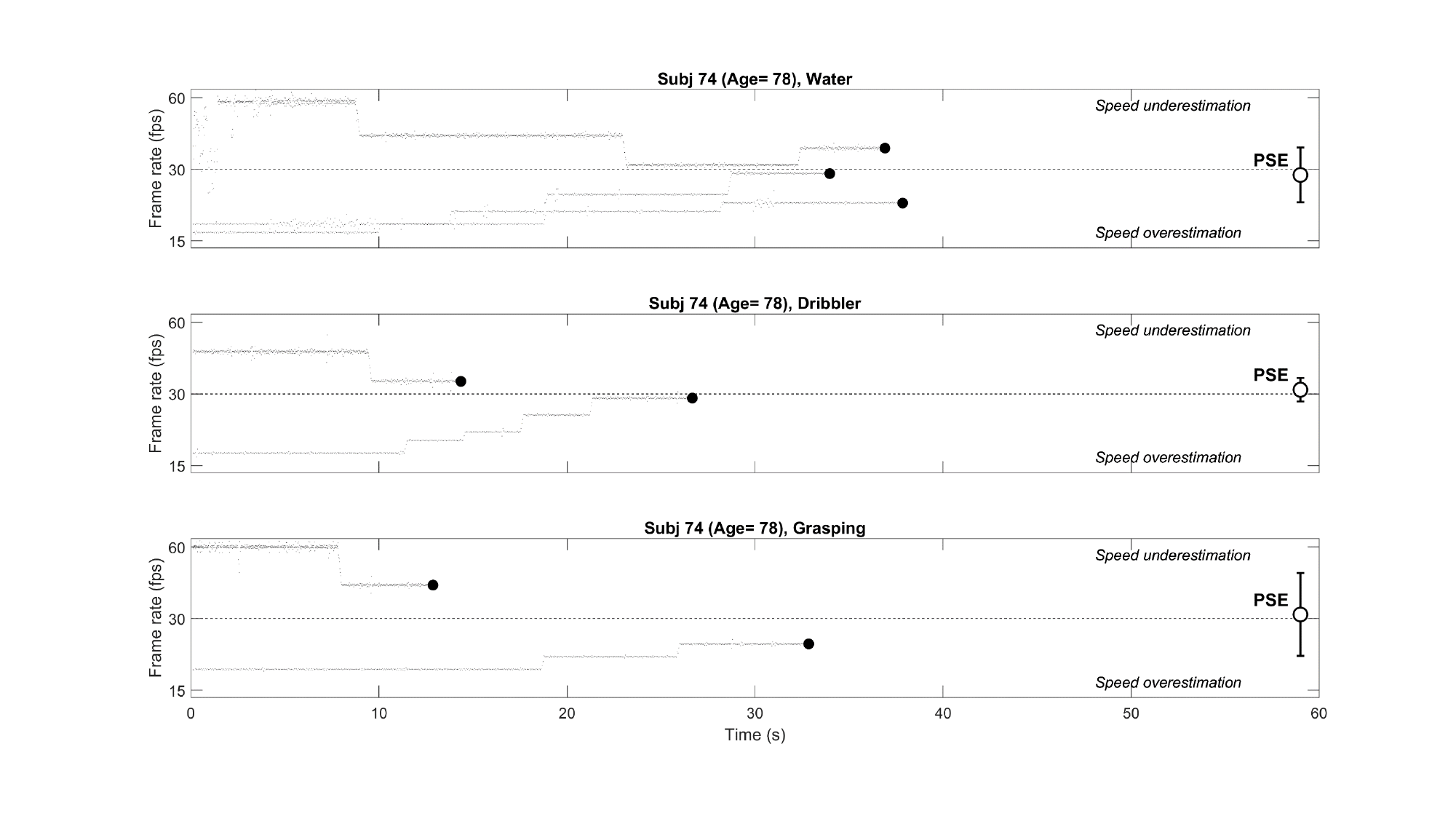

#

## Slide 12
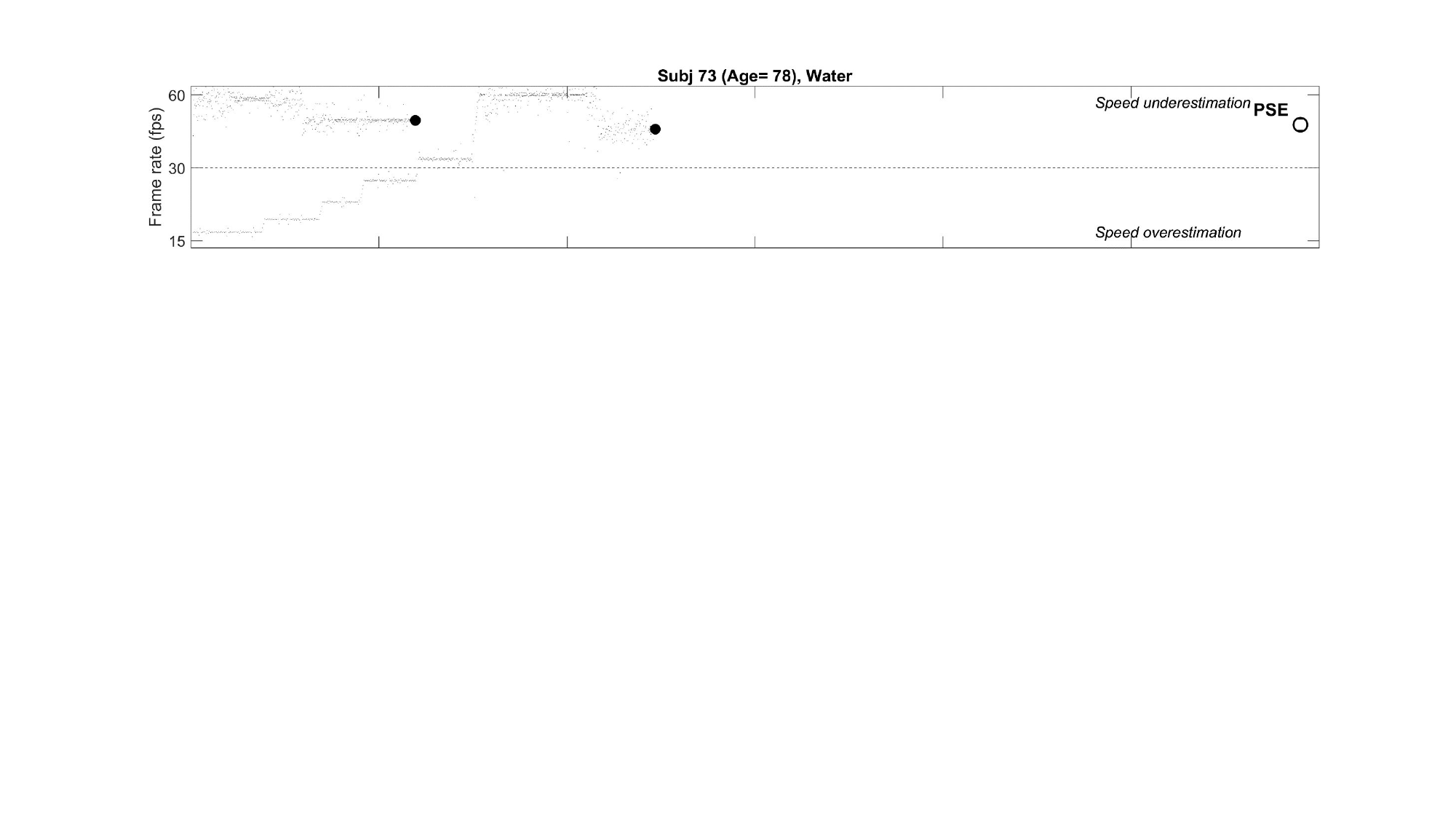

#

## Slide 13
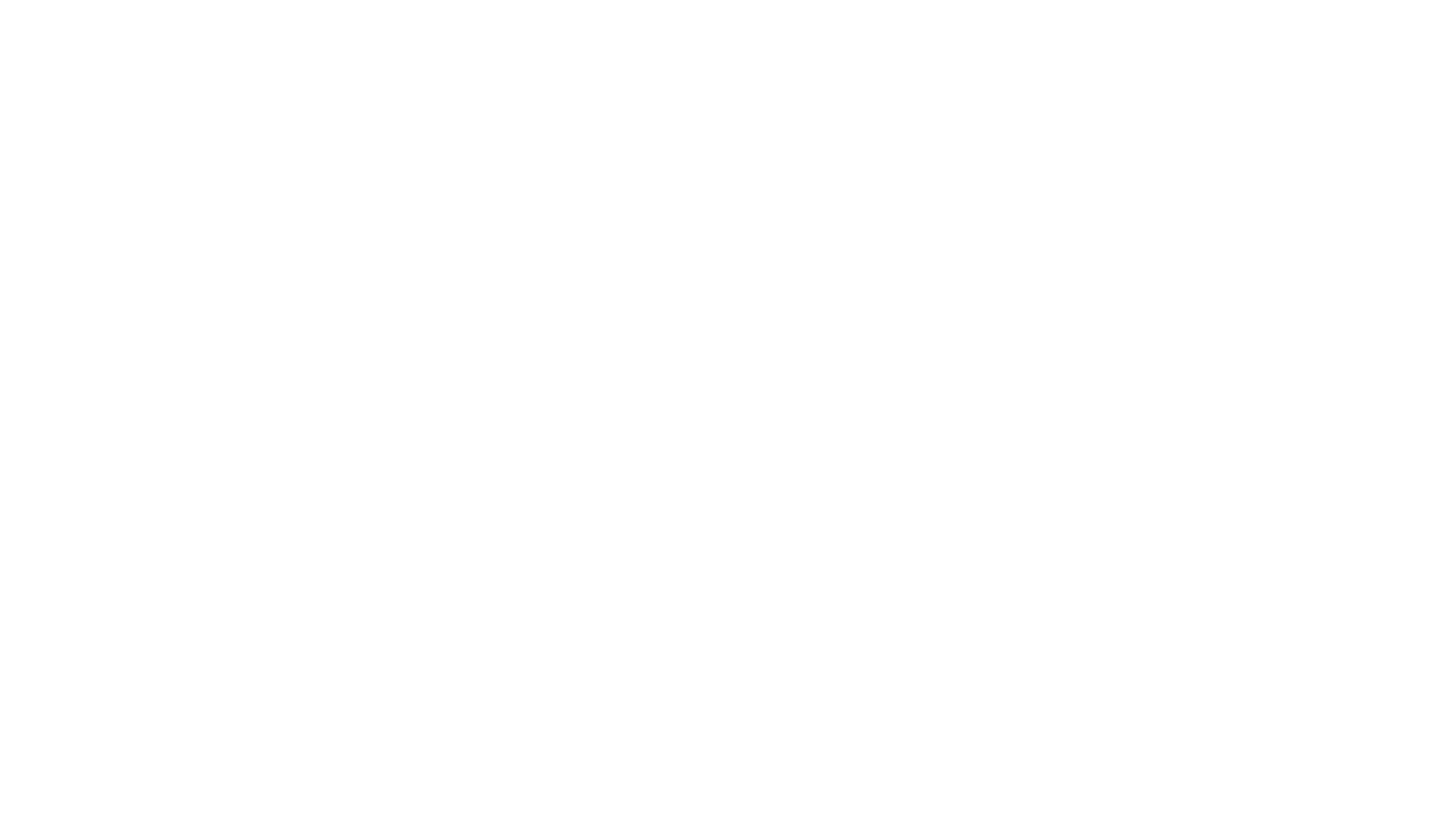

#

## Slide 14
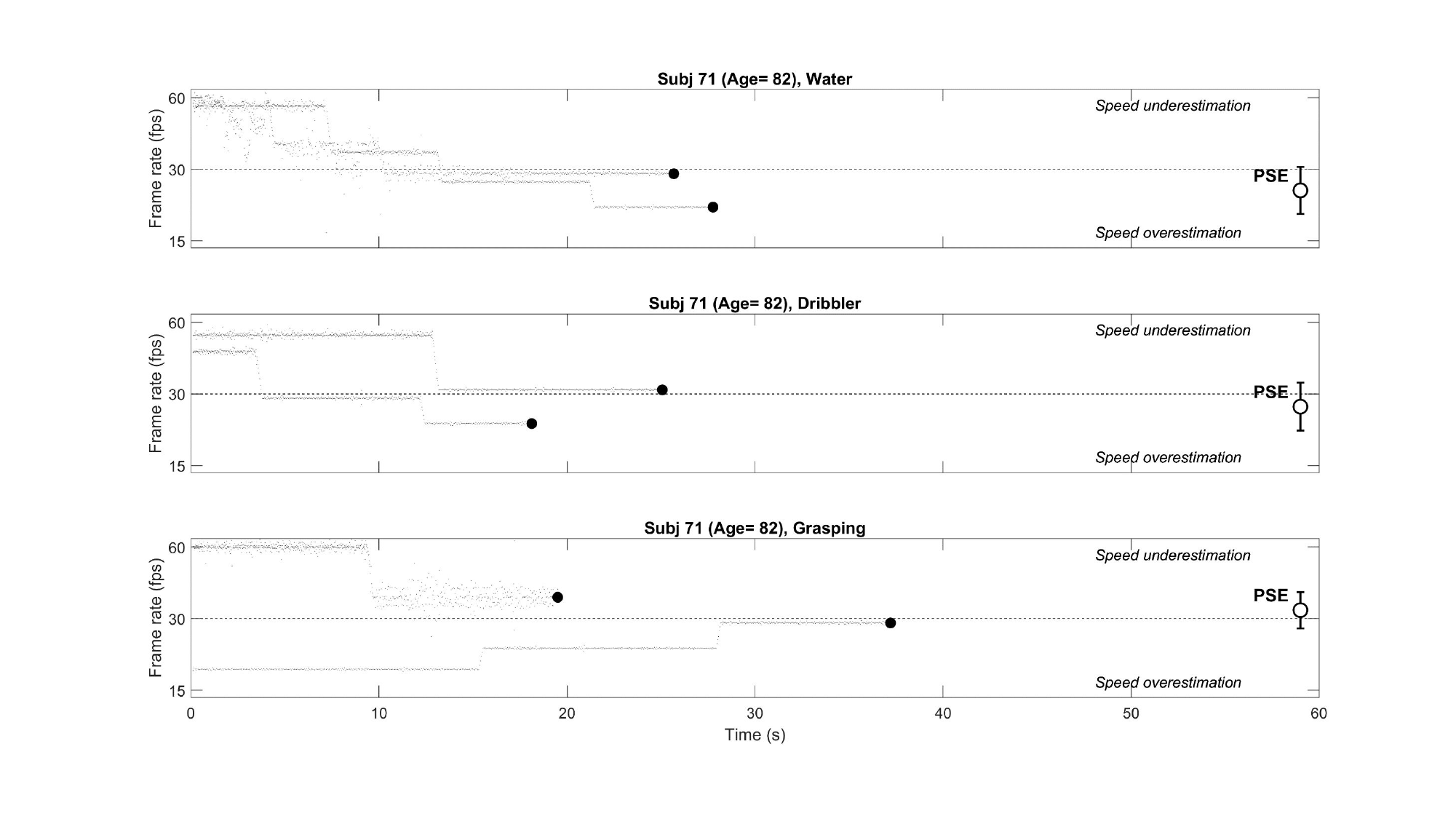

#

## Slide 15
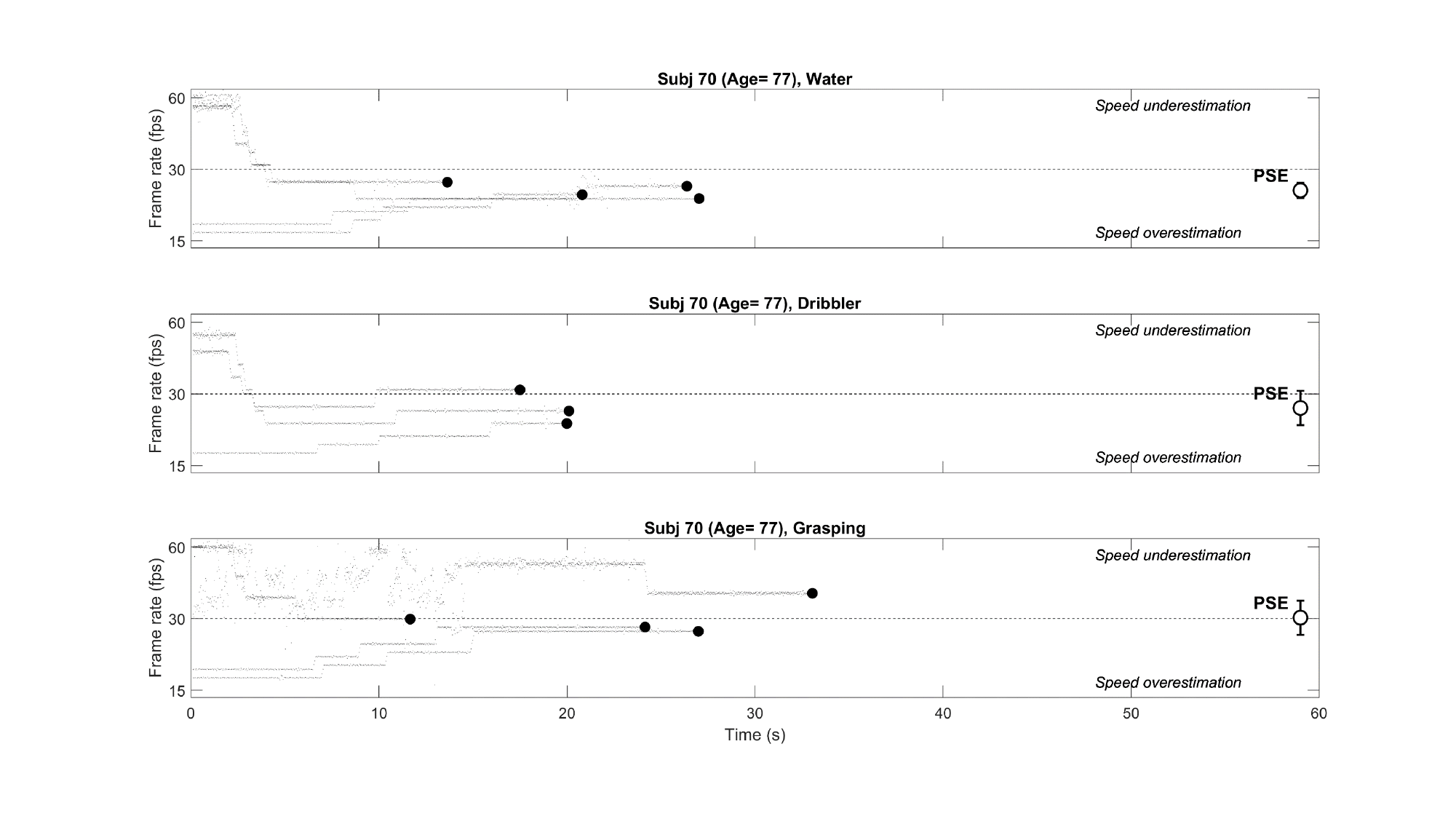

#

## Slide 16
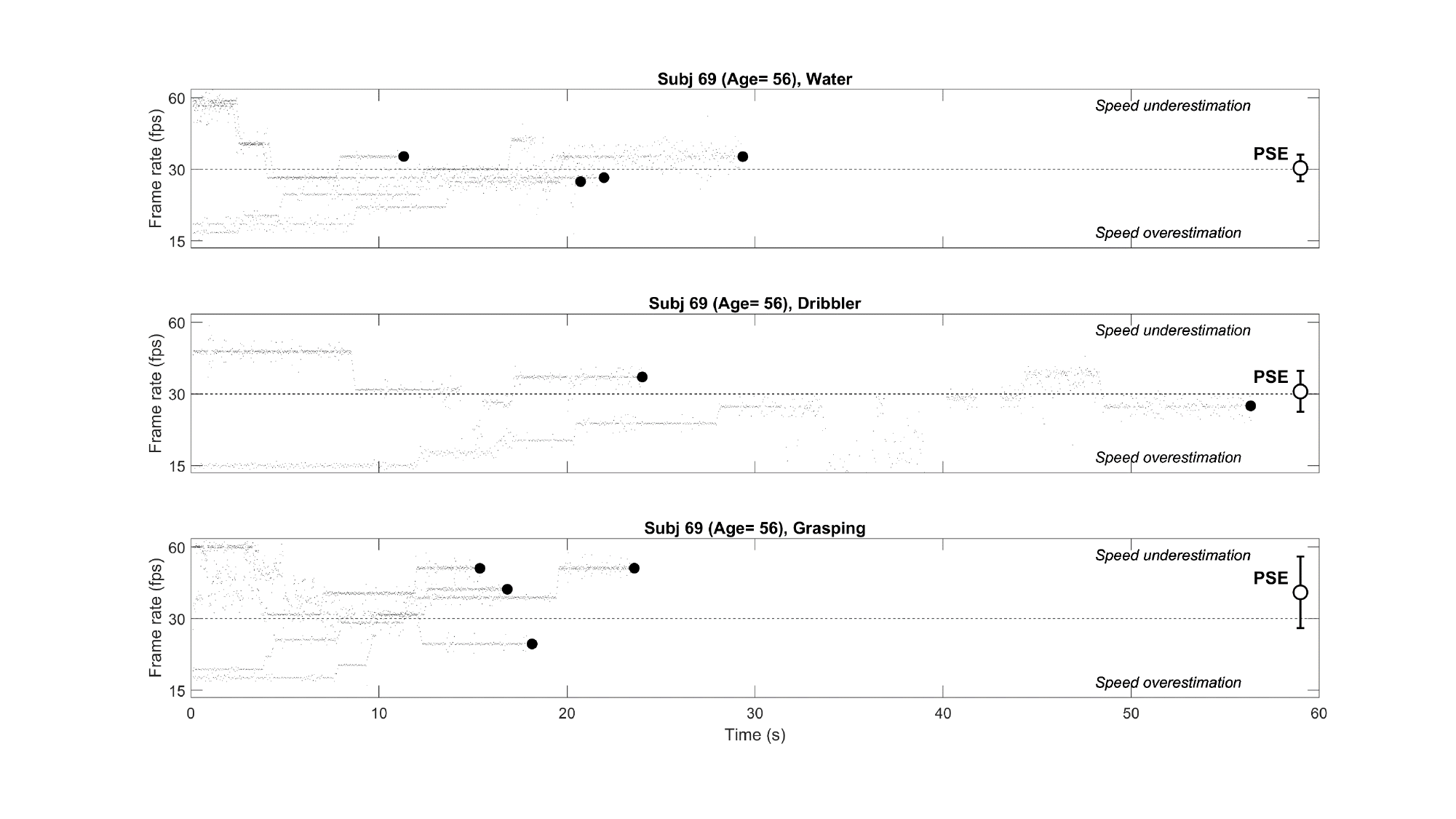

#

## Slide 17
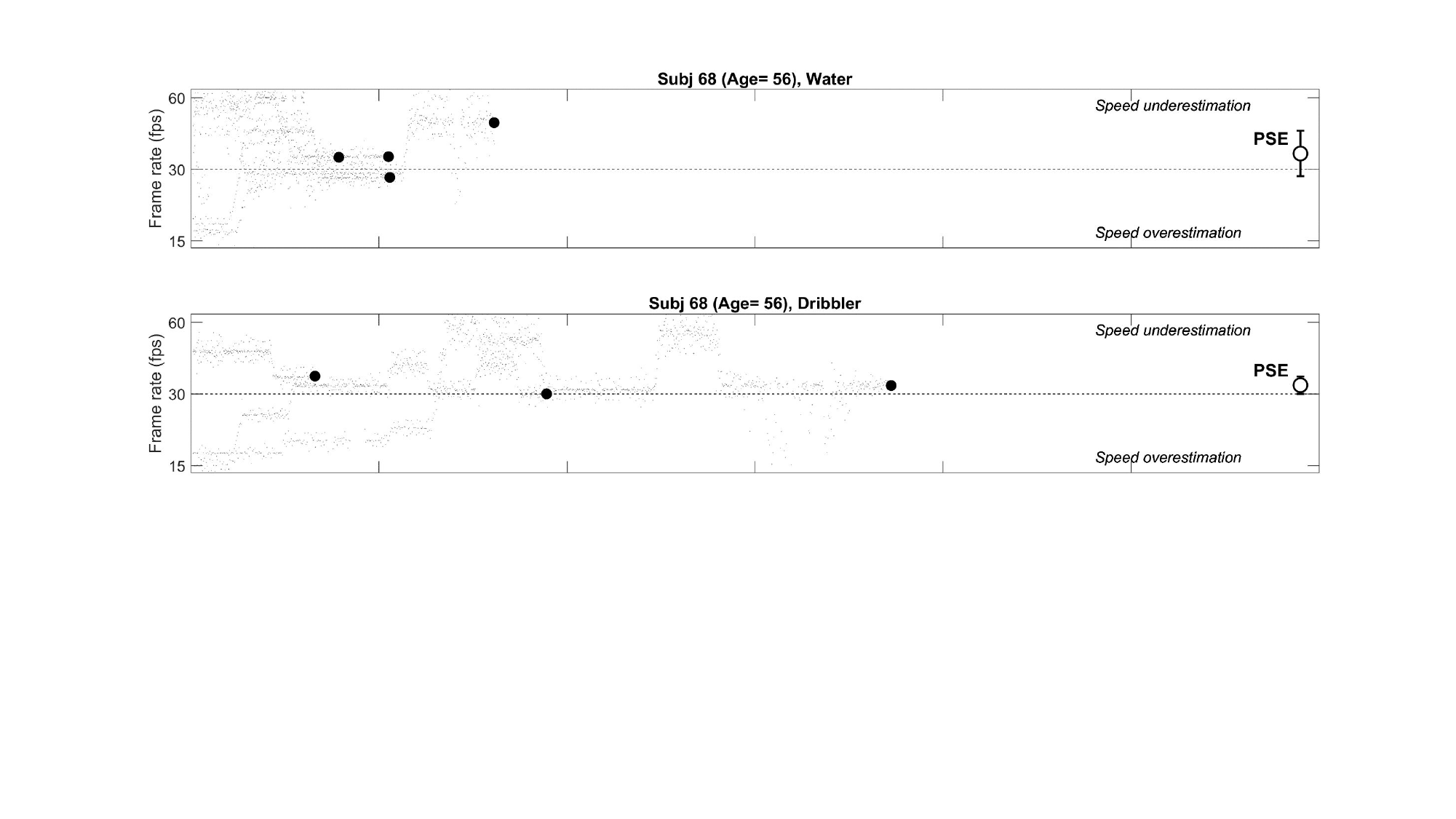

#

## Slide 18
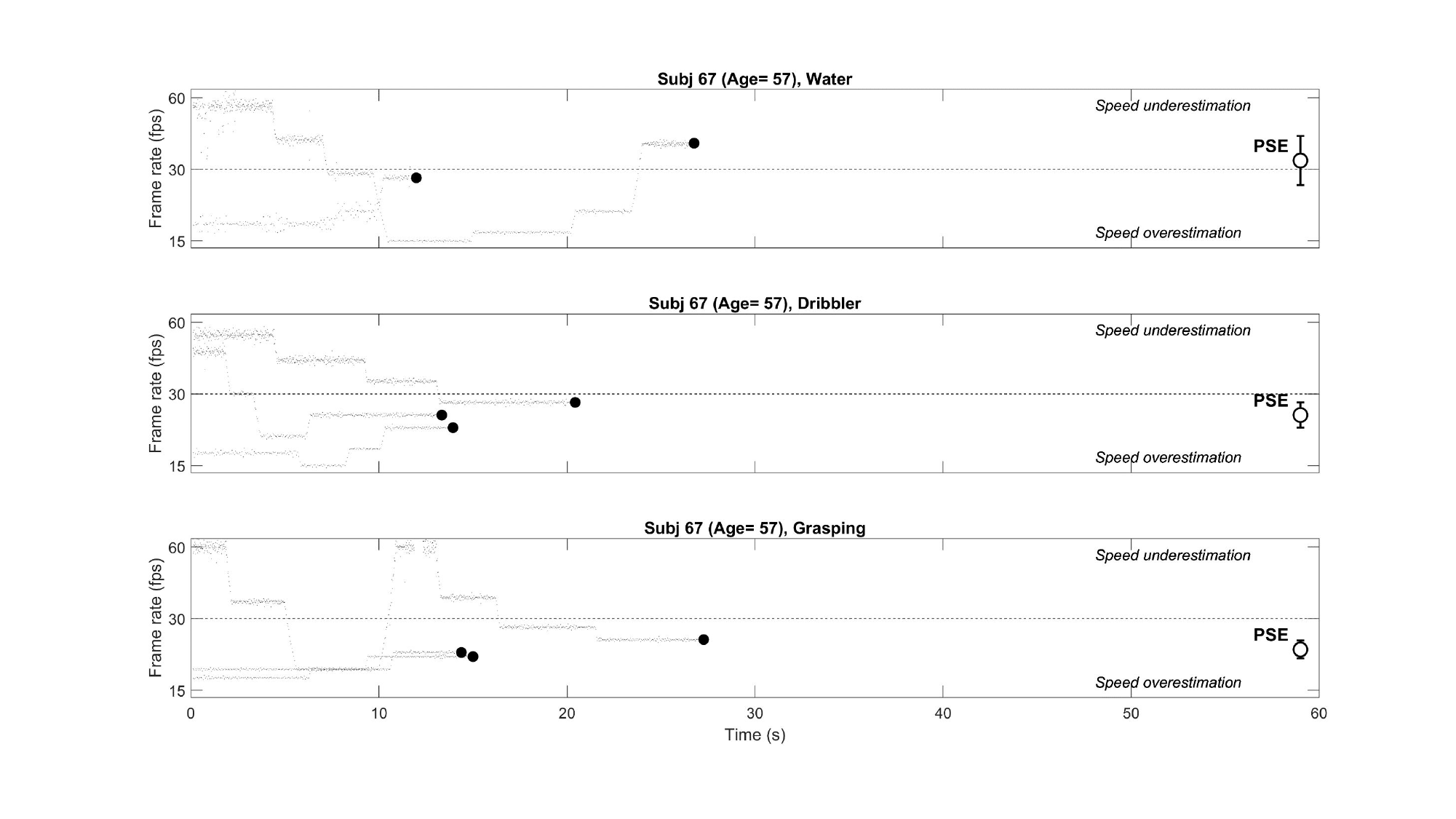

#

## Slide 19
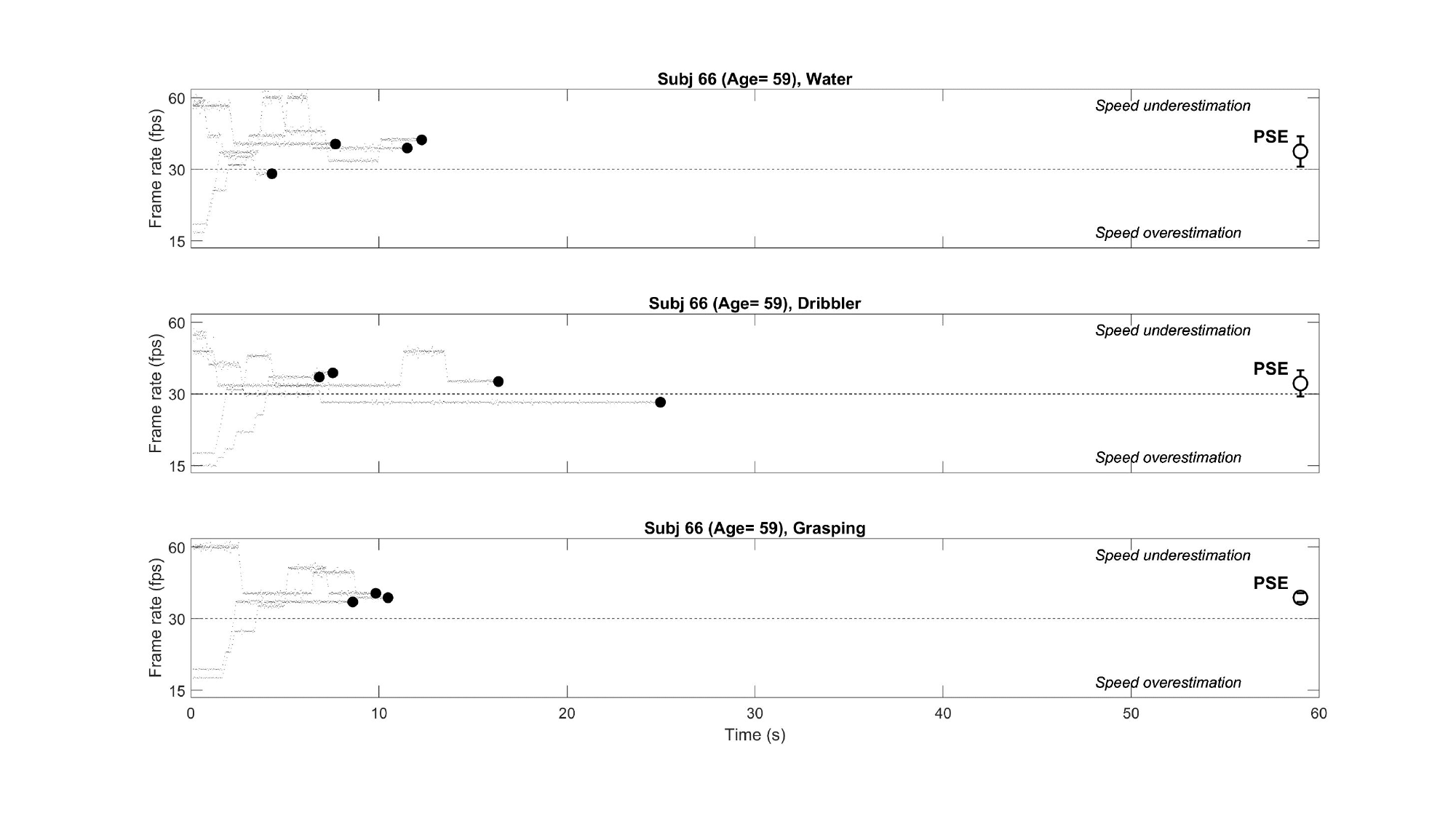

#

## Slide 20
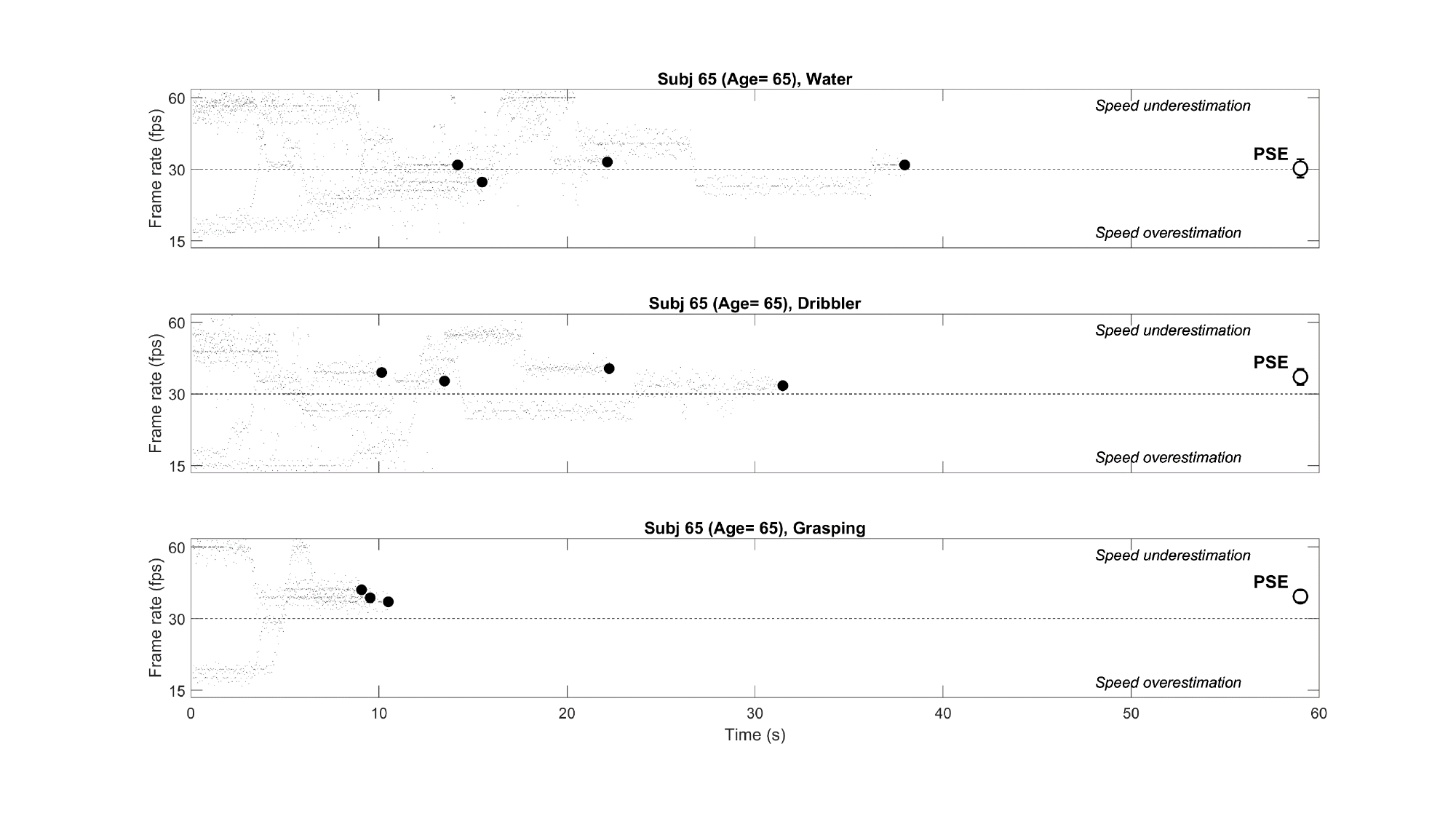

#

## Slide 21
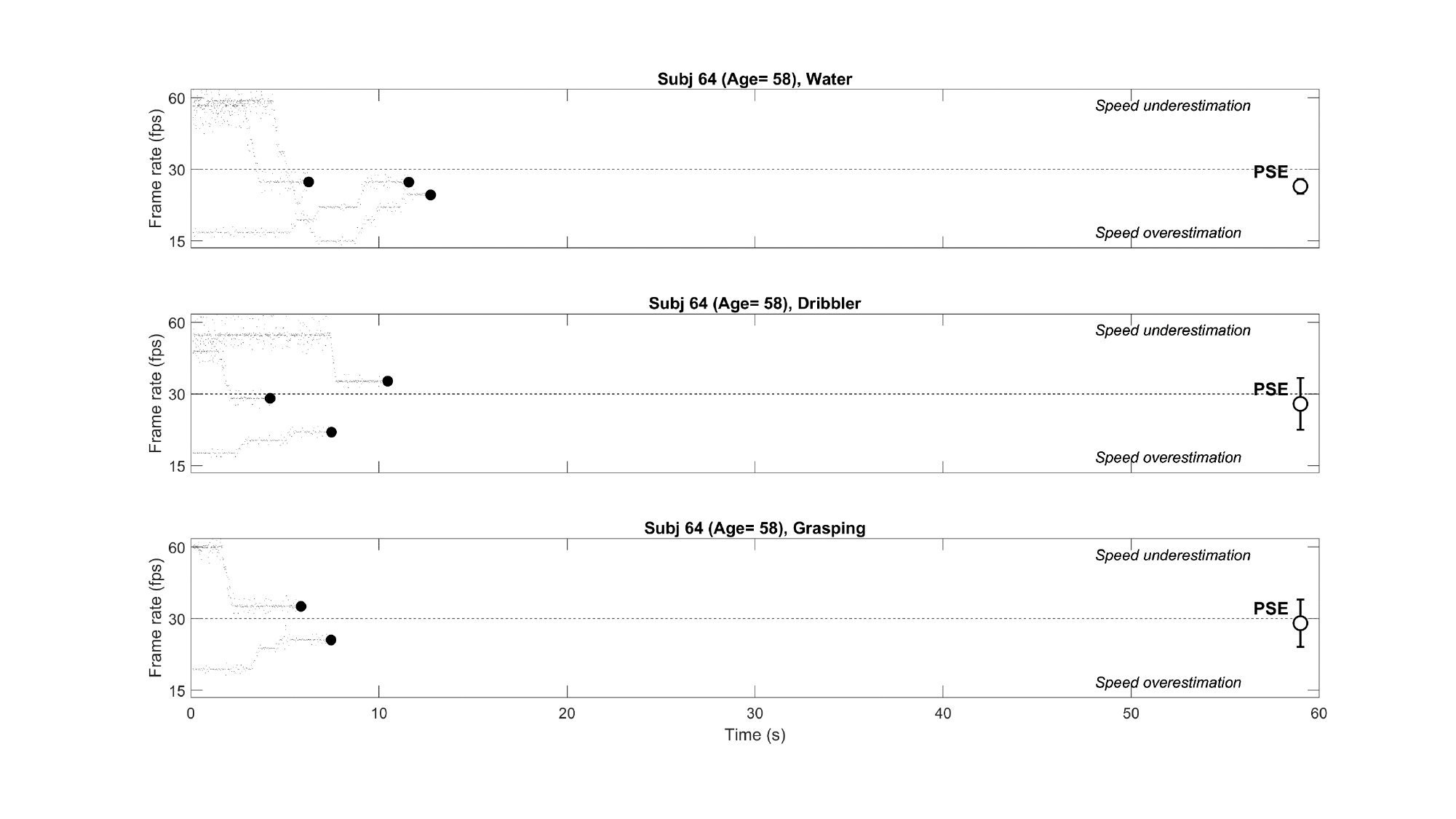

#

## Slide 22
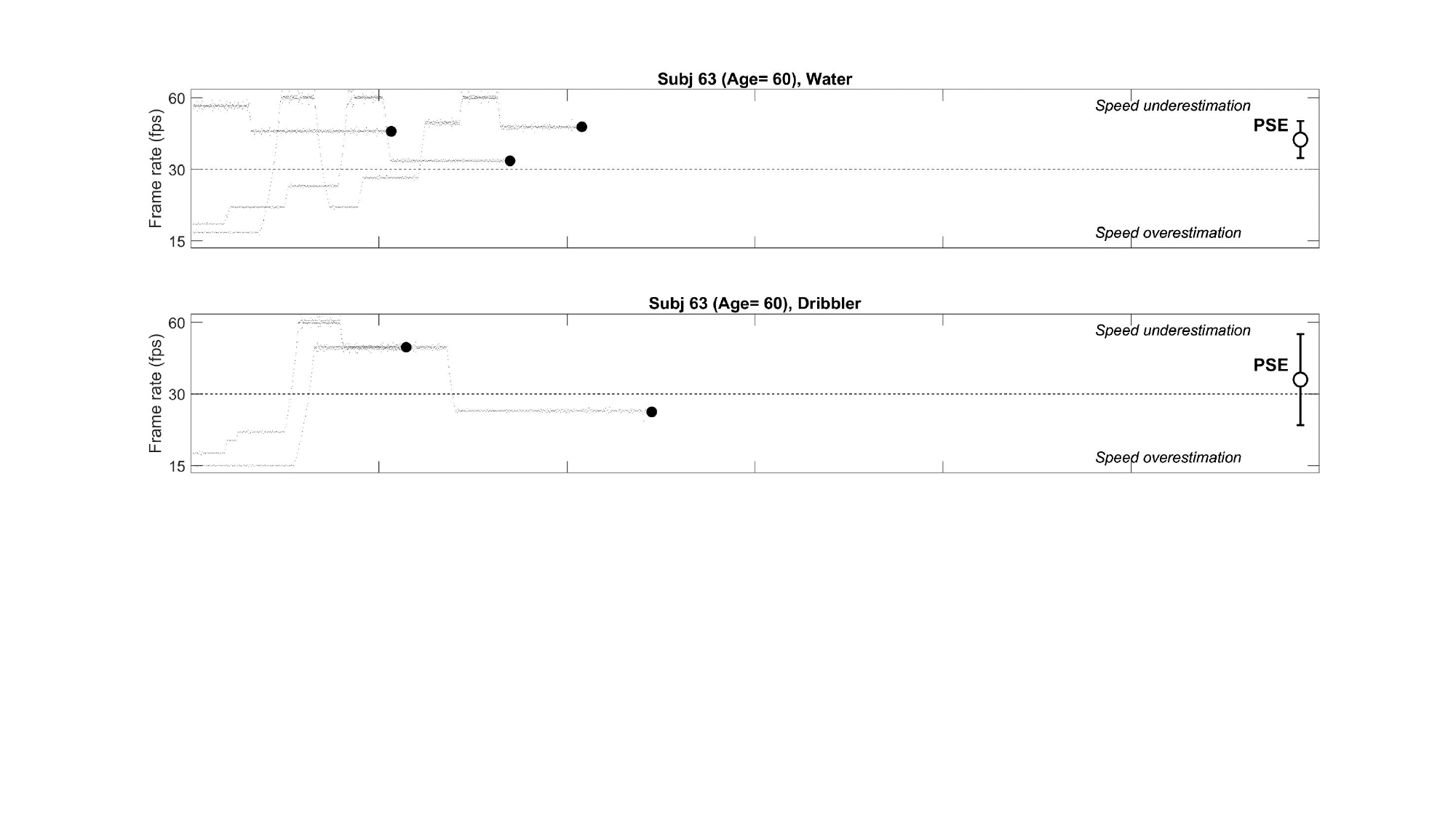

#

## Slide 23
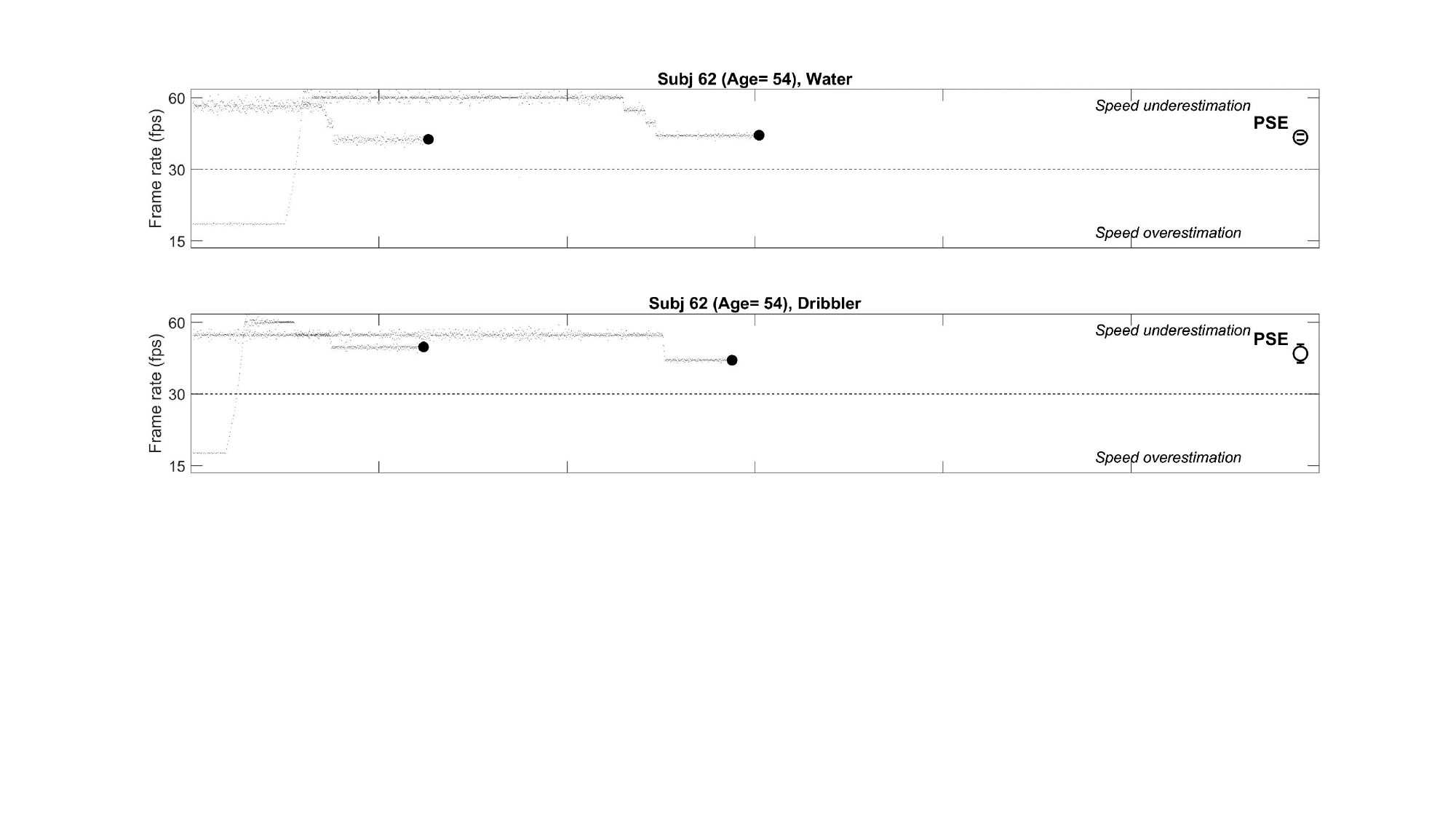

#

## Slide 24
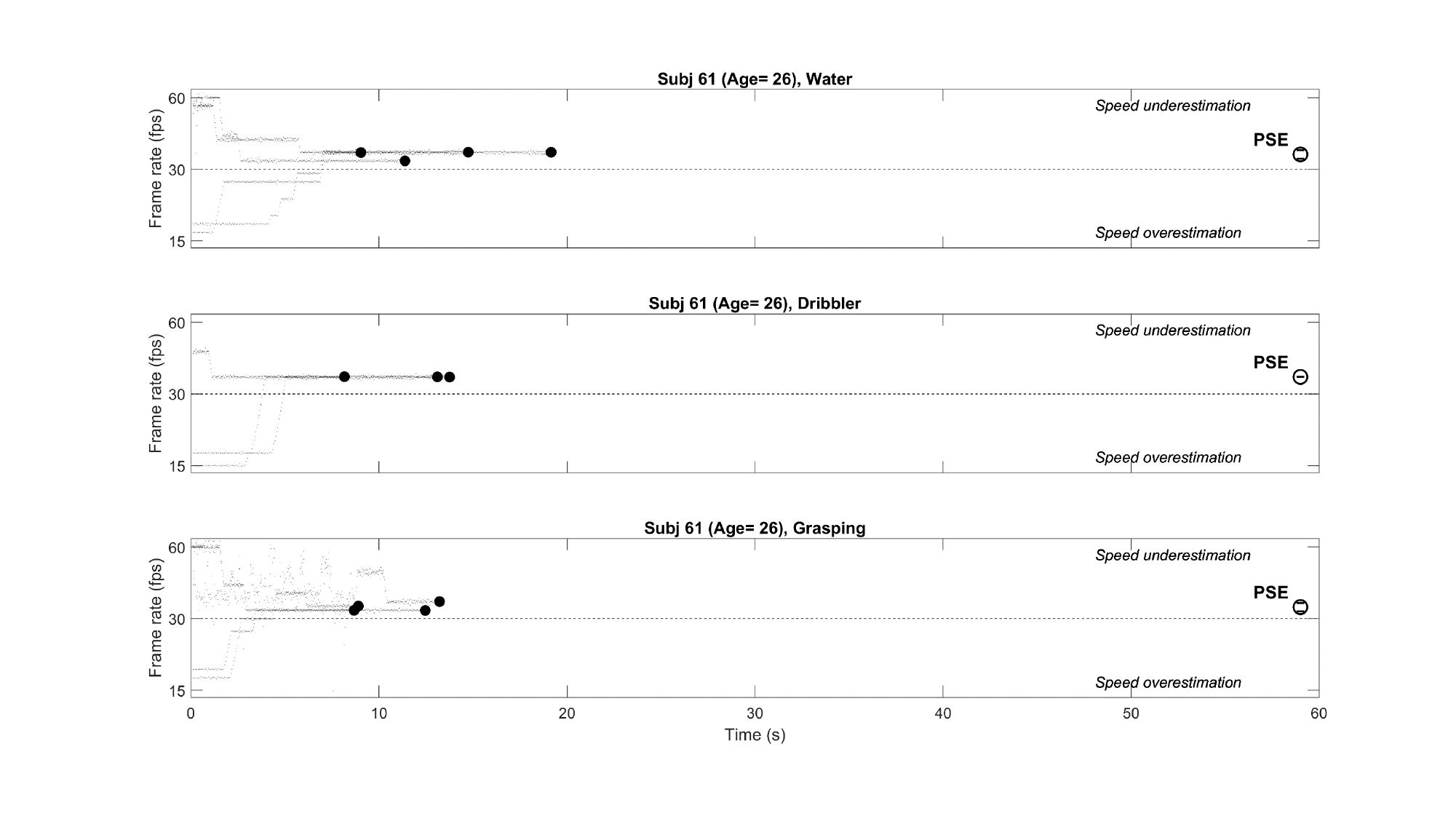

#

## Slide 25
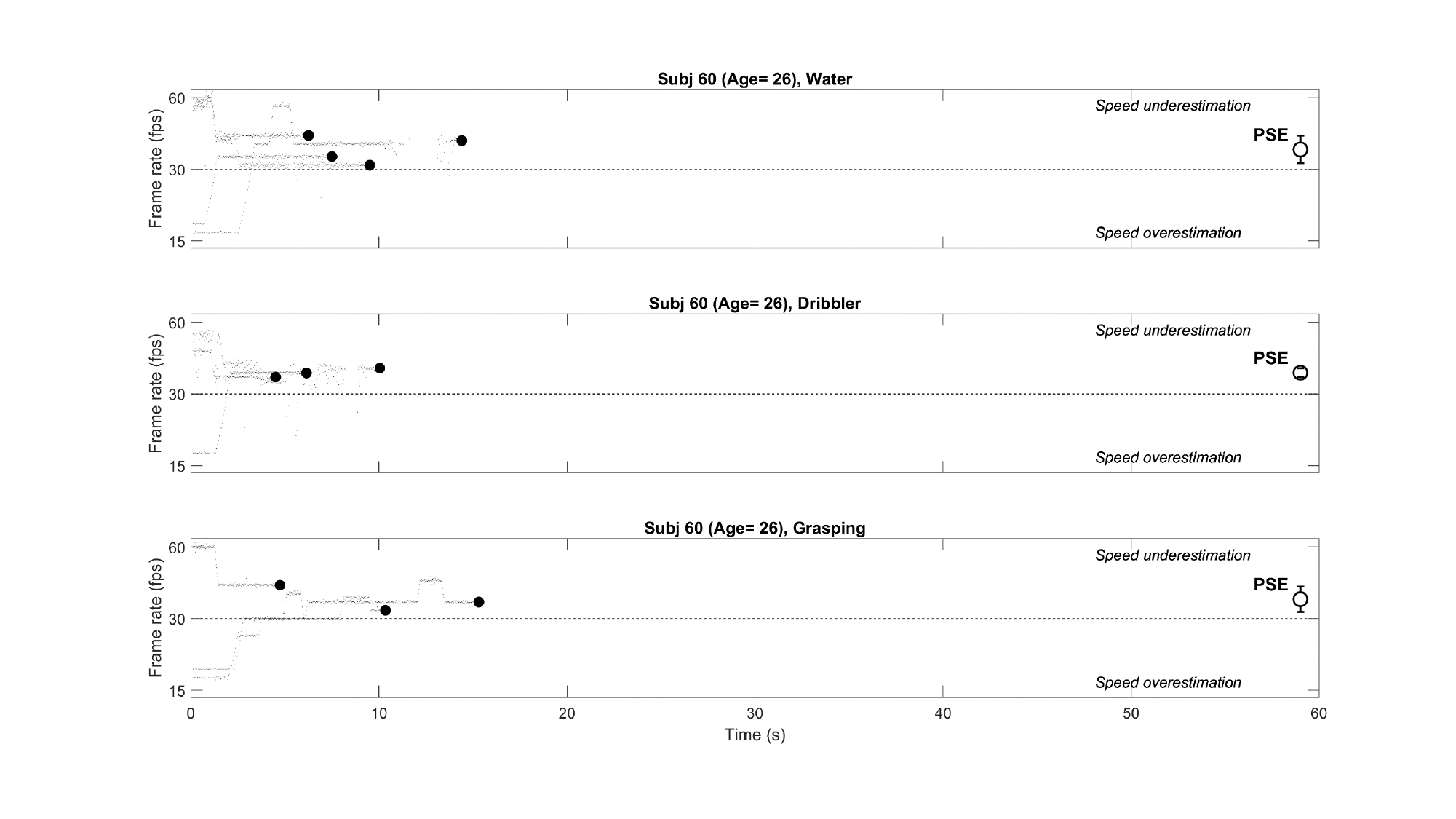

#

## Slide 26
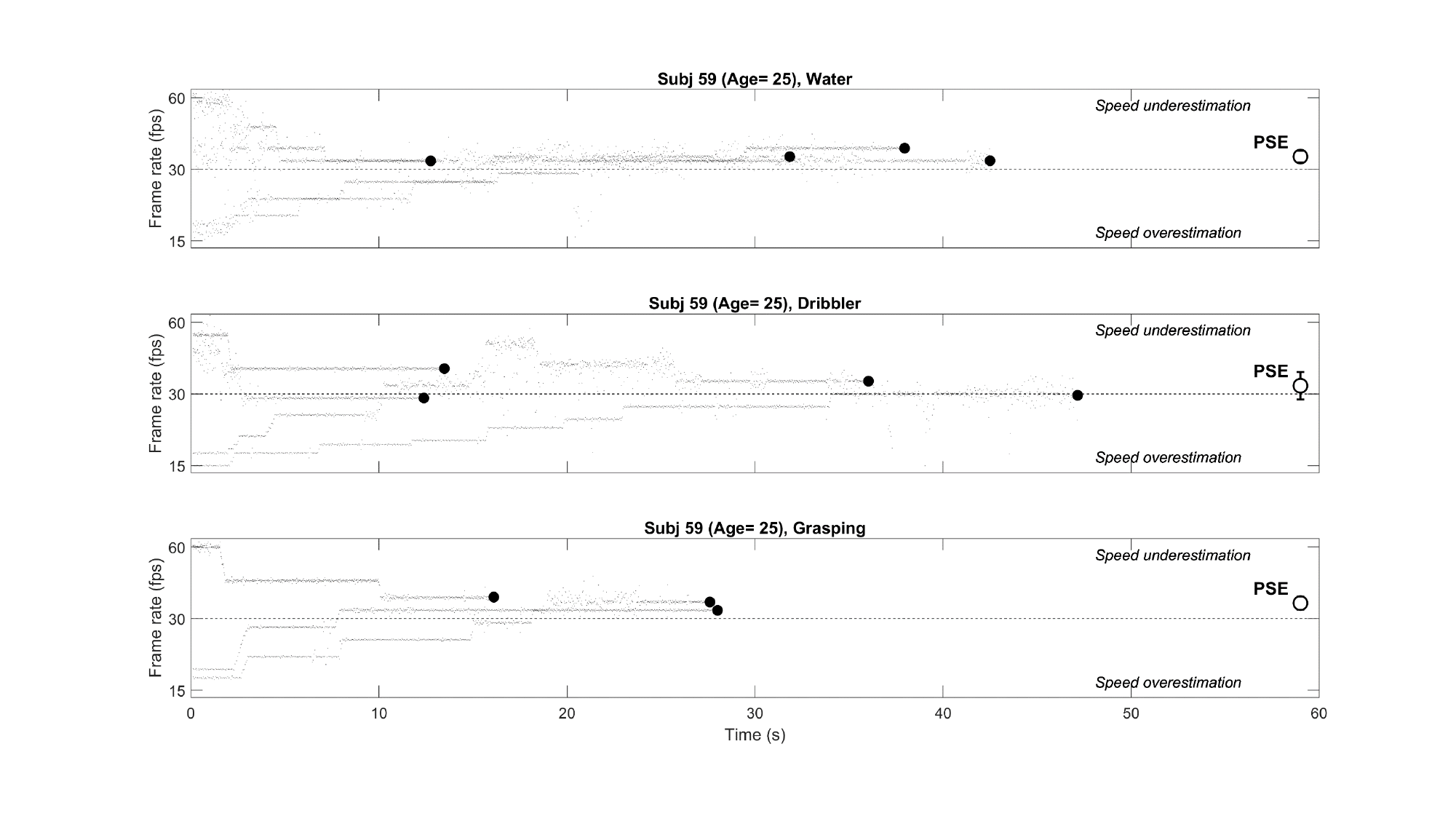

#

## Slide 27
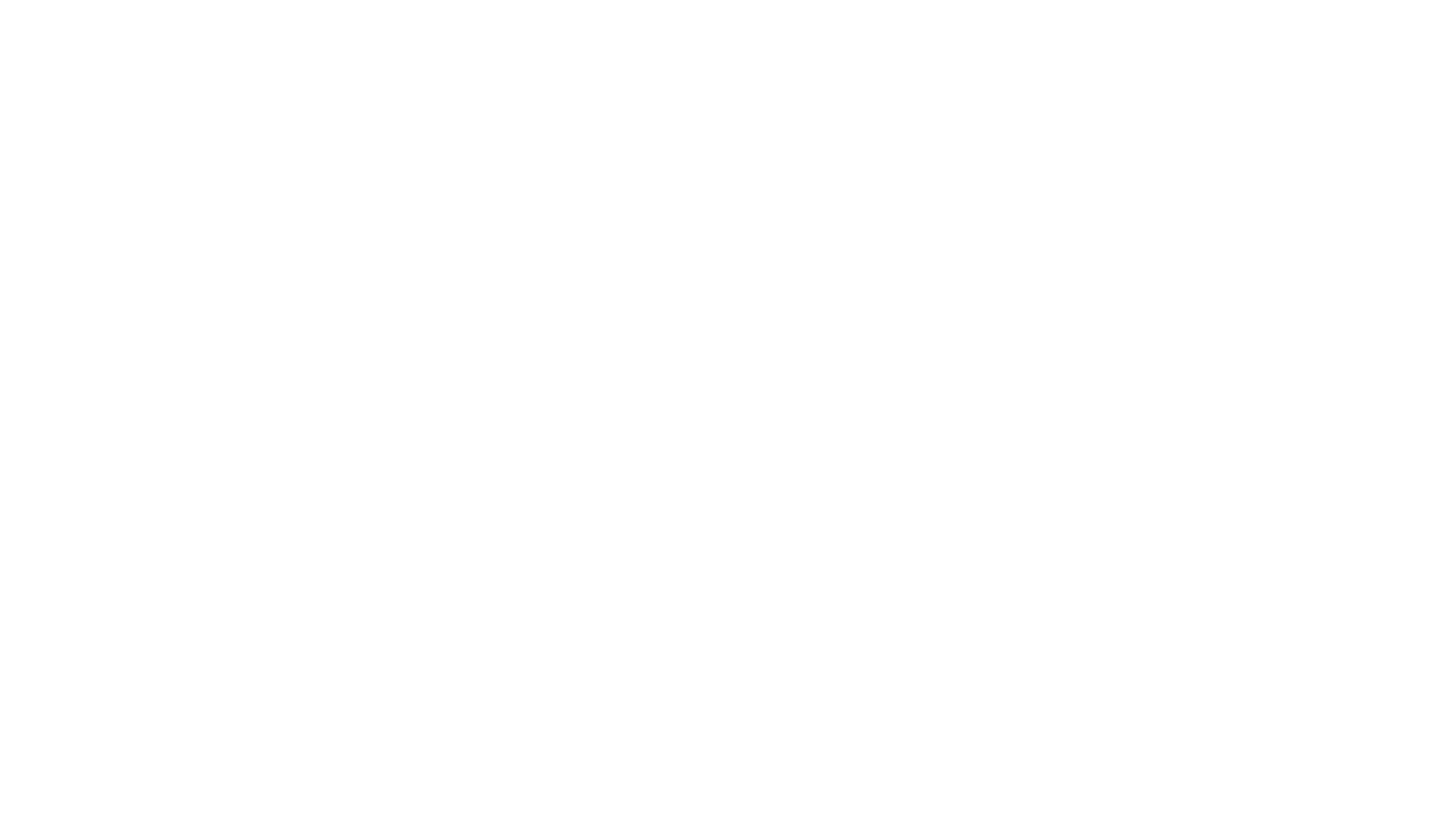

#

## Slide 28
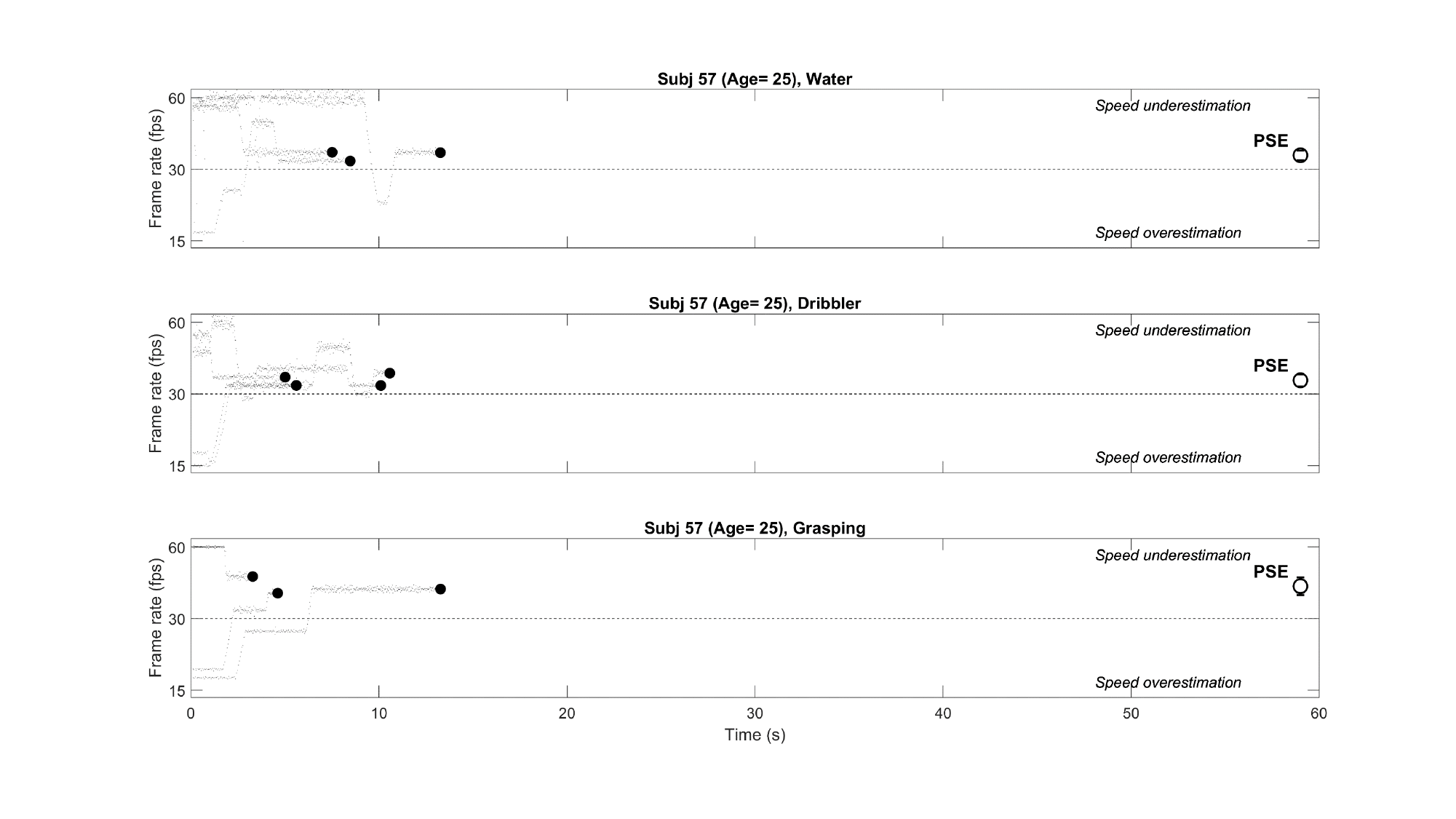

#

## Slide 29
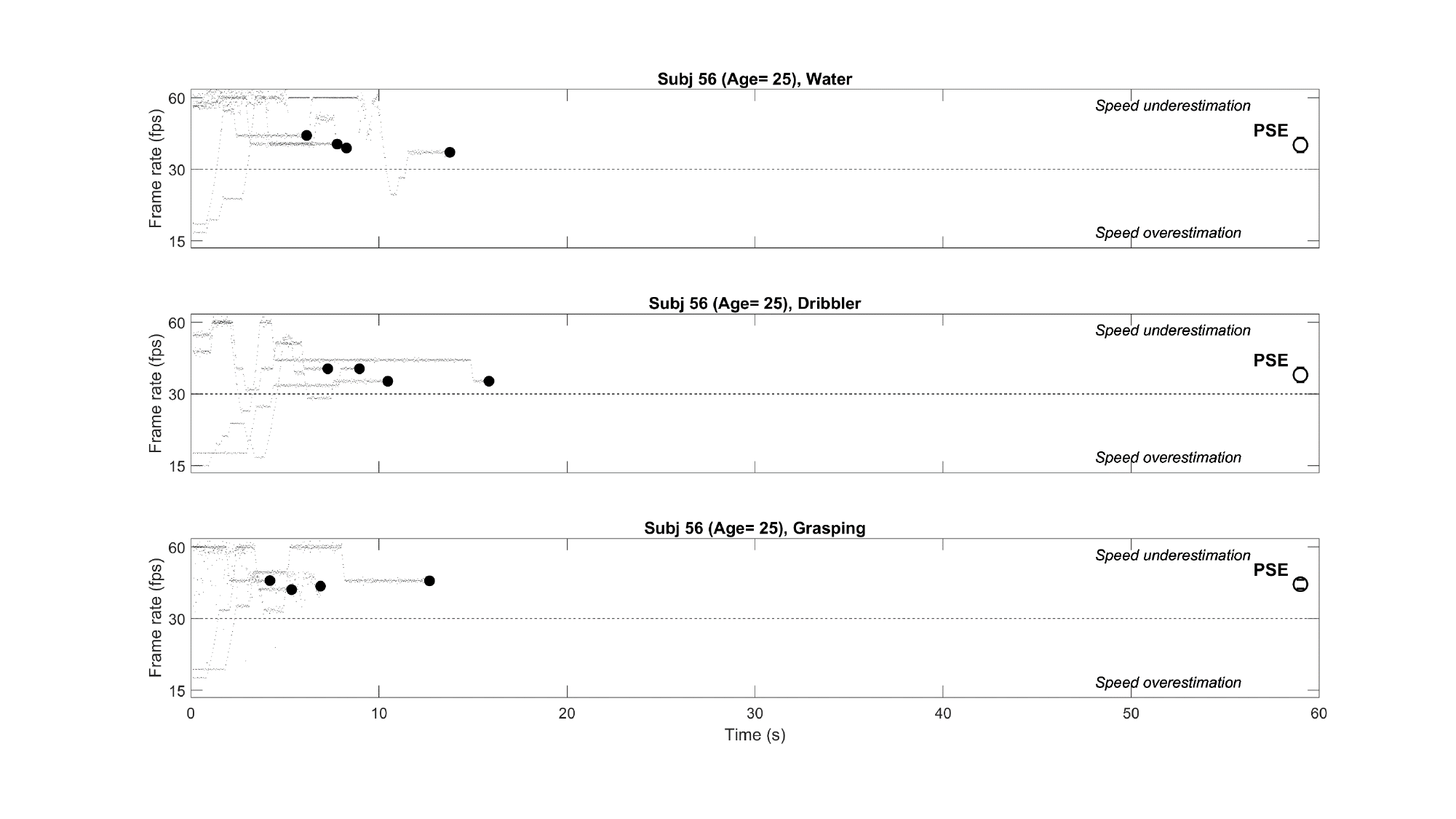

#

## Slide 30
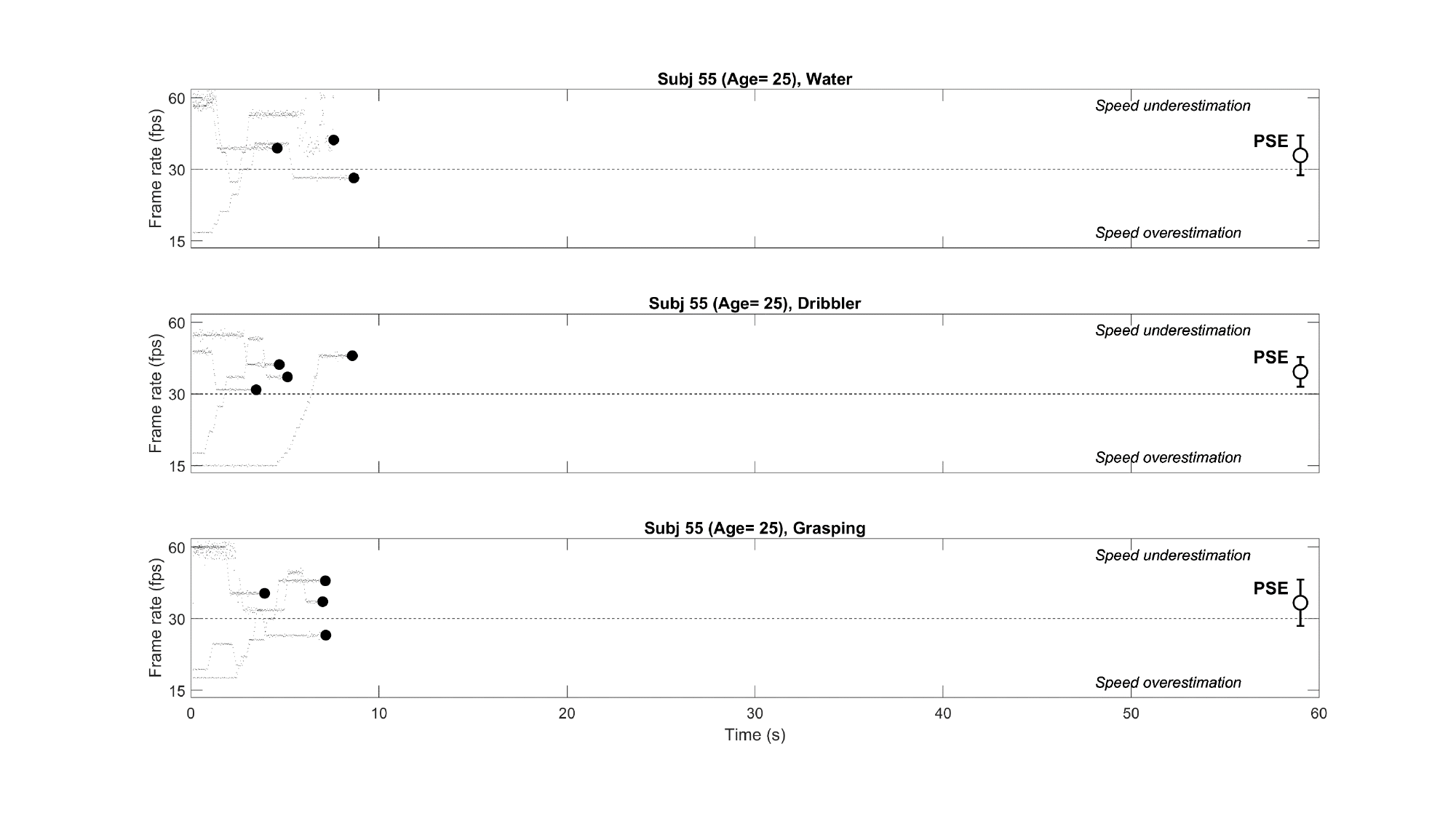

#

## Slide 31
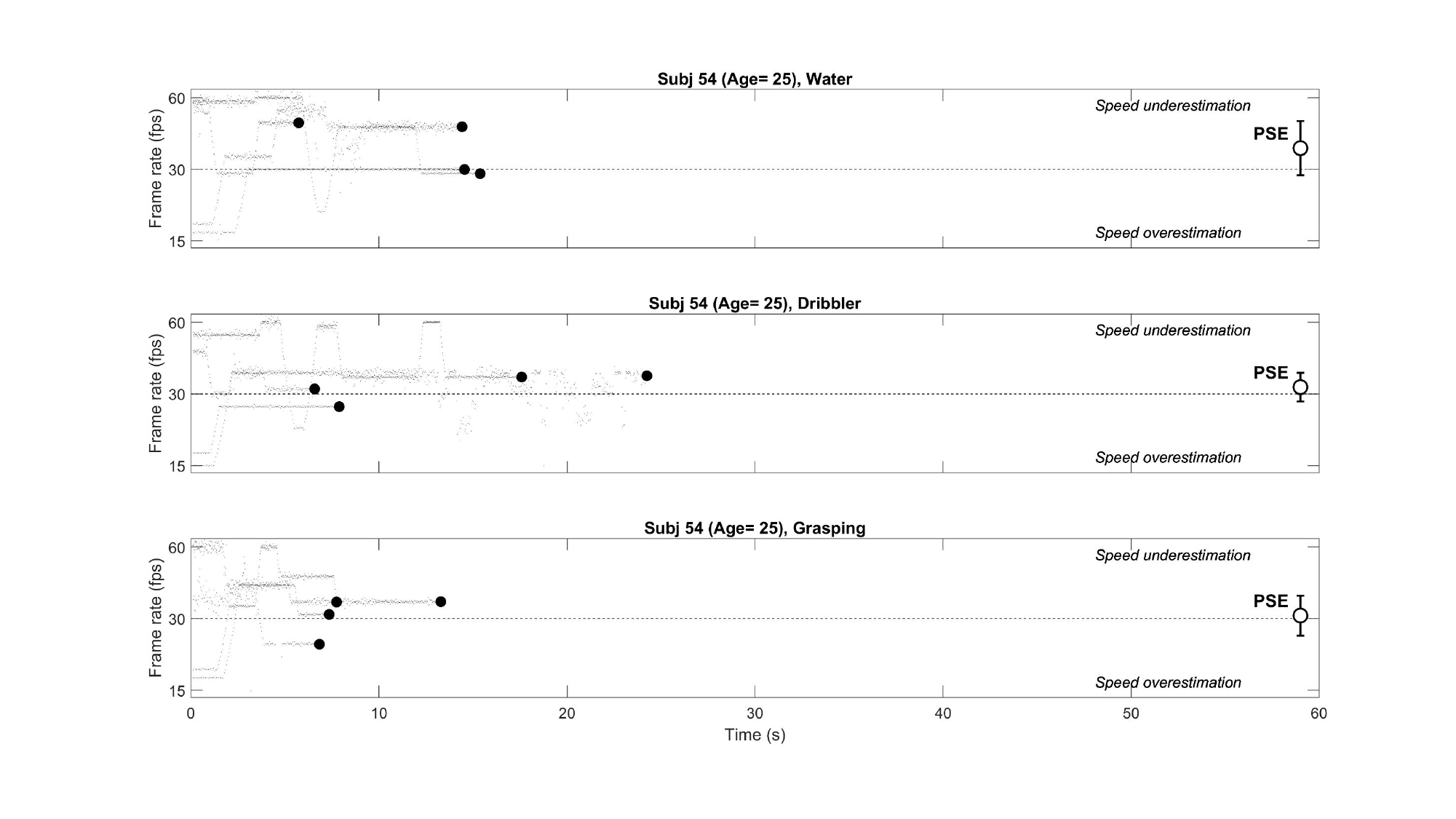

#

## Slide 32
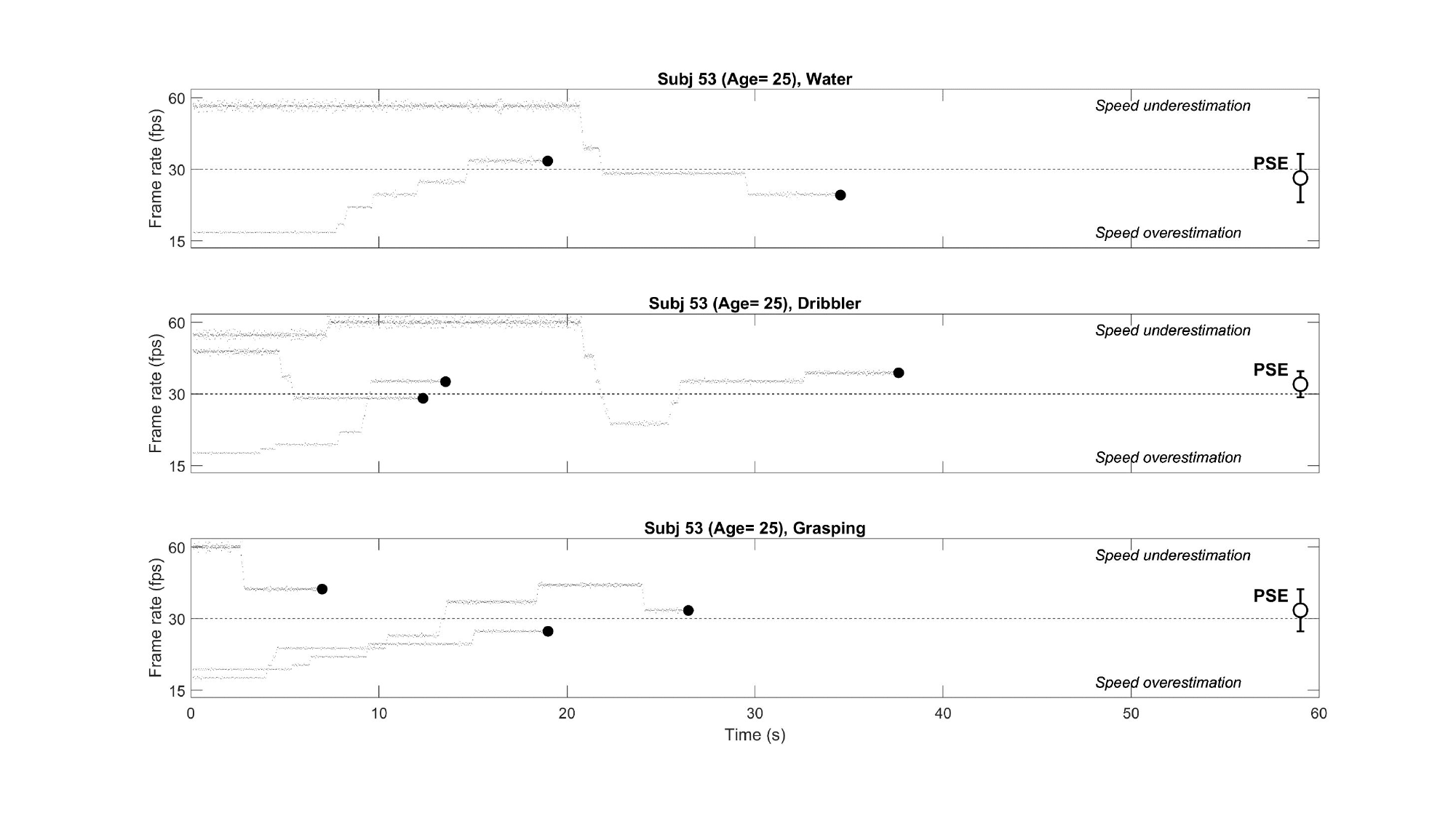

#

## Slide 33
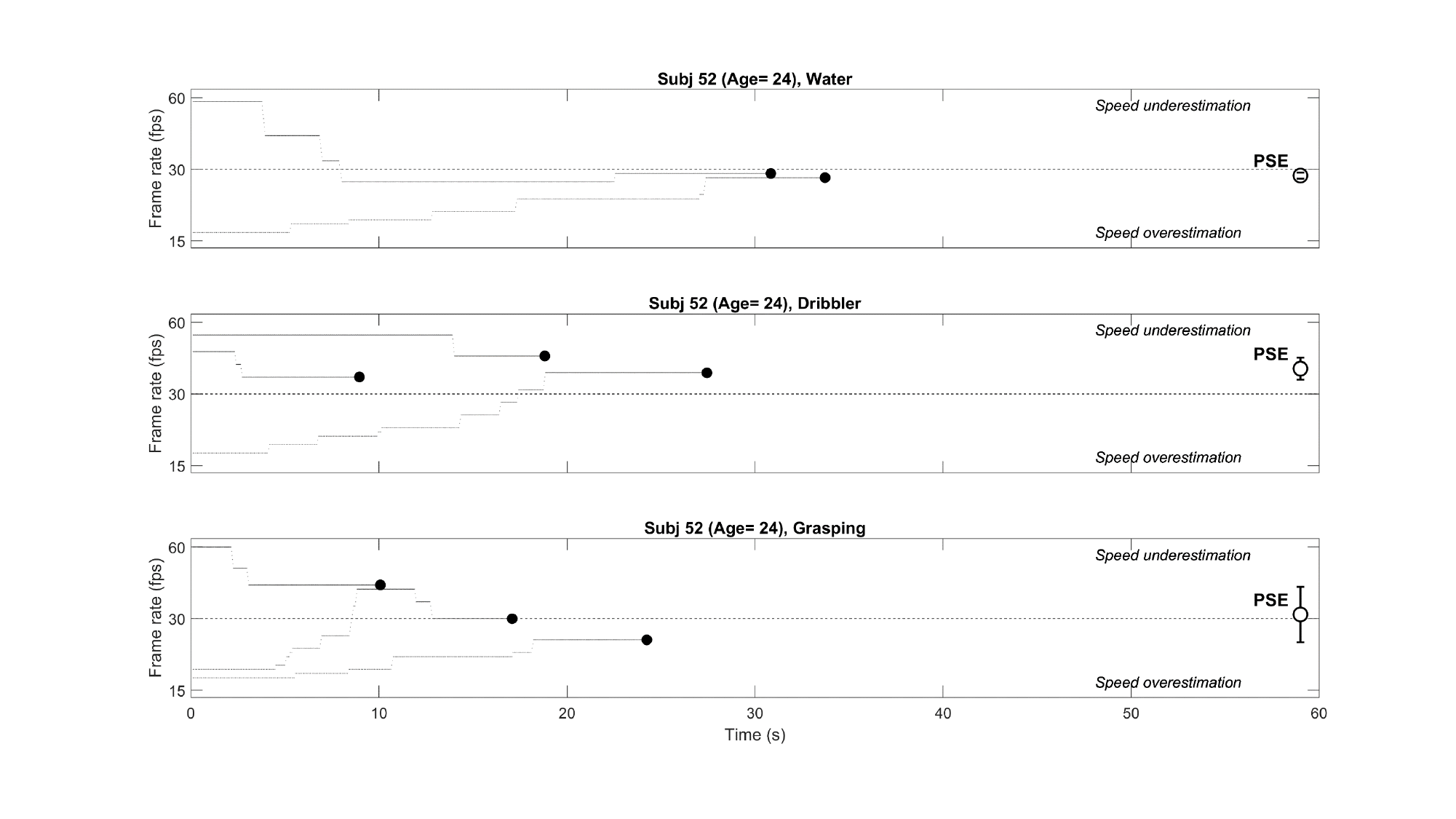

#

## Slide 34
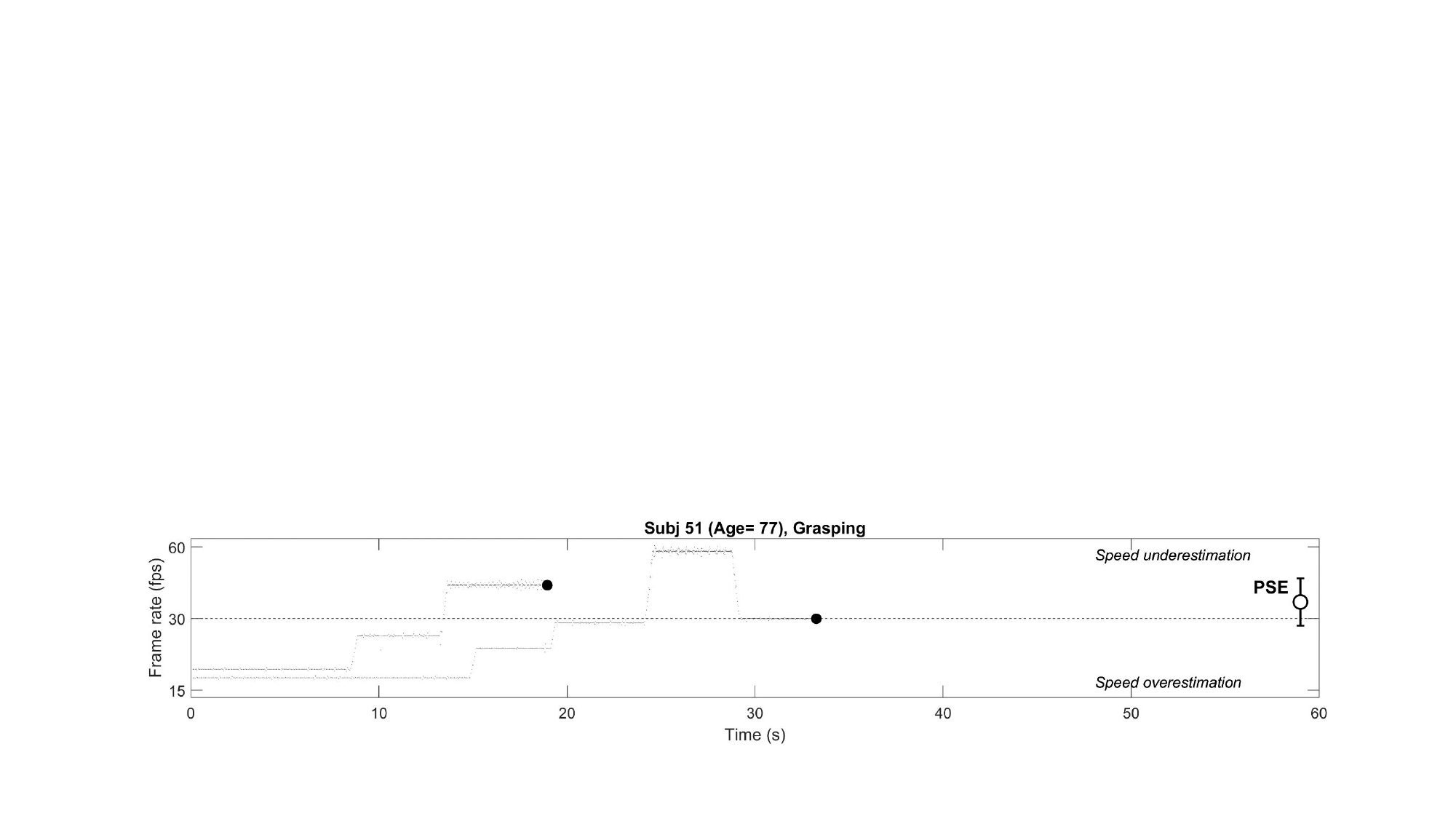

#

## Slide 35
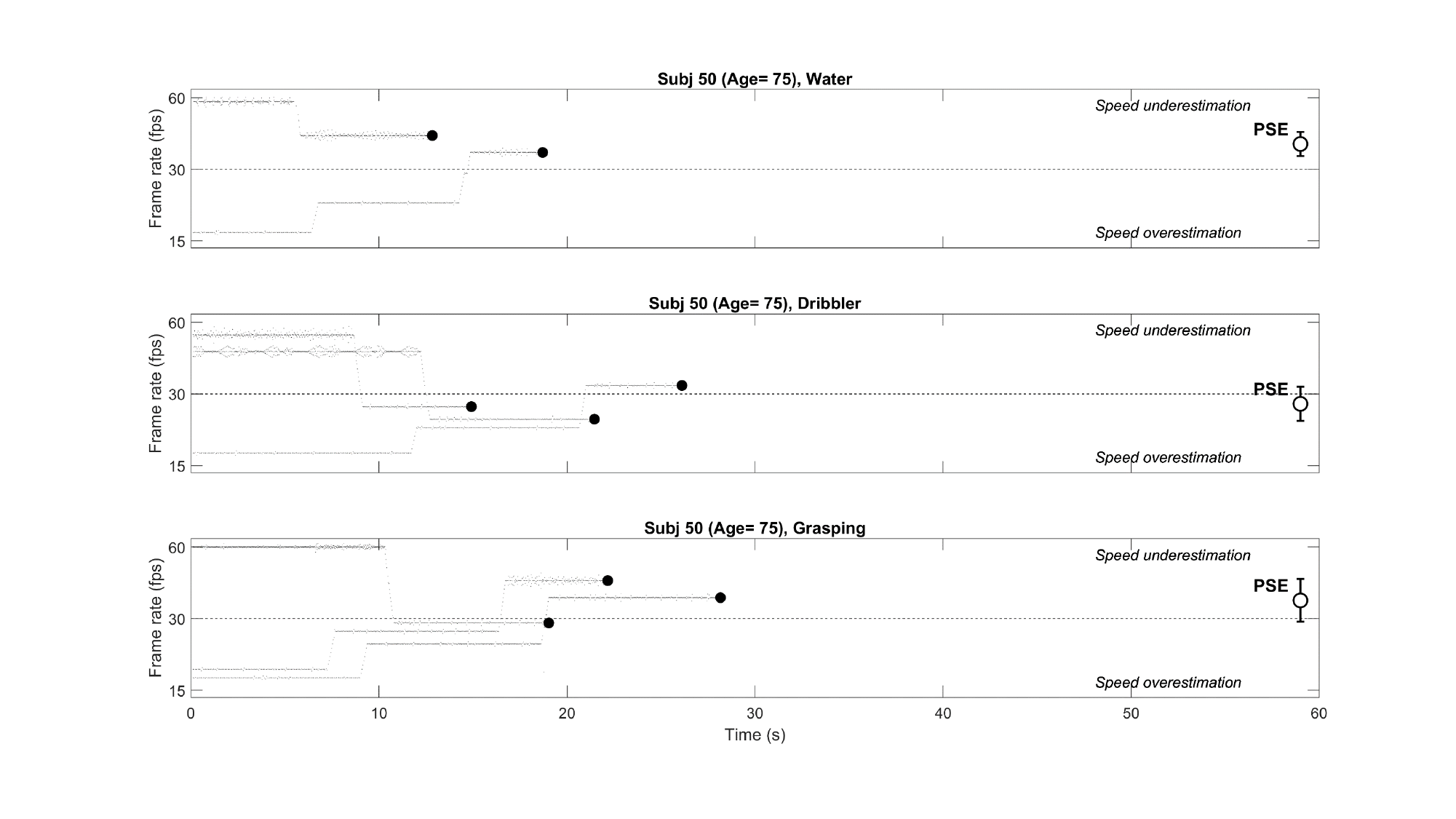

#

## Slide 36
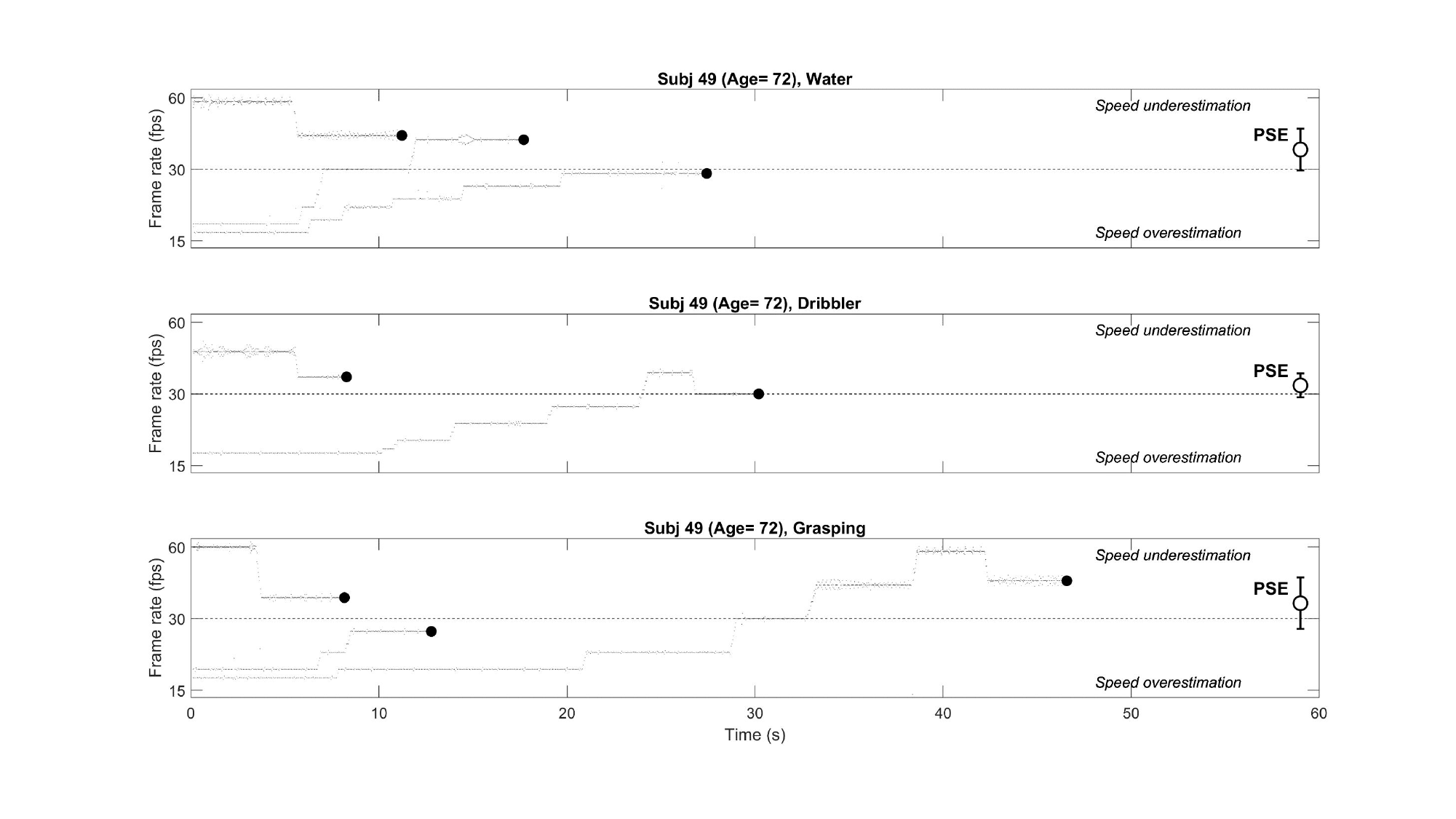

#

## Slide 37
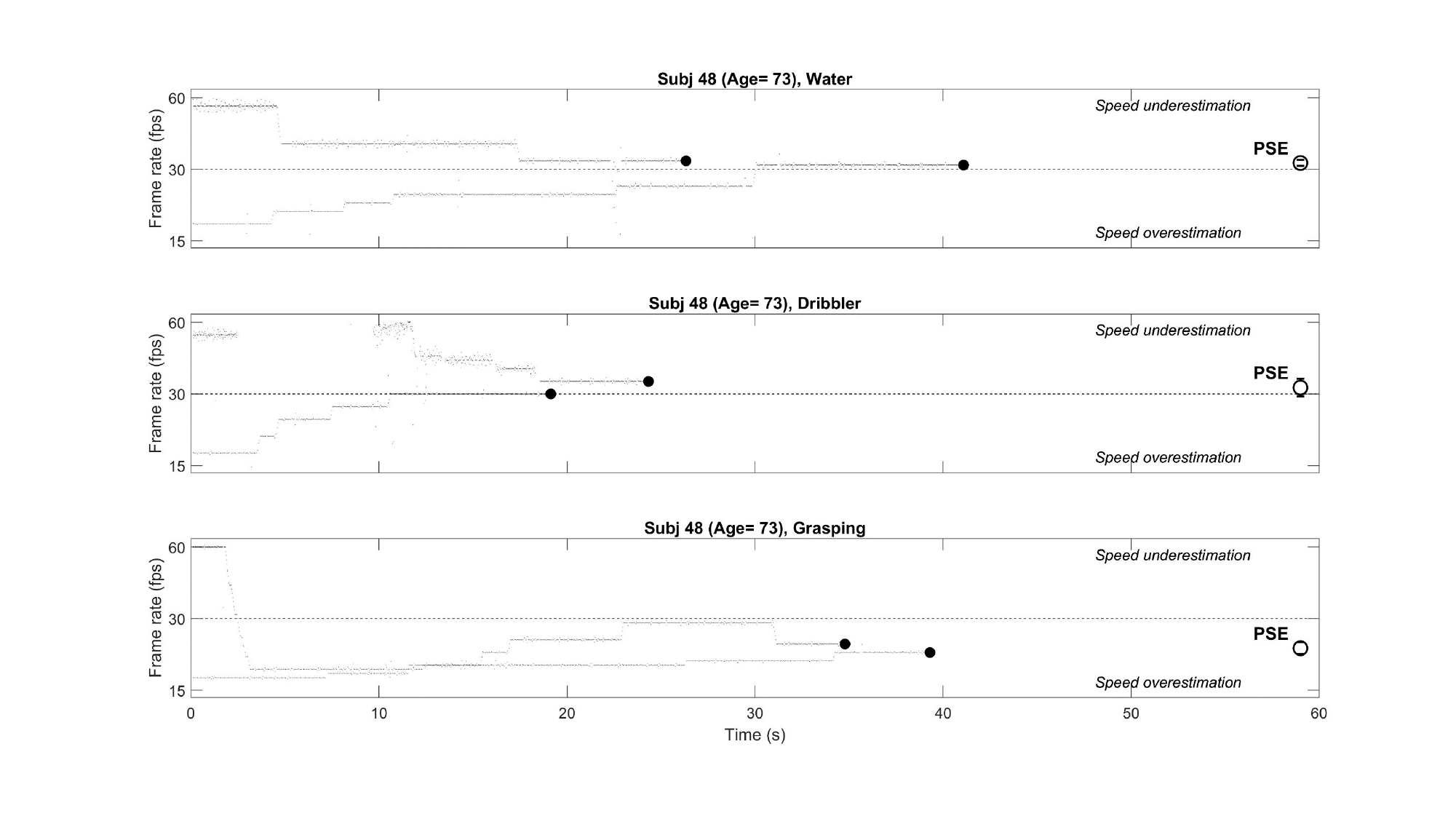

#

## Slide 38
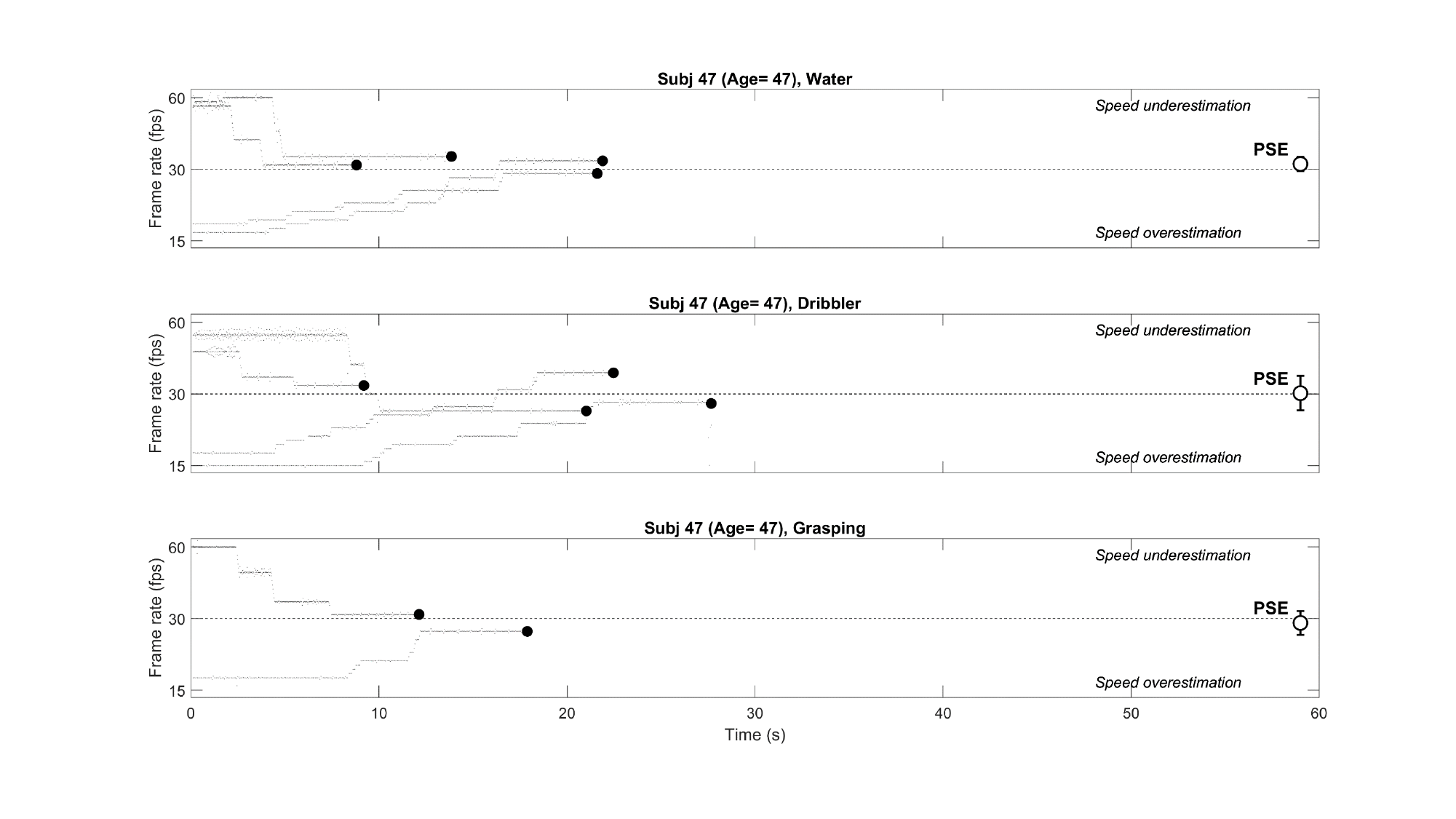

#

## Slide 39
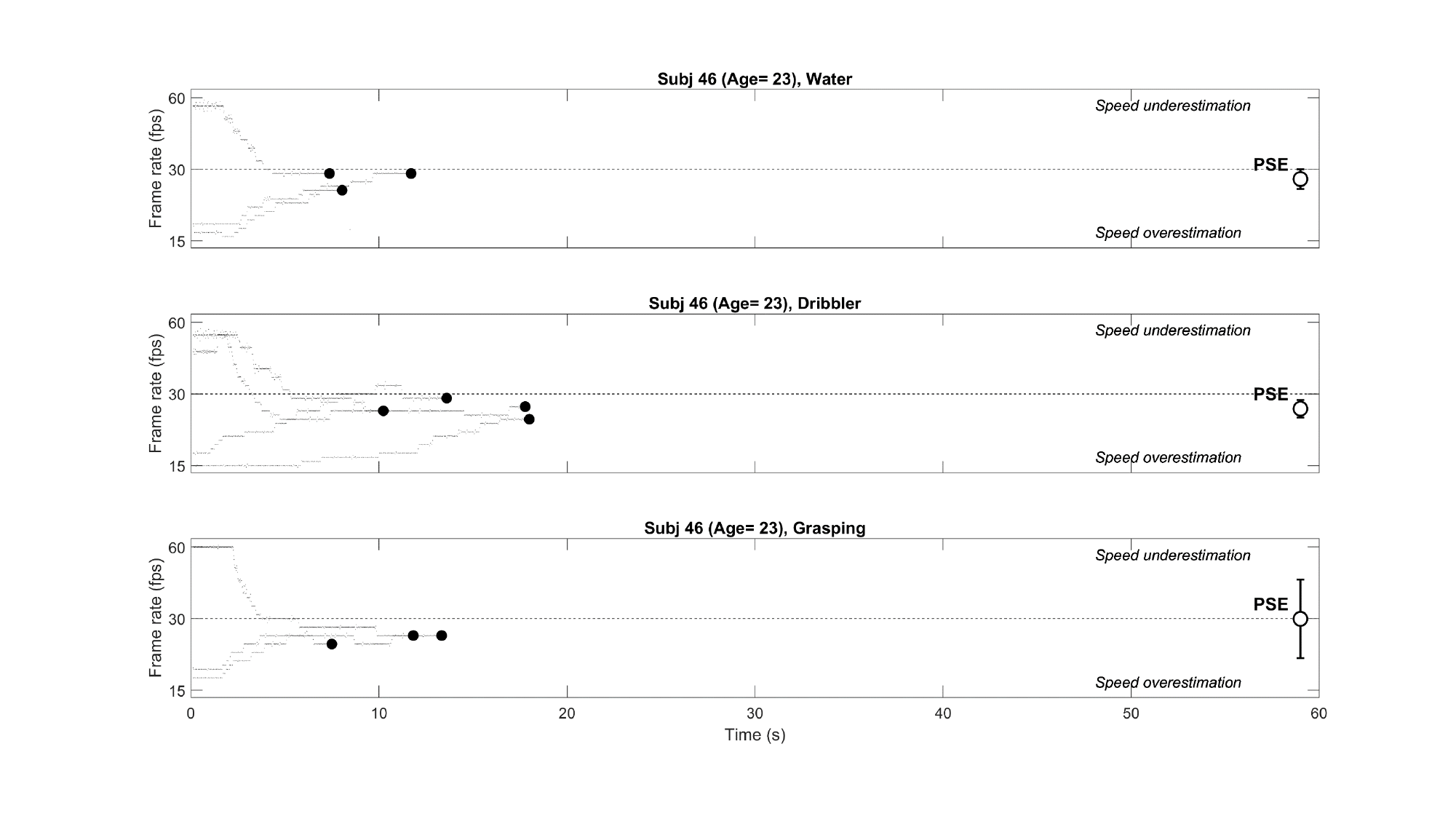

#

## Slide 40
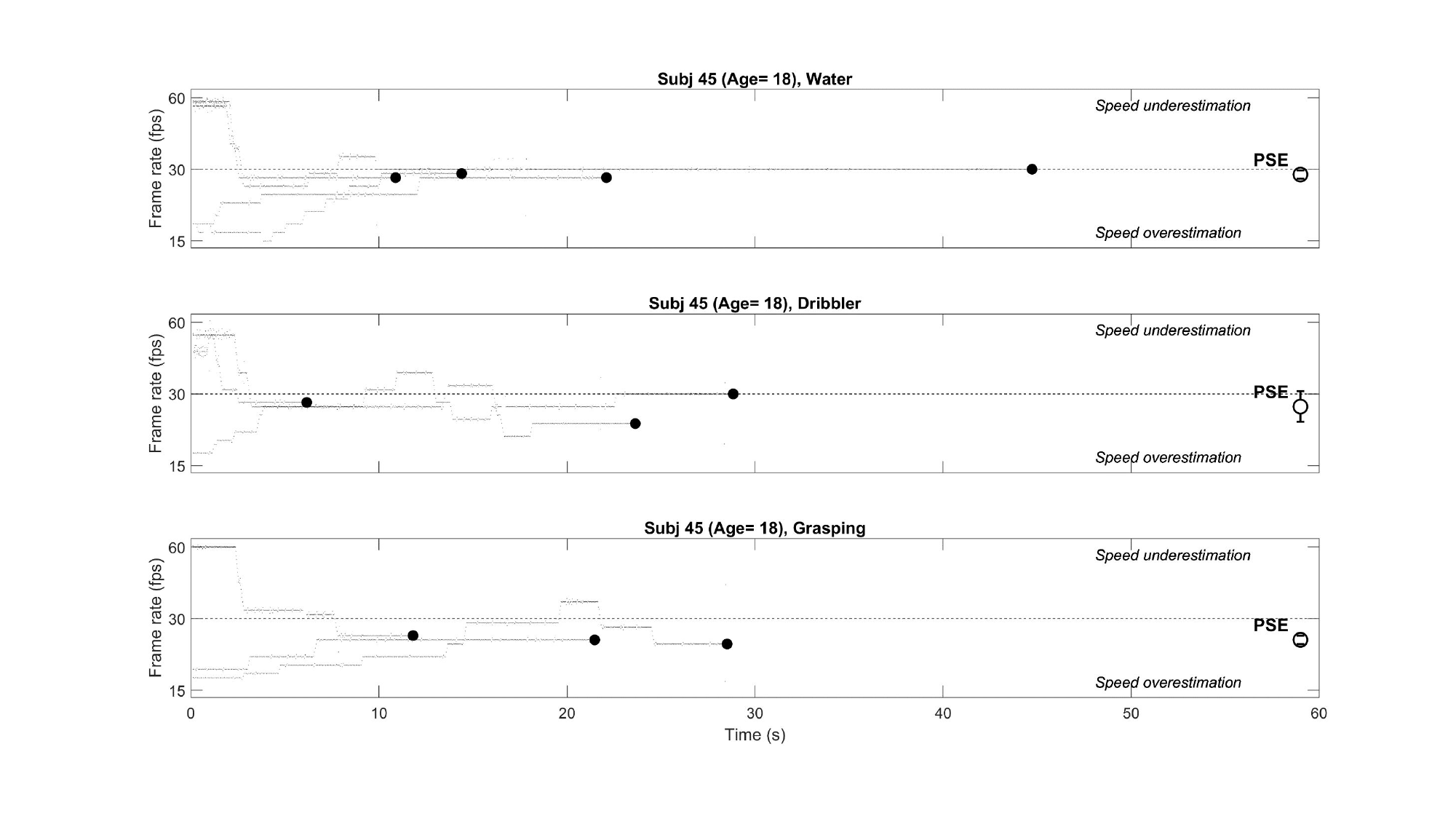

#

## Slide 41
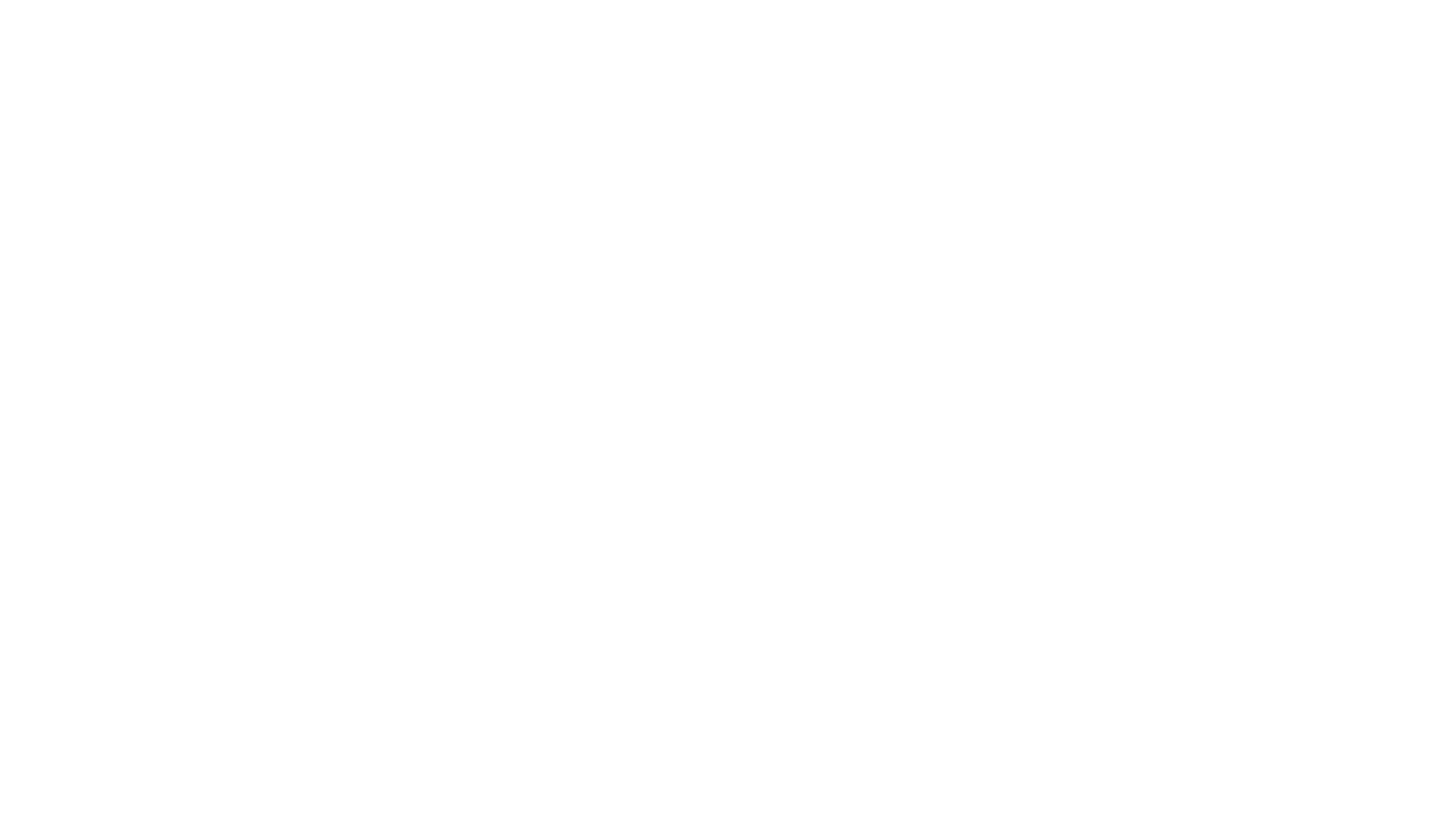

#

## Slide 42
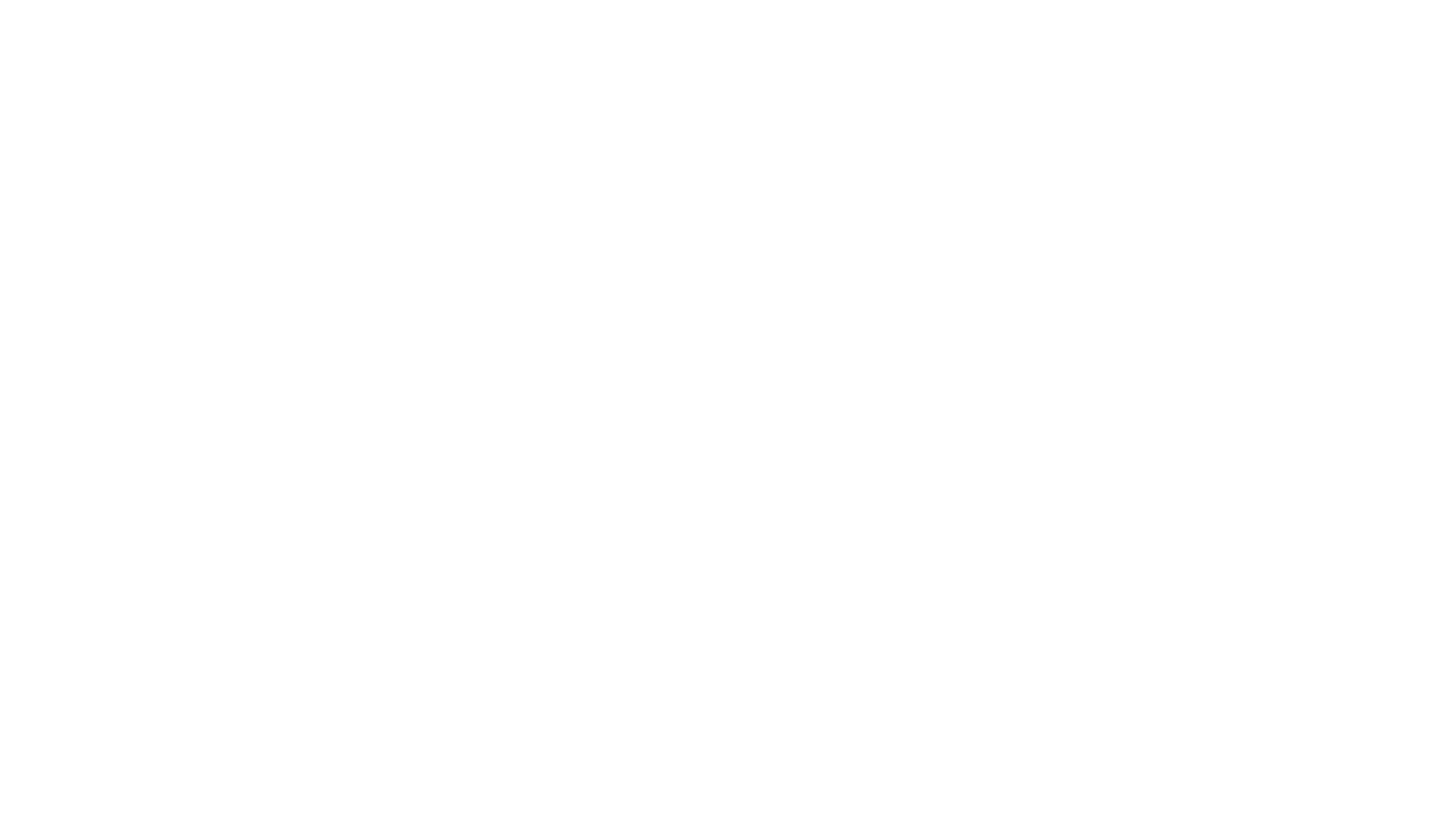

#

## Slide 43
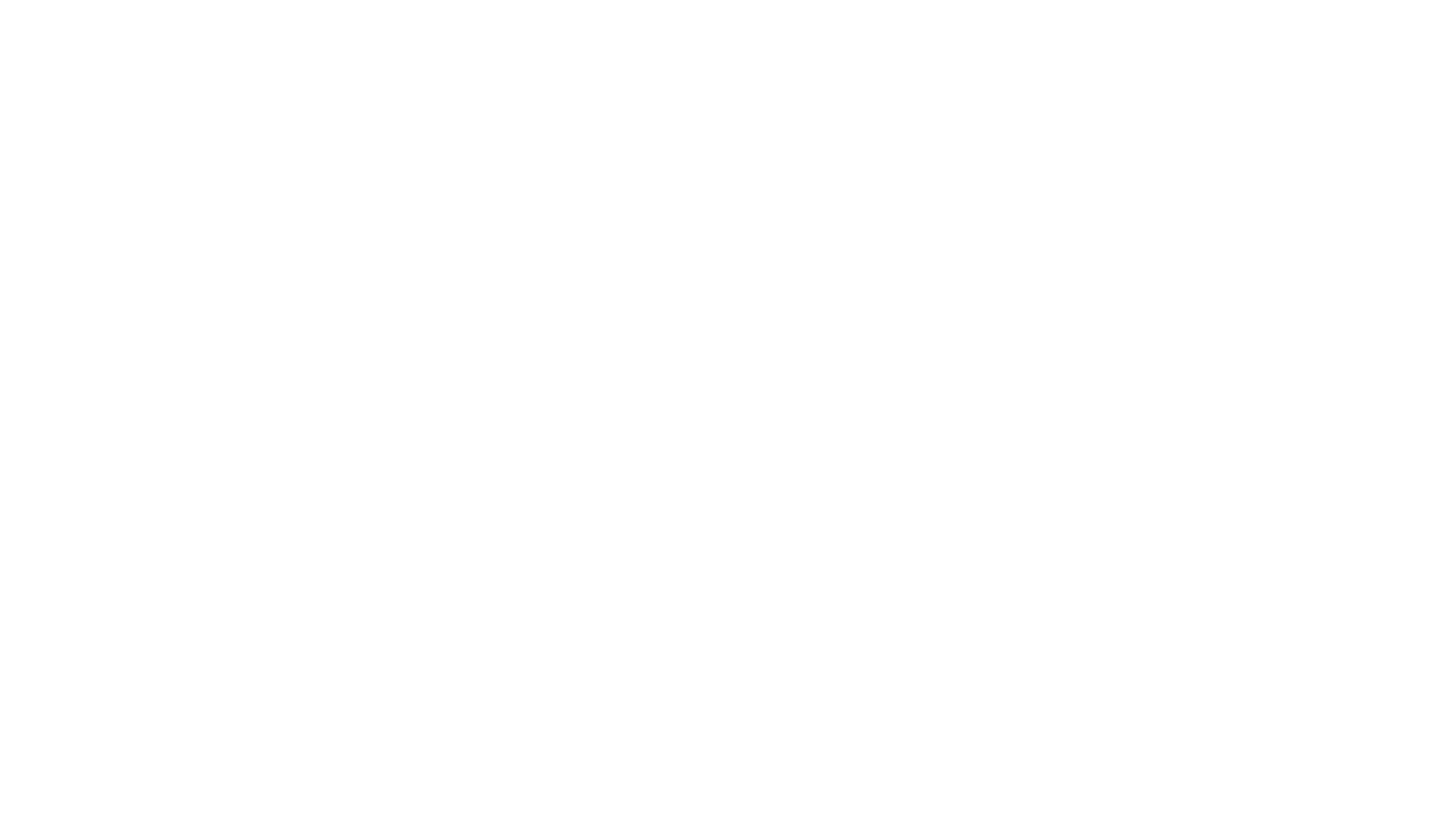

#

## Slide 44
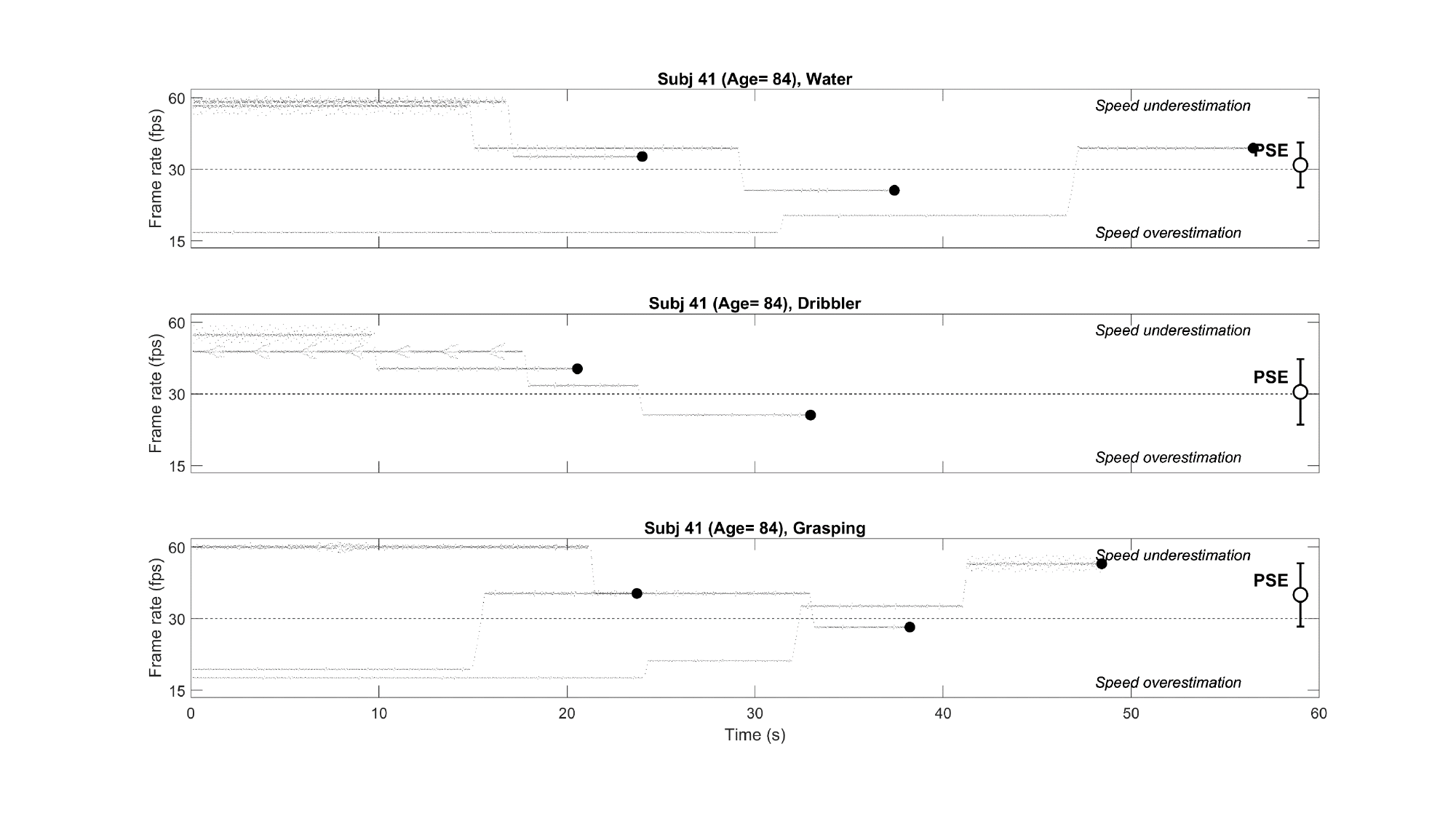

#

## Slide 45
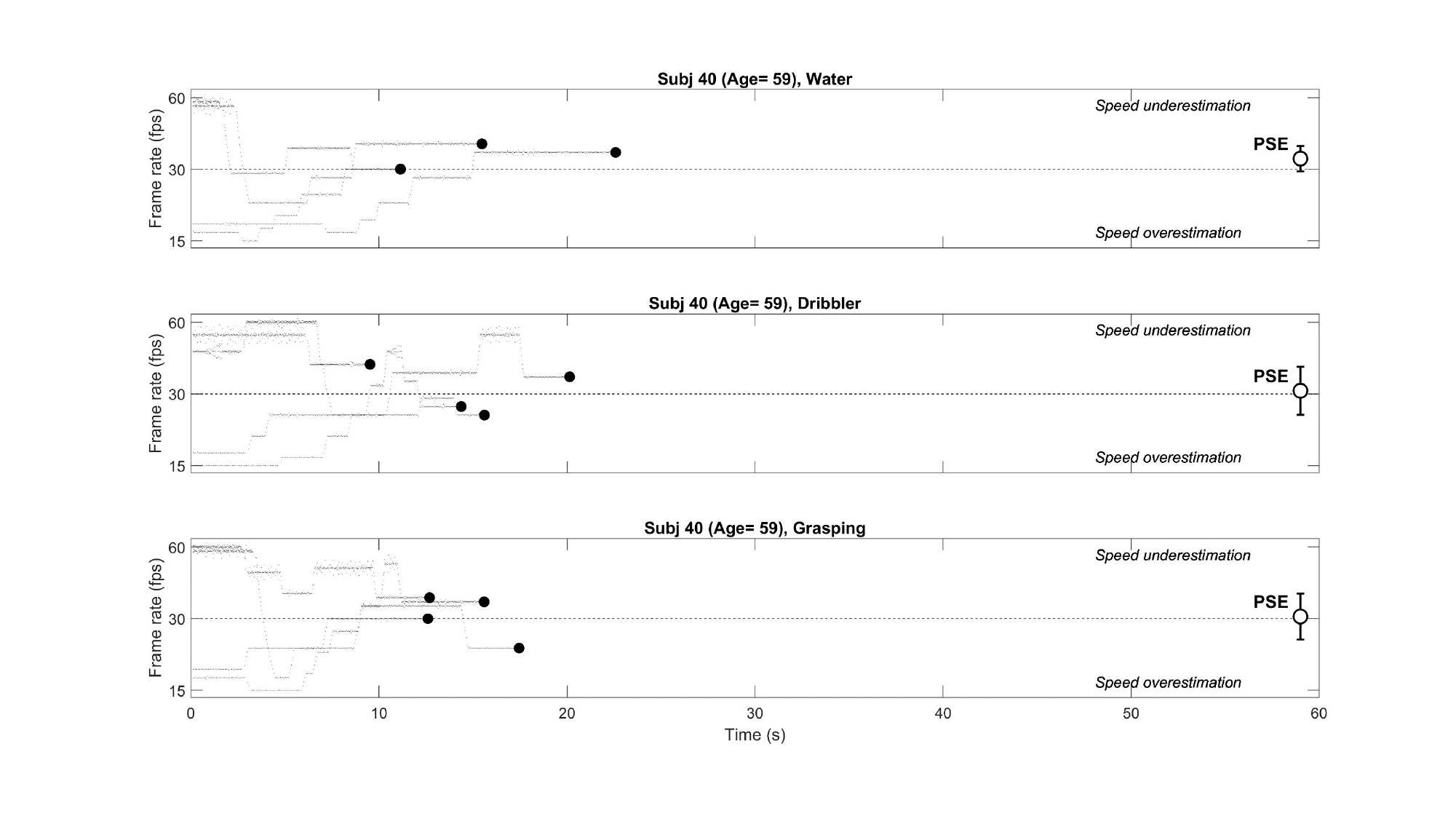

#

## Slide 46
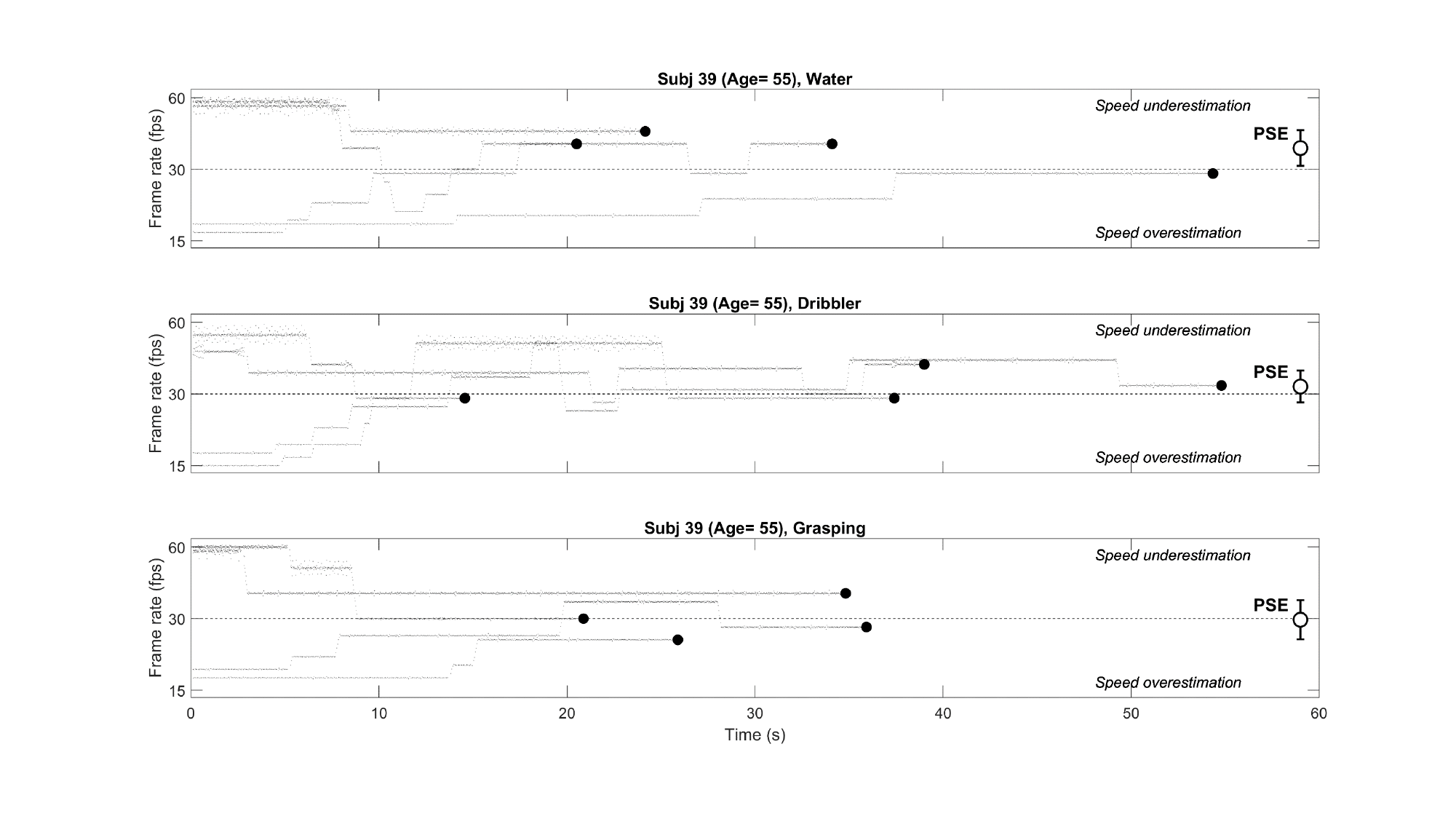

#

## Slide 47
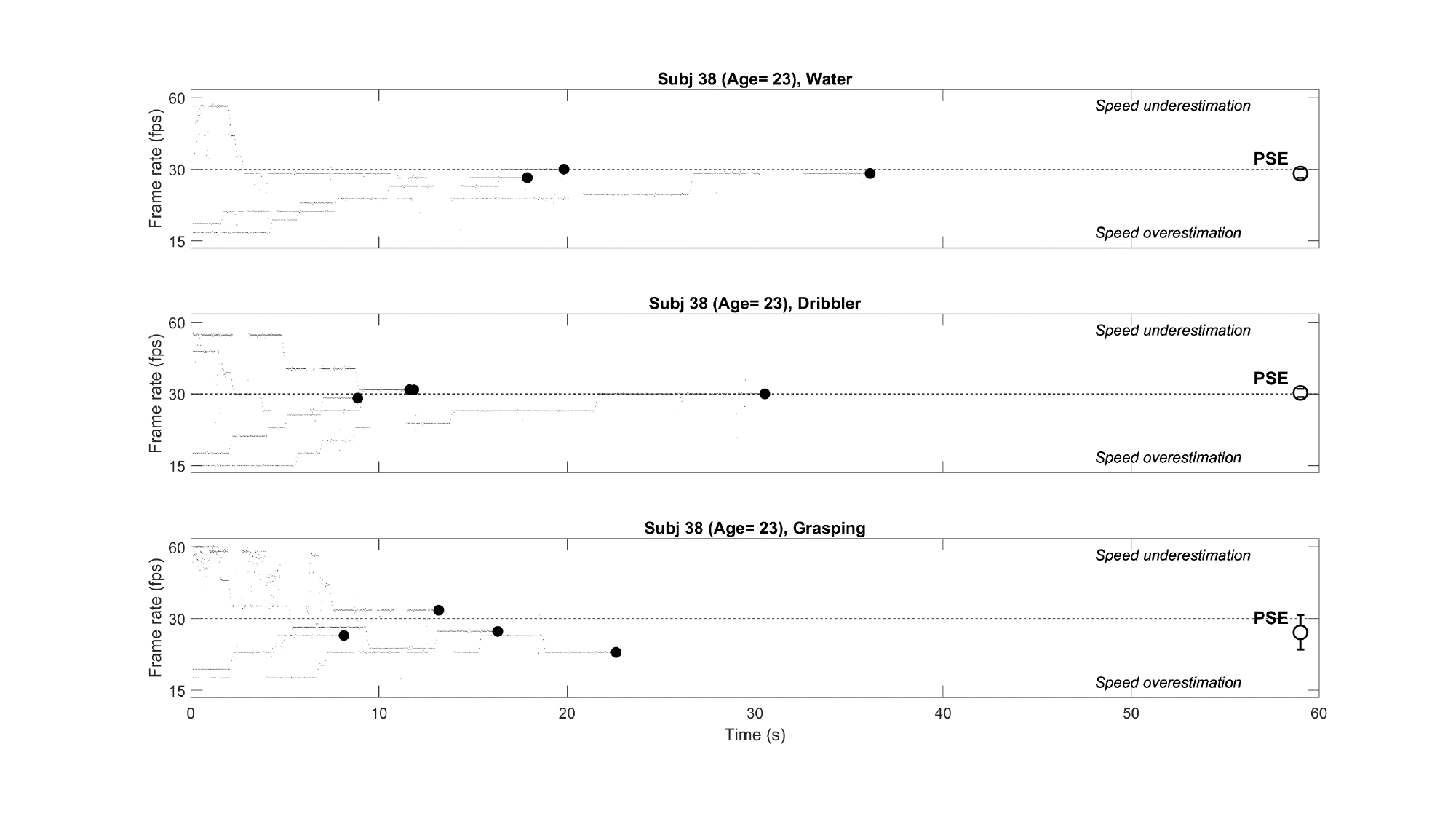

#

## Slide 48
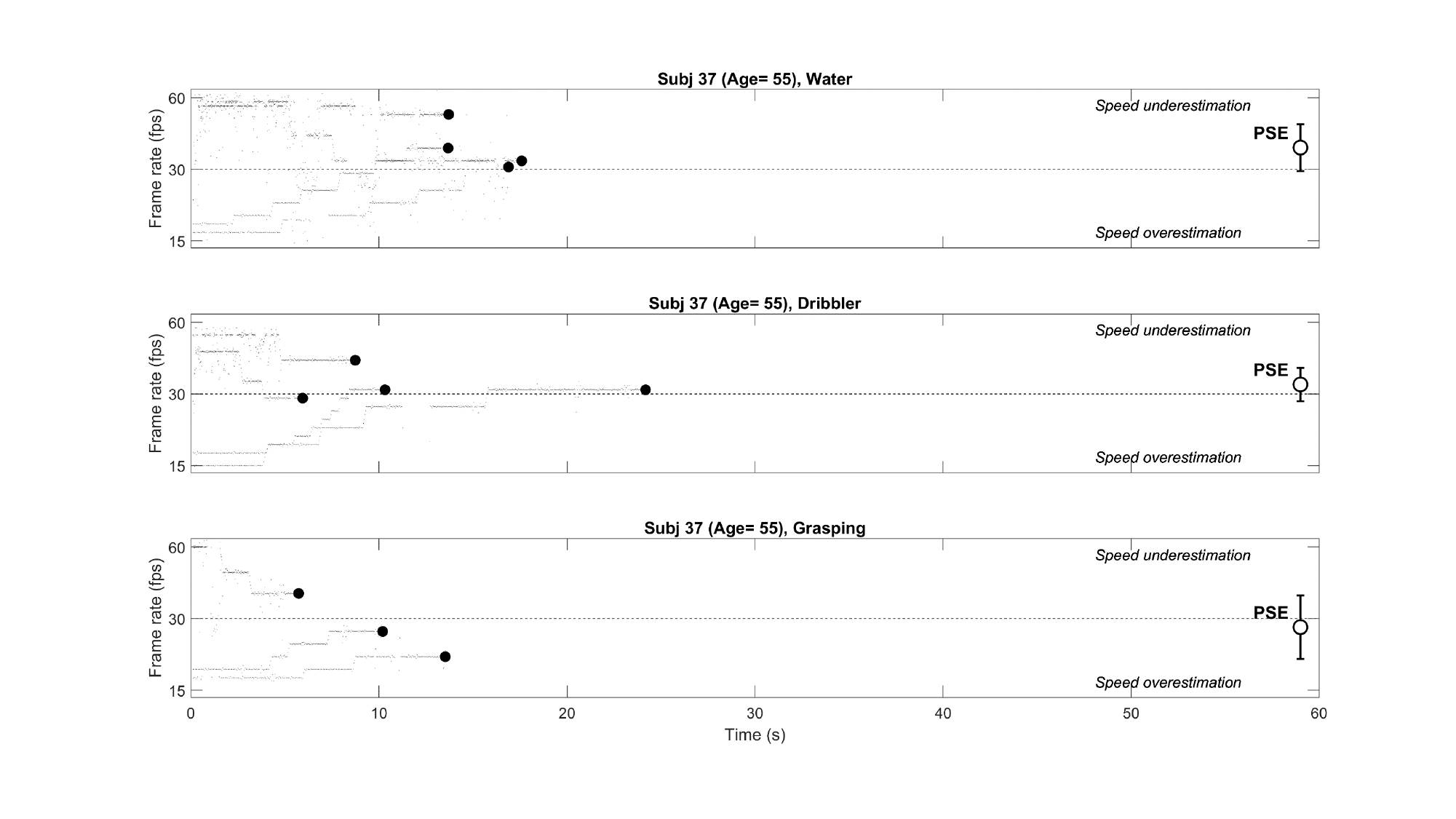

#

## Slide 49
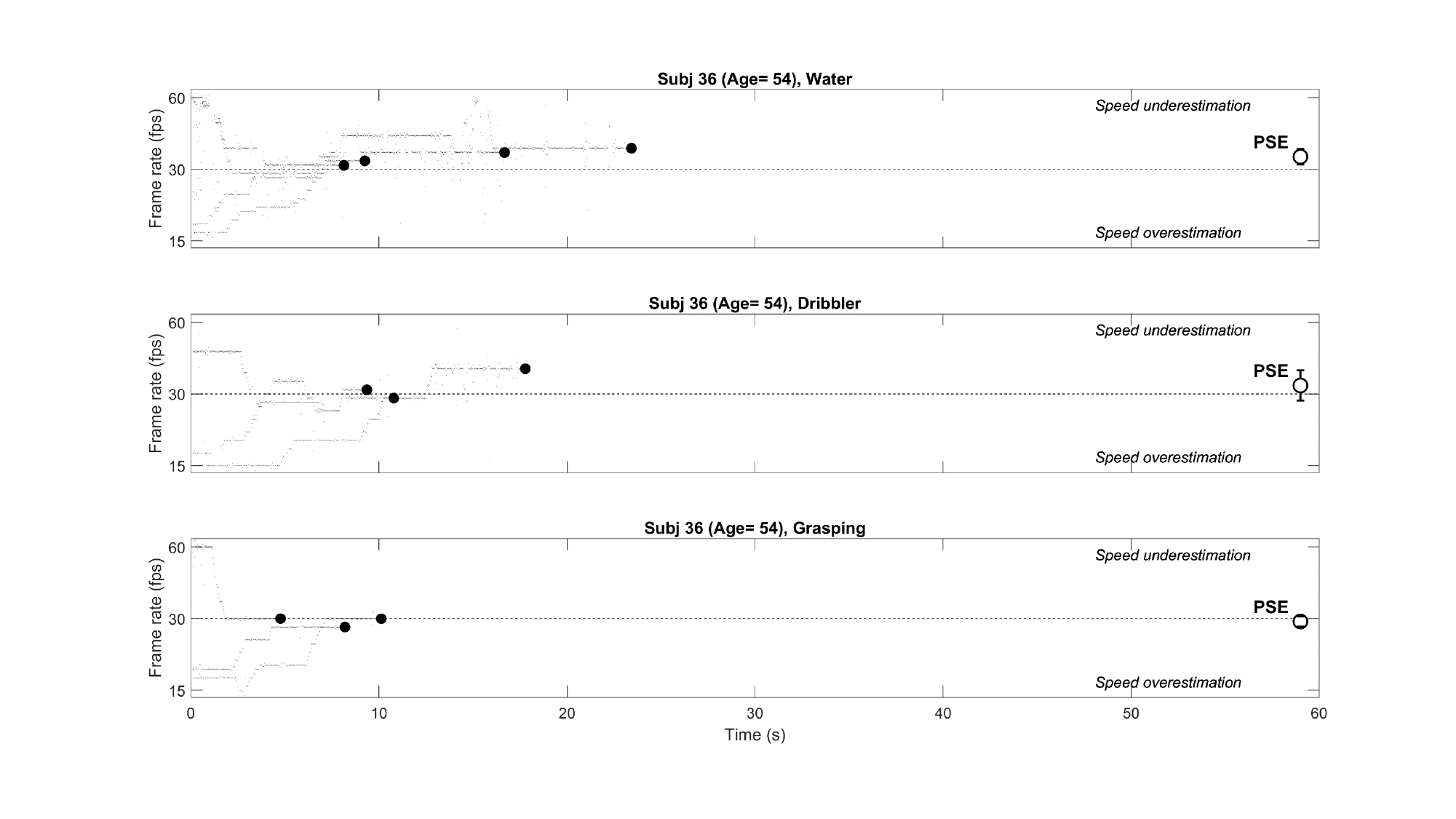

#

## Slide 50
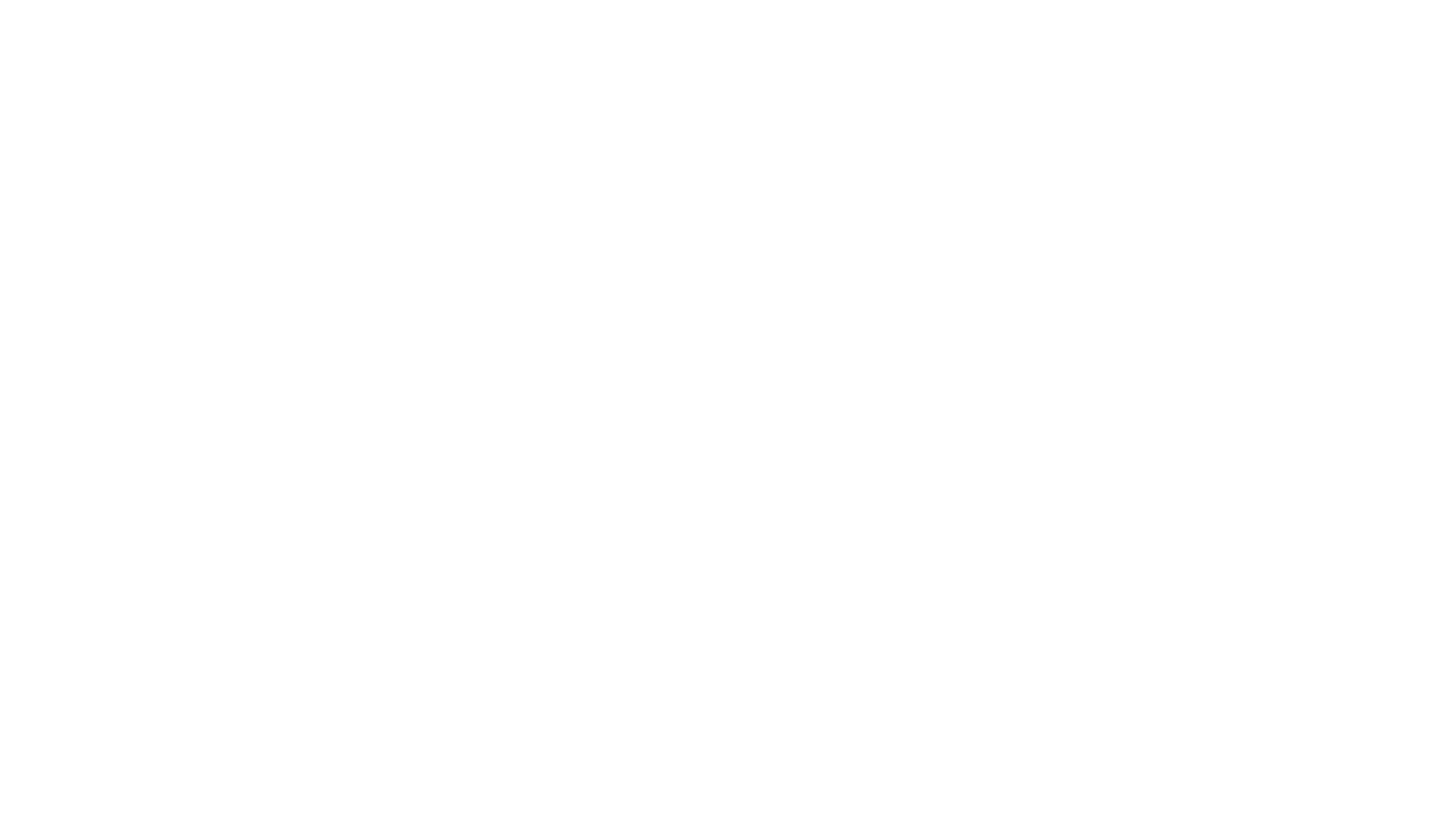

#

## Slide 51
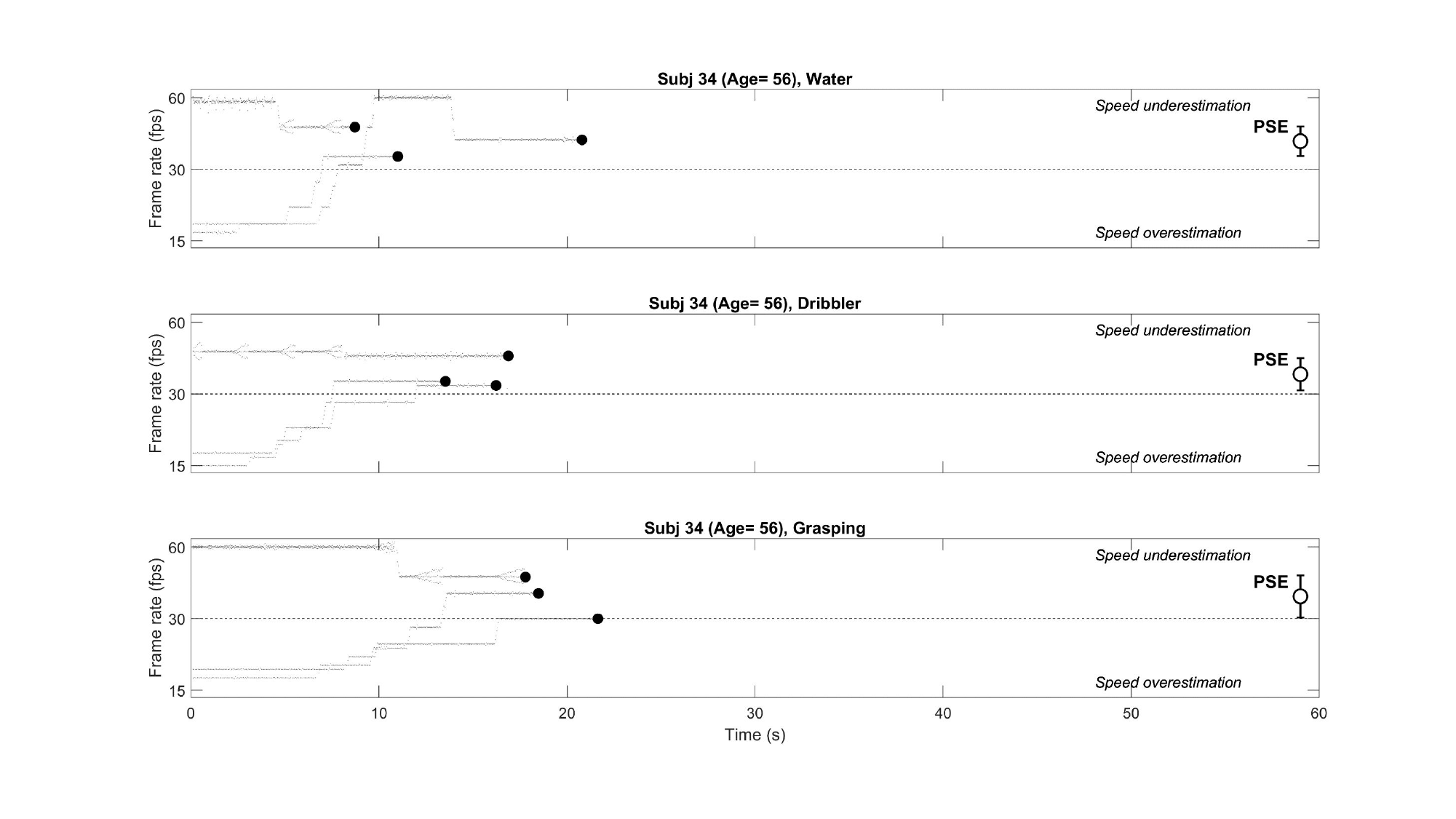

#

## Slide 52
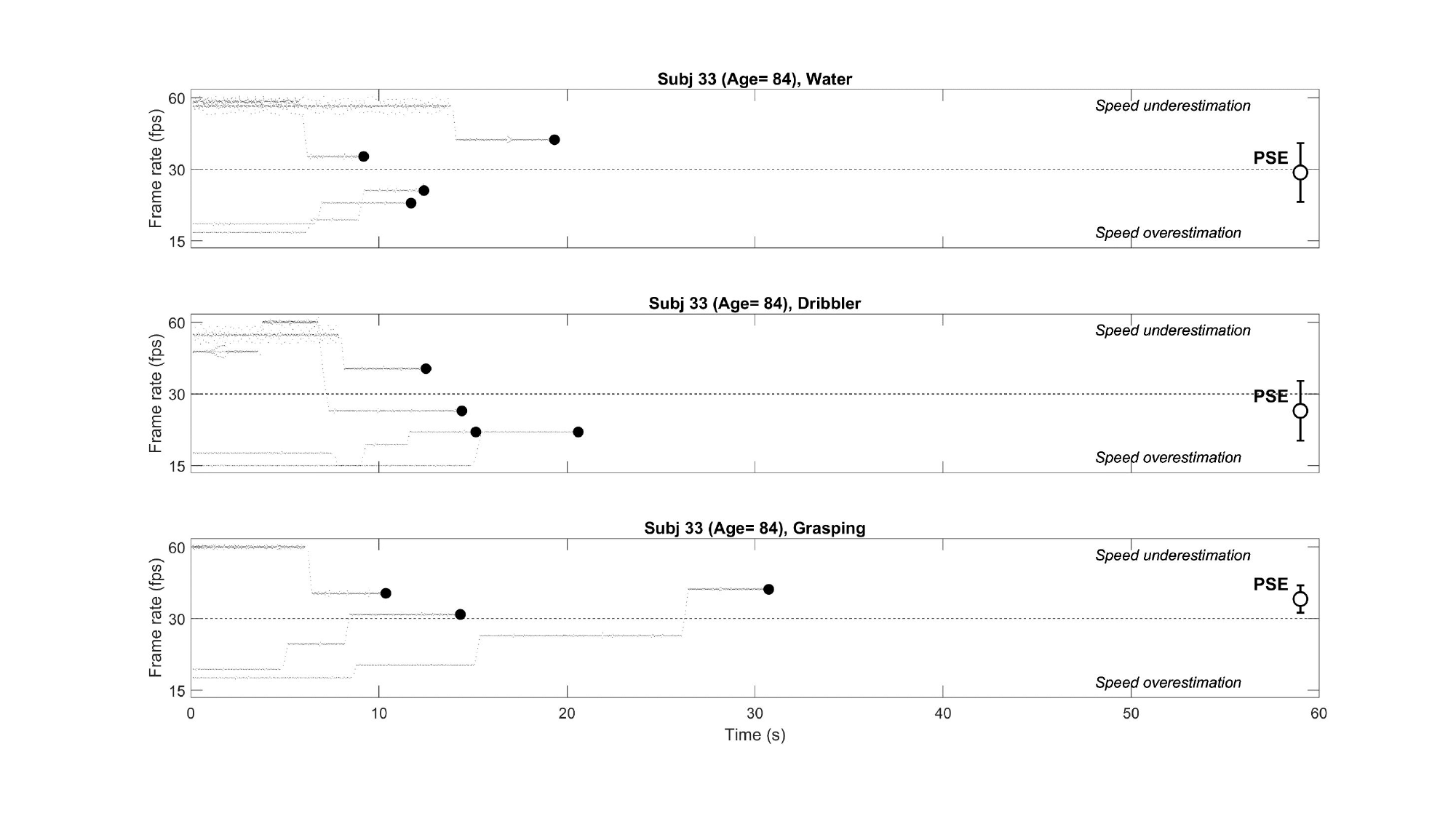

#

## Slide 53
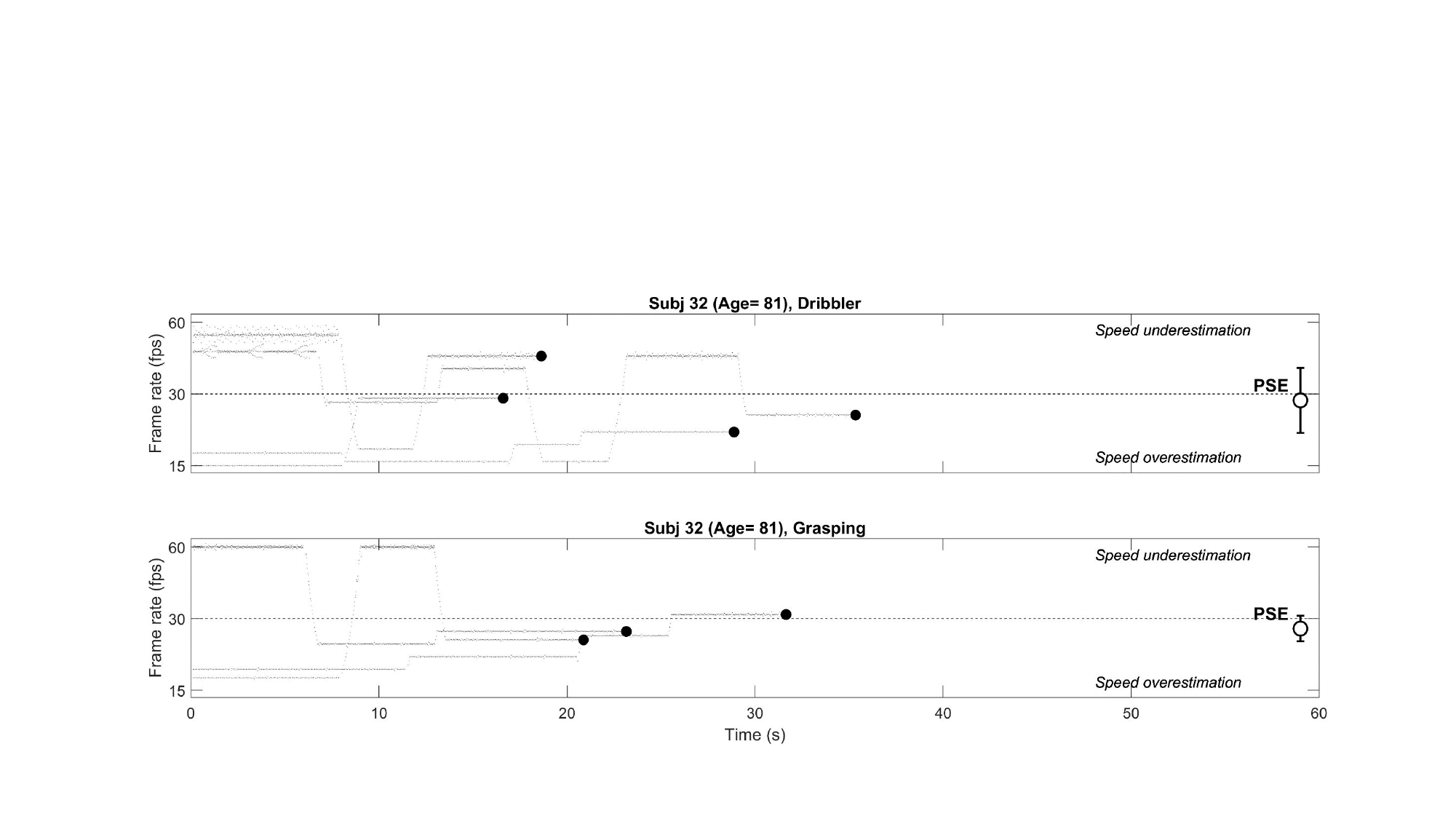

#

## Slide 54
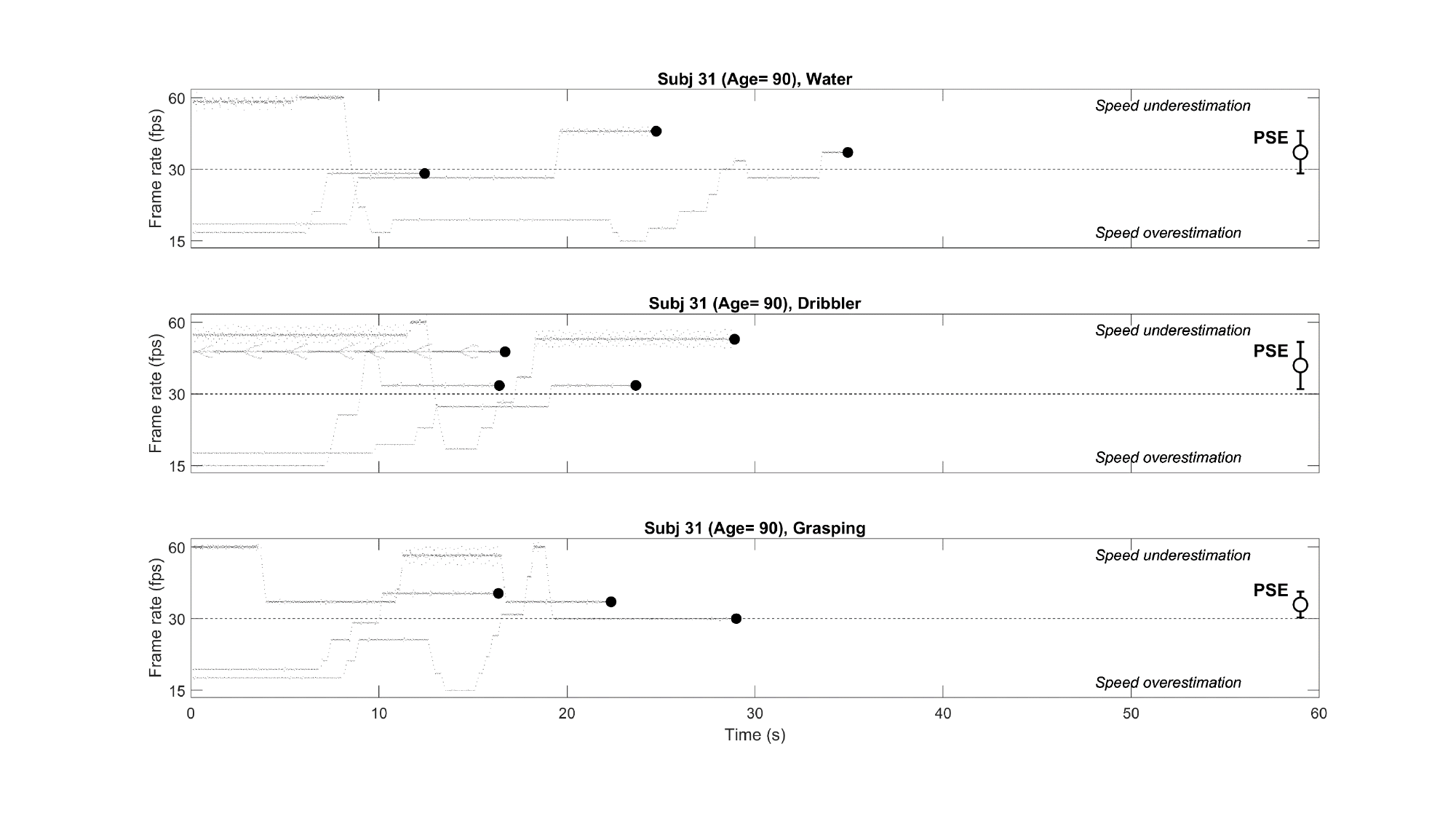

#

## Slide 55
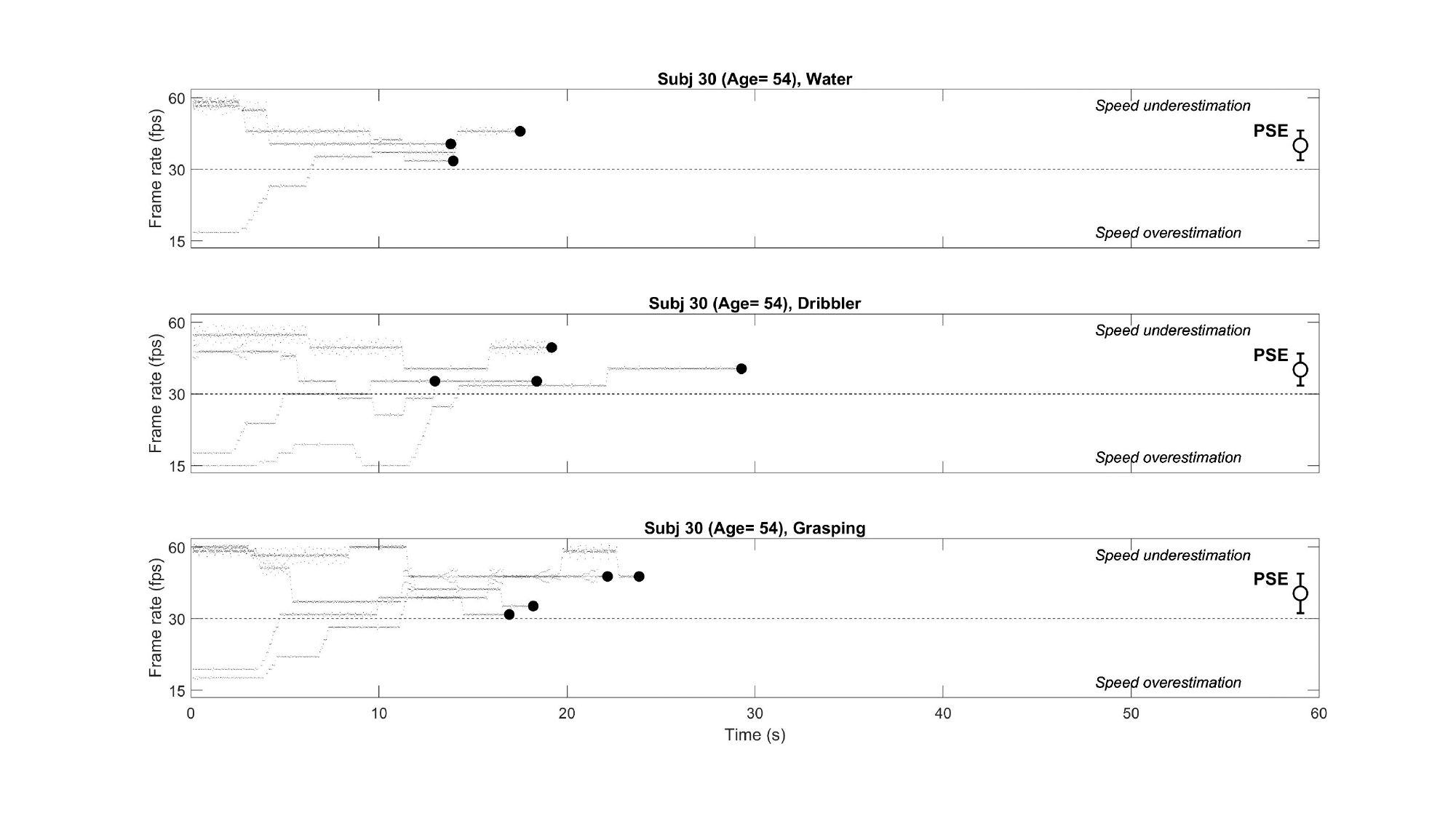

#

## Slide 56
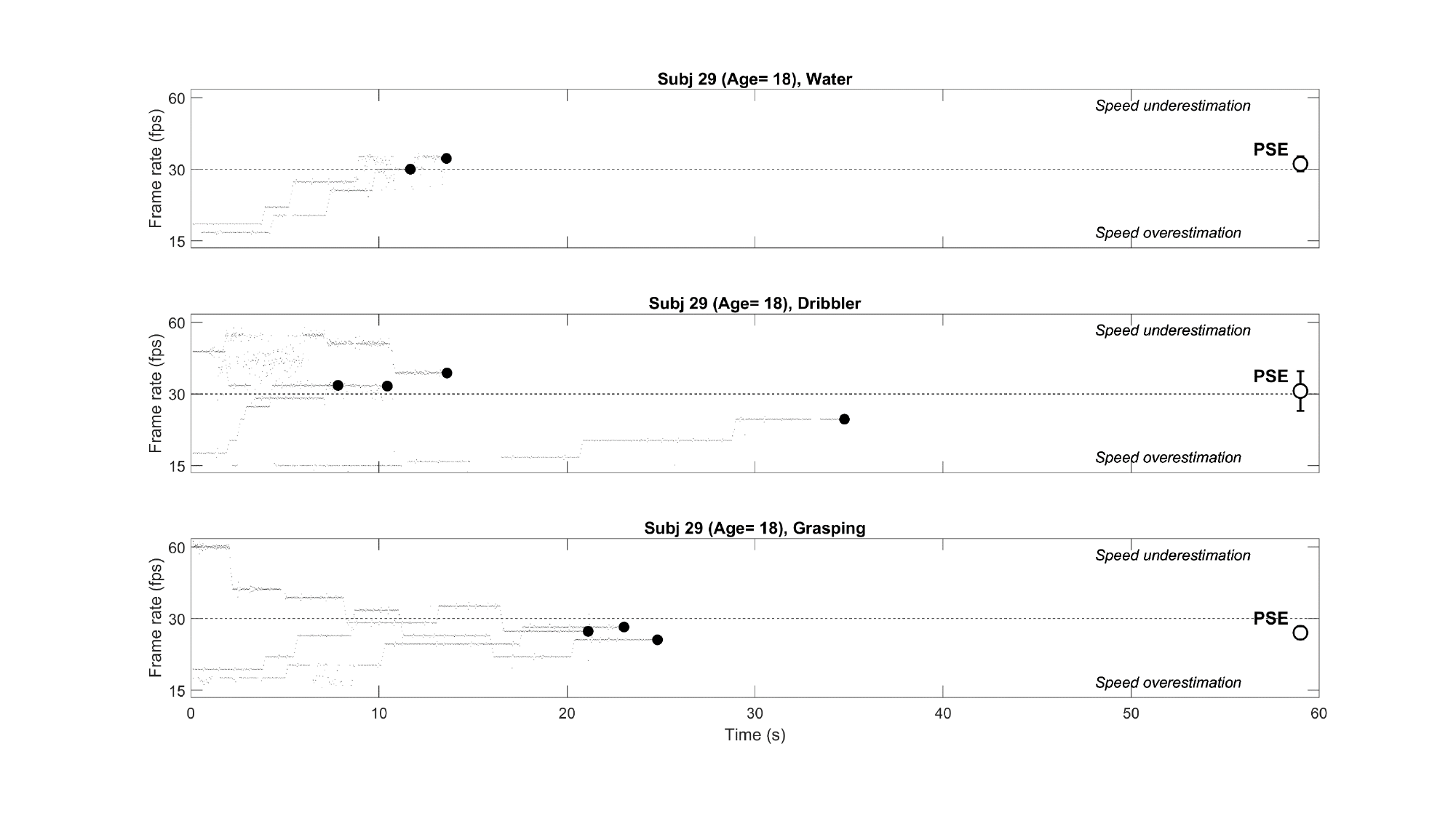

#

## Slide 57
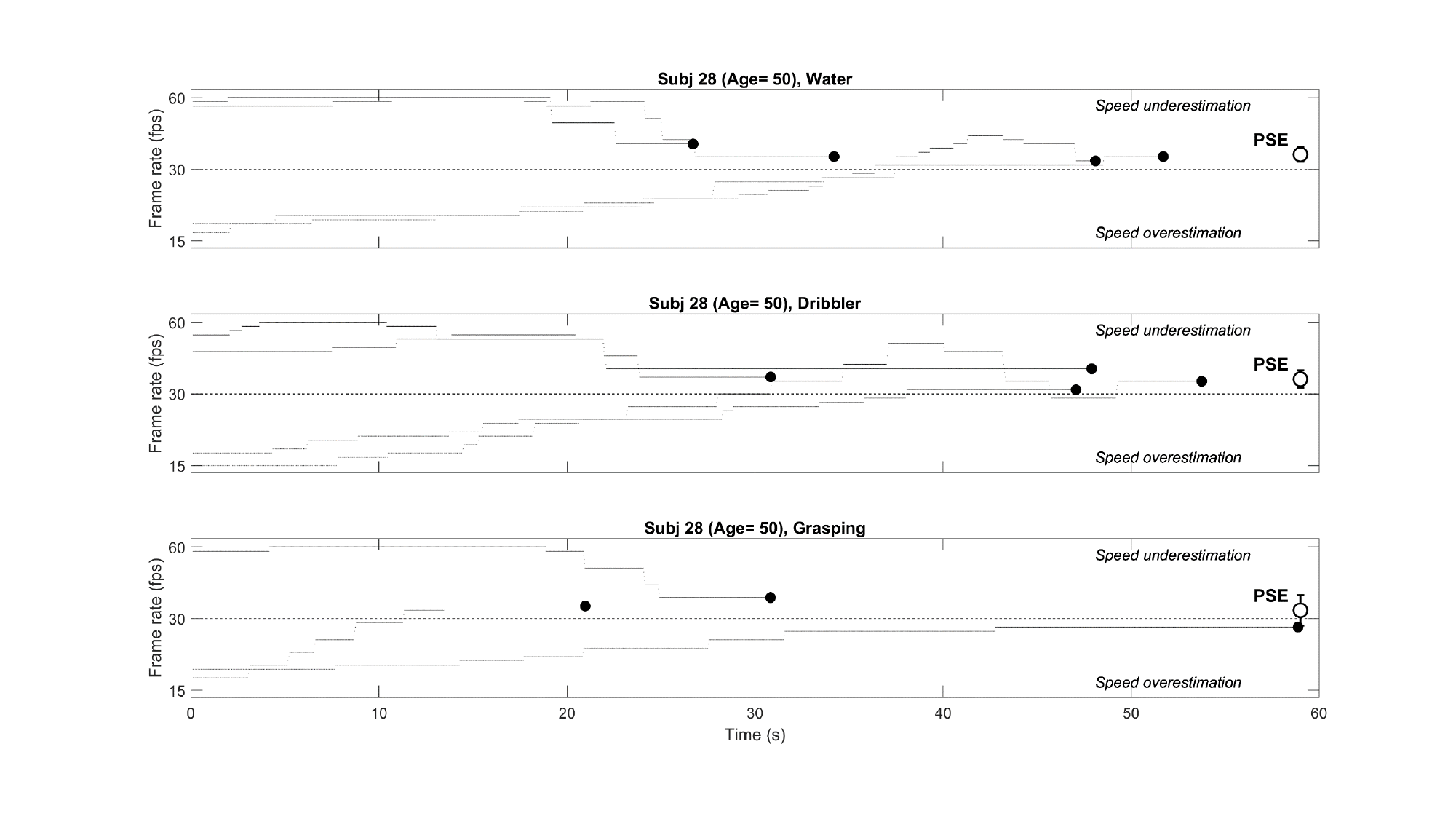

#

## Slide 58
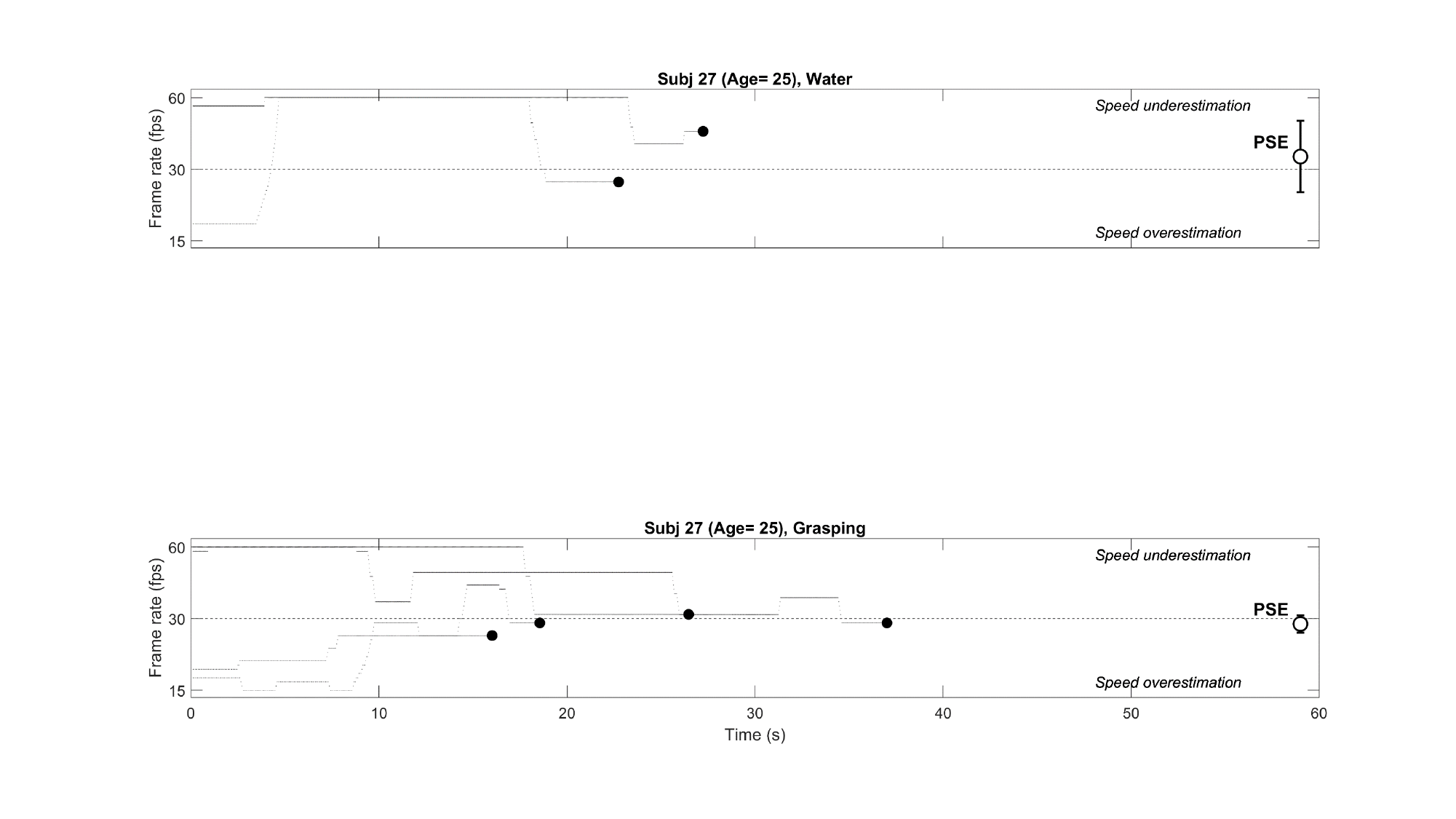

#

## Slide 59
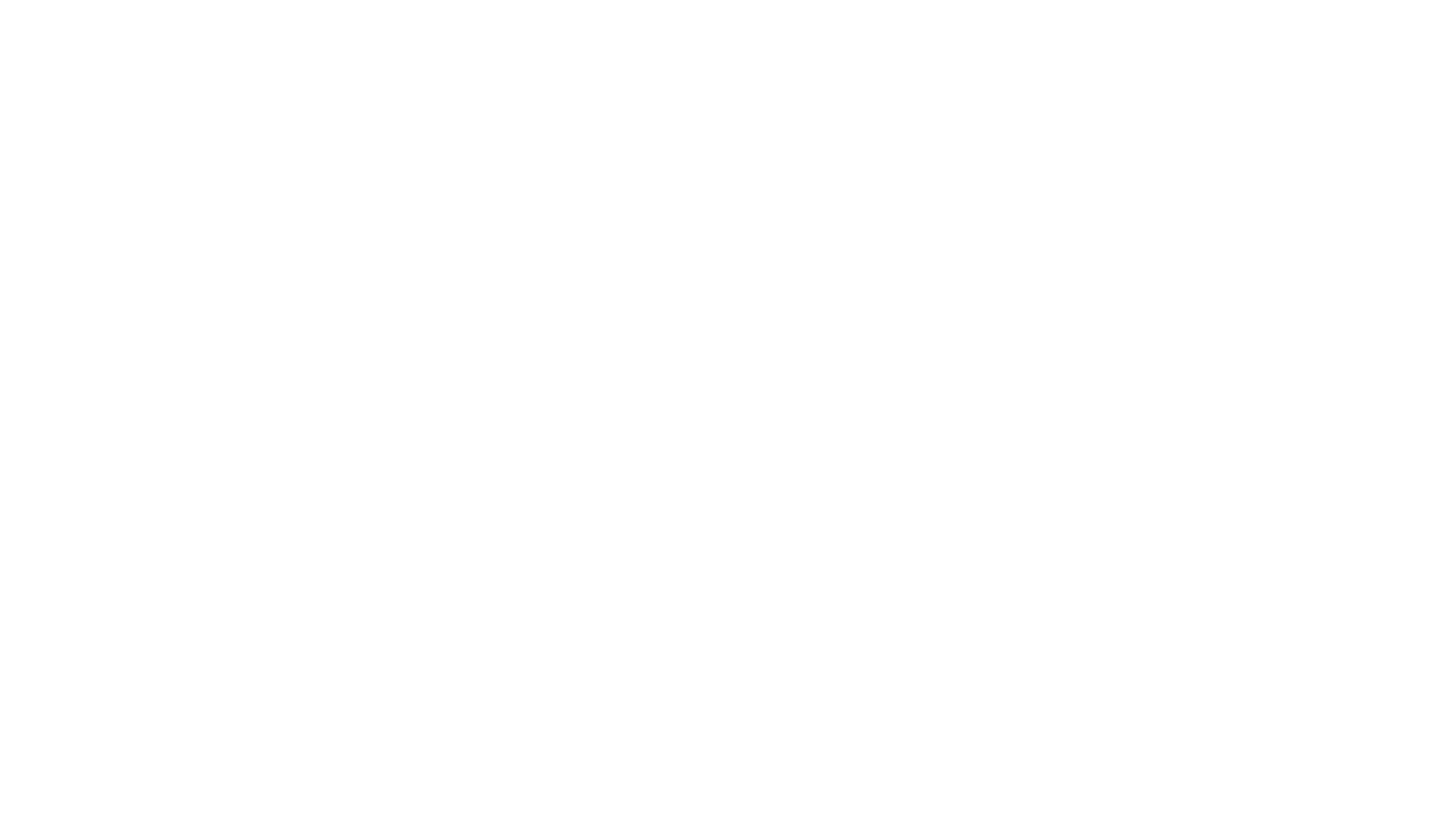

#

## Slide 60
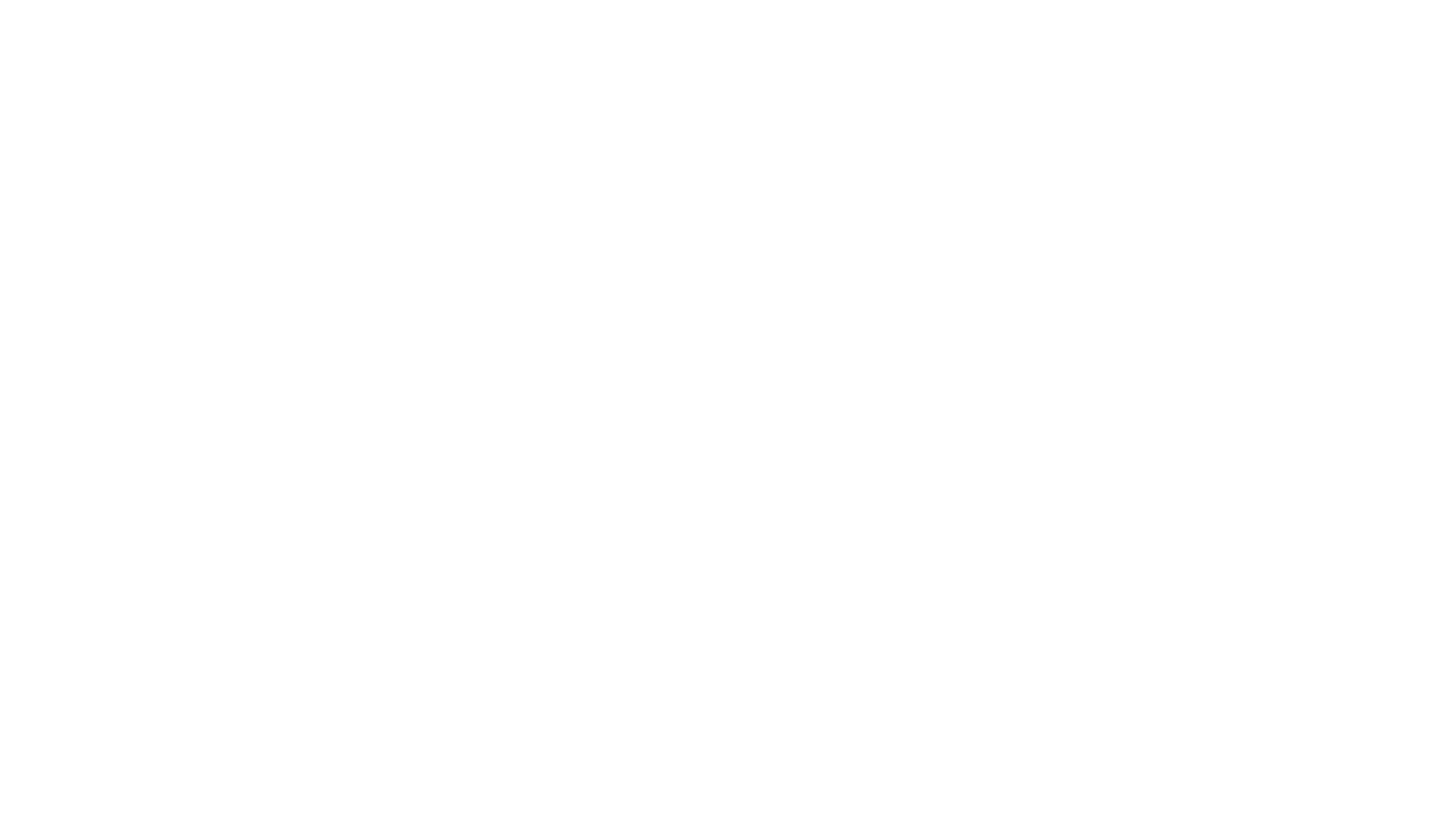

#

## Slide 61
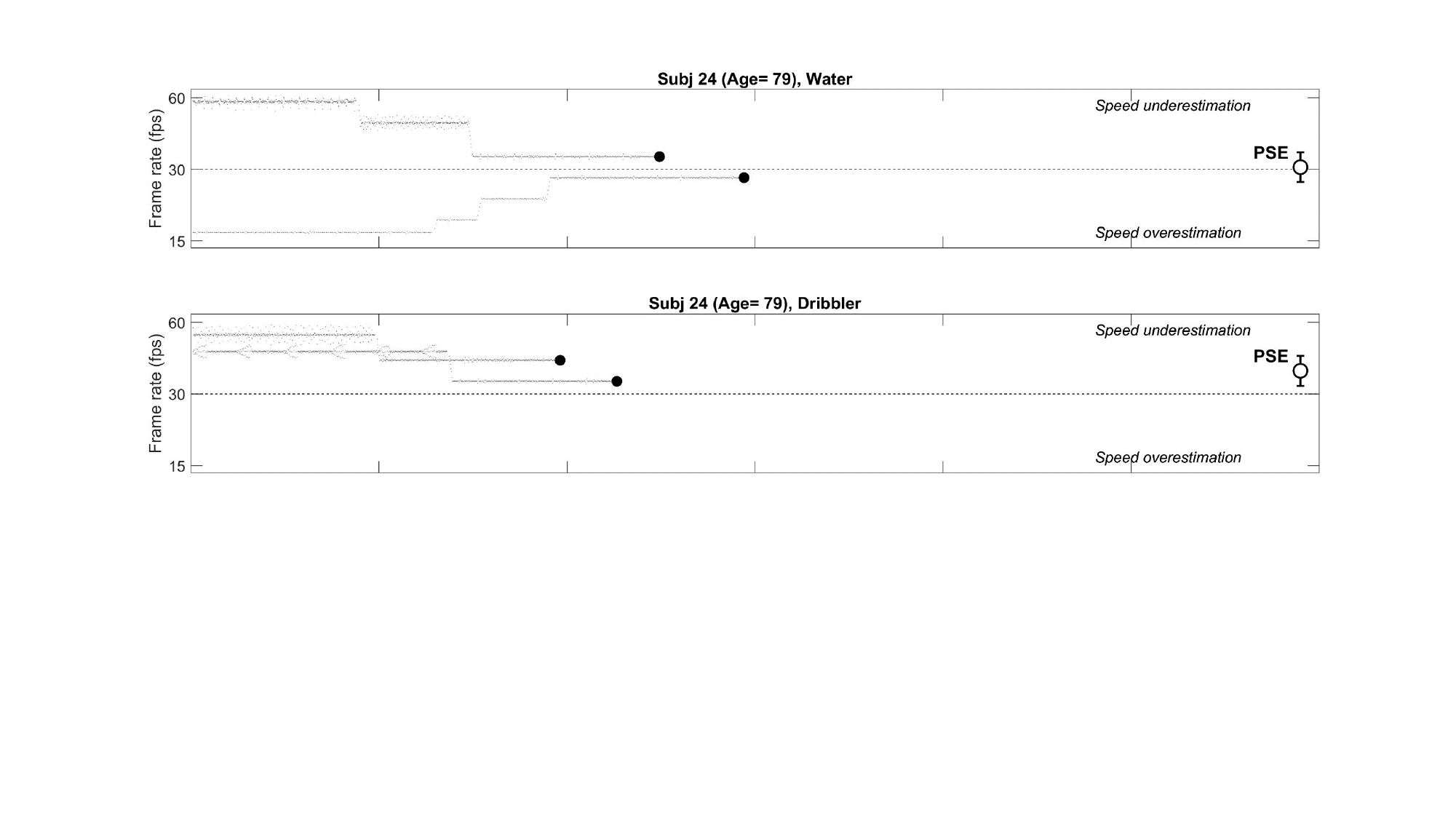

#

## Slide 62
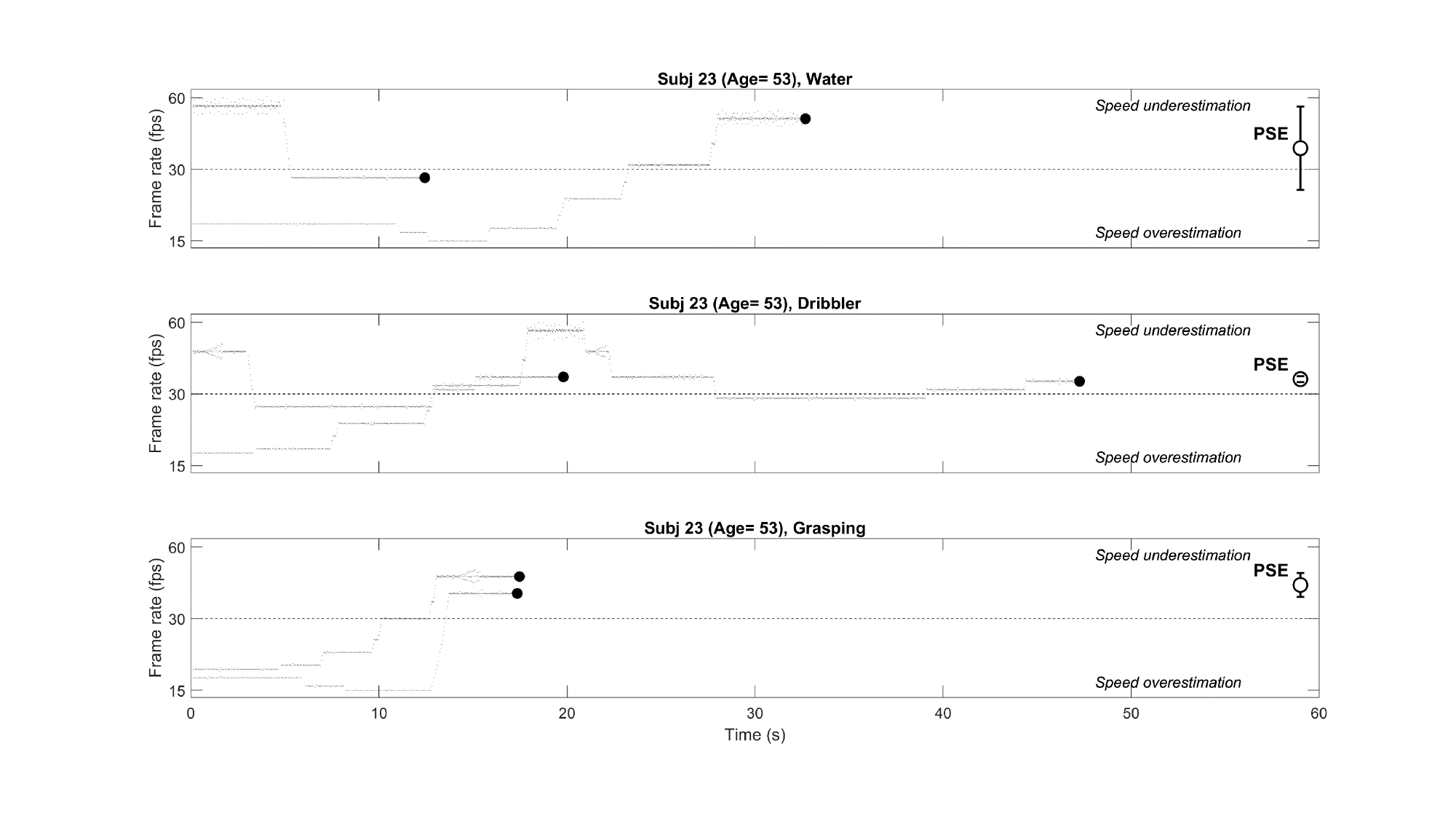

#

## Slide 63
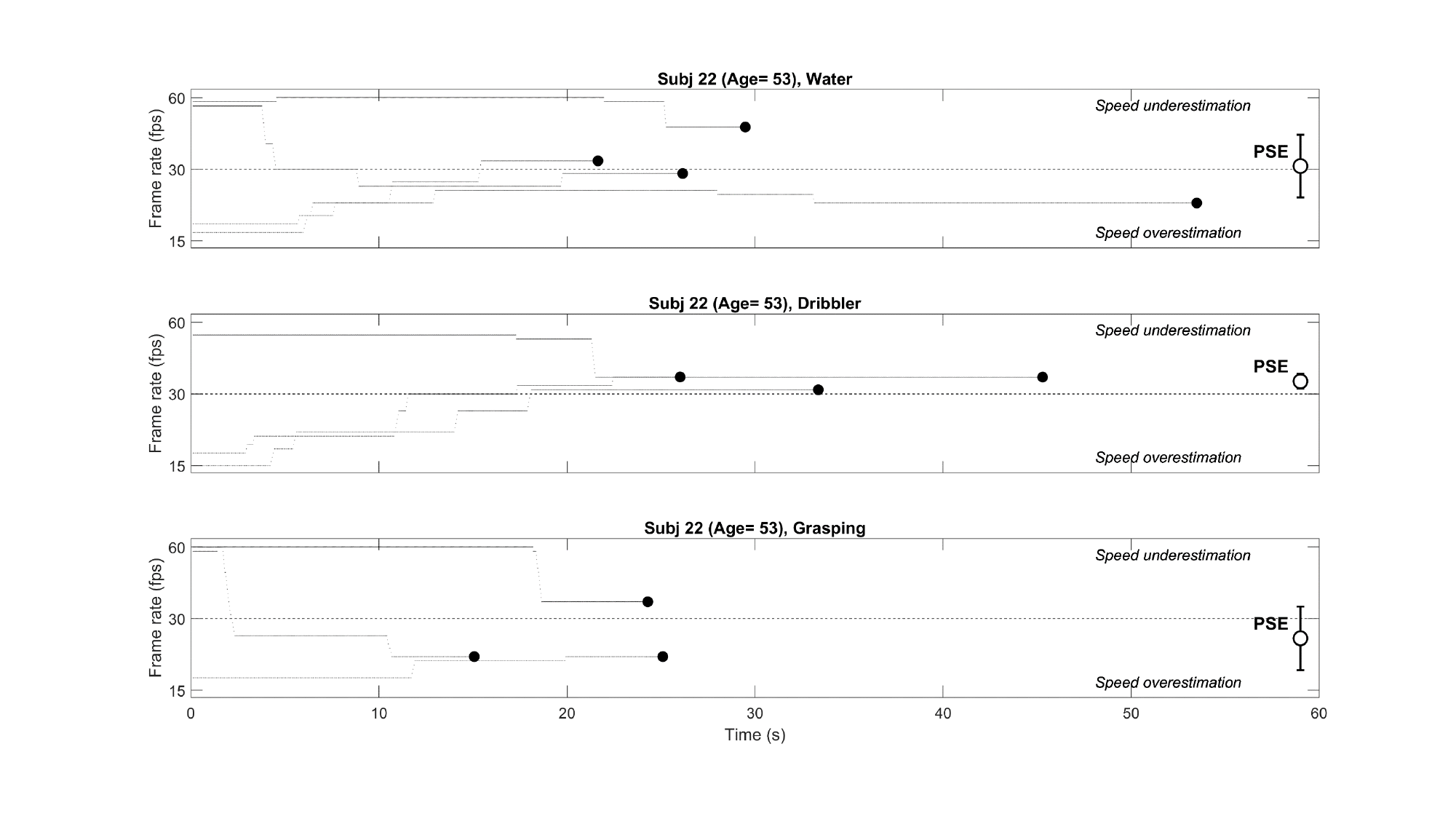

#

## Slide 64
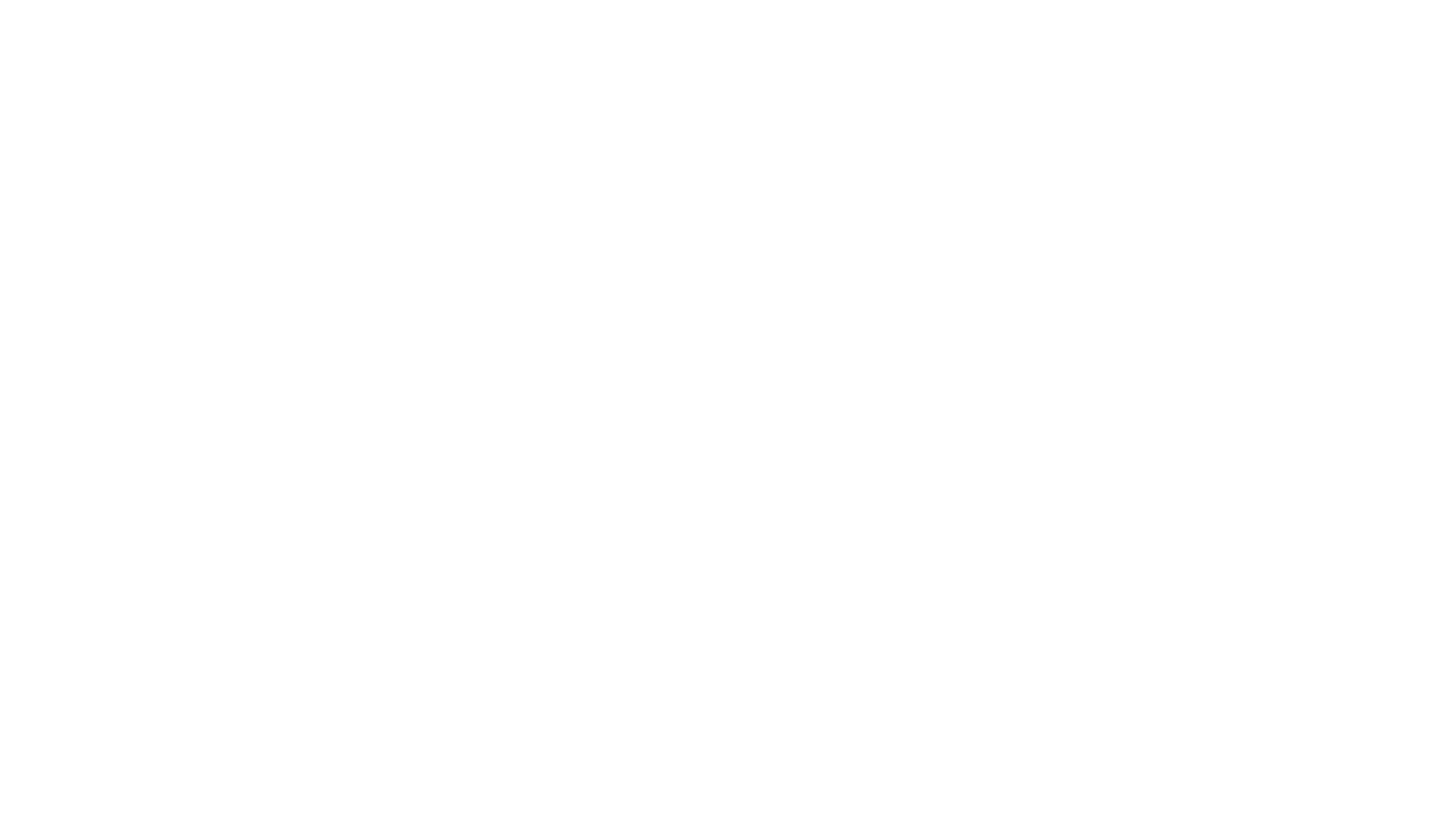

#

## Slide 65
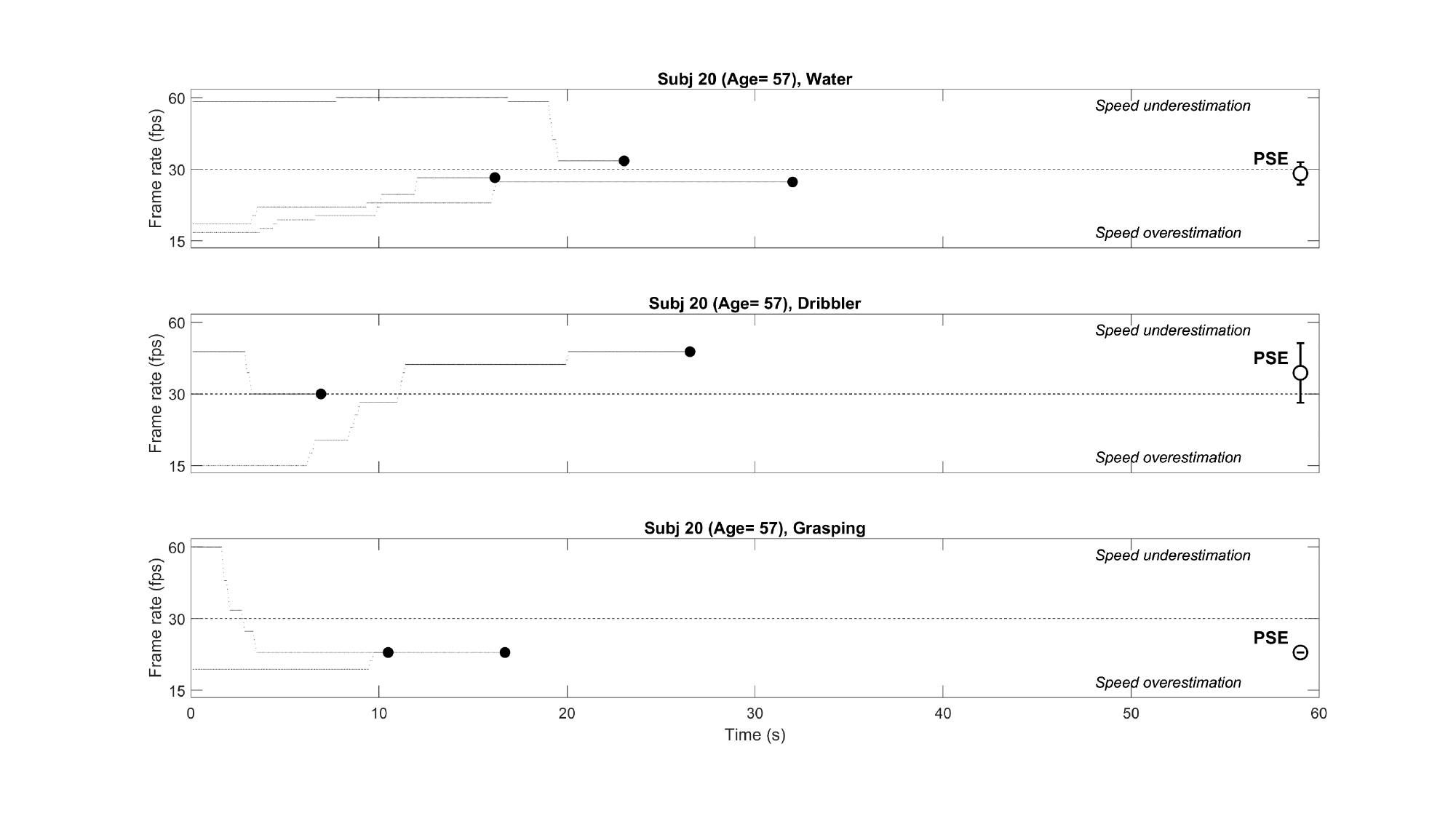

#

## Slide 66
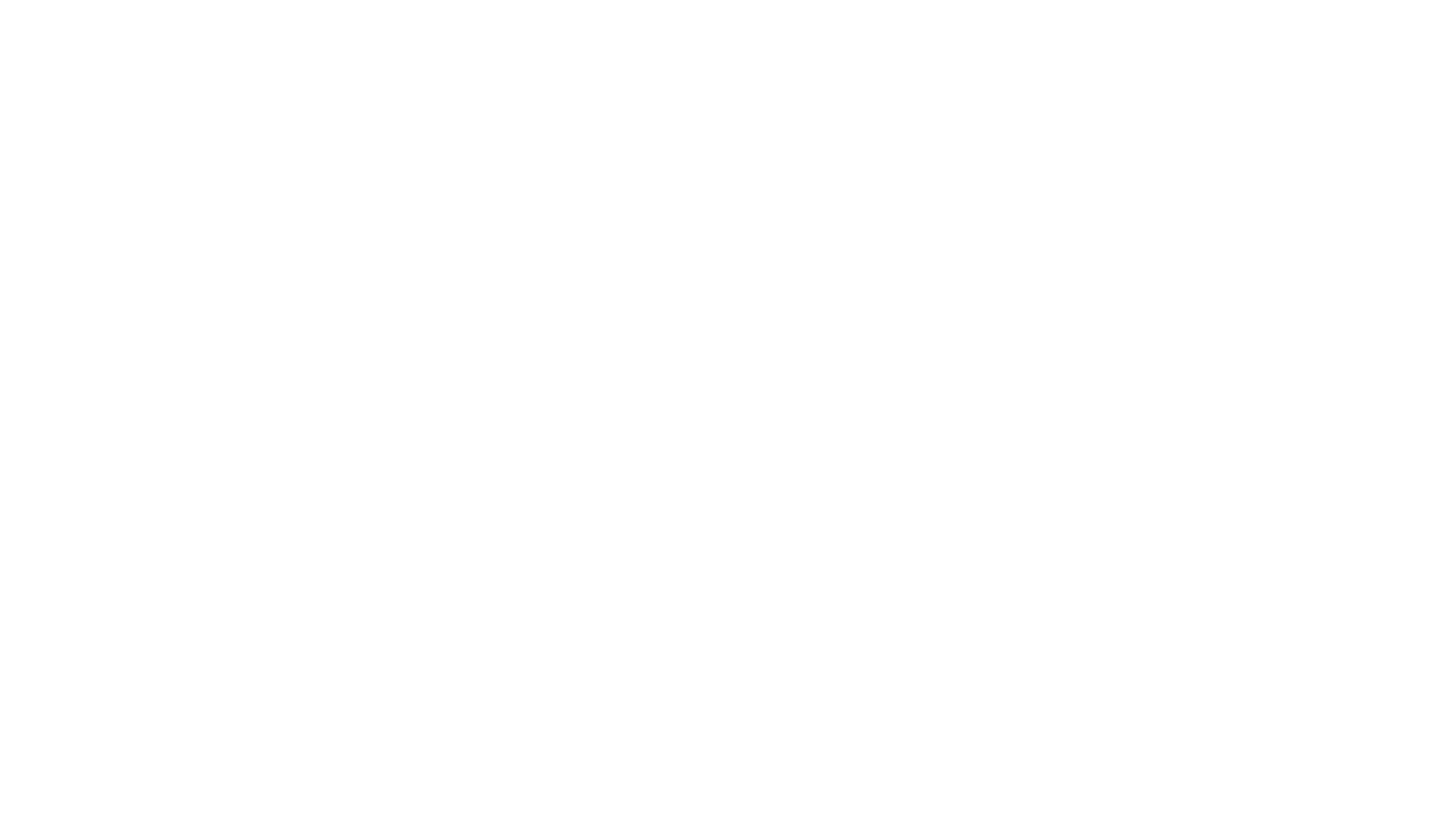

#

## Slide 67
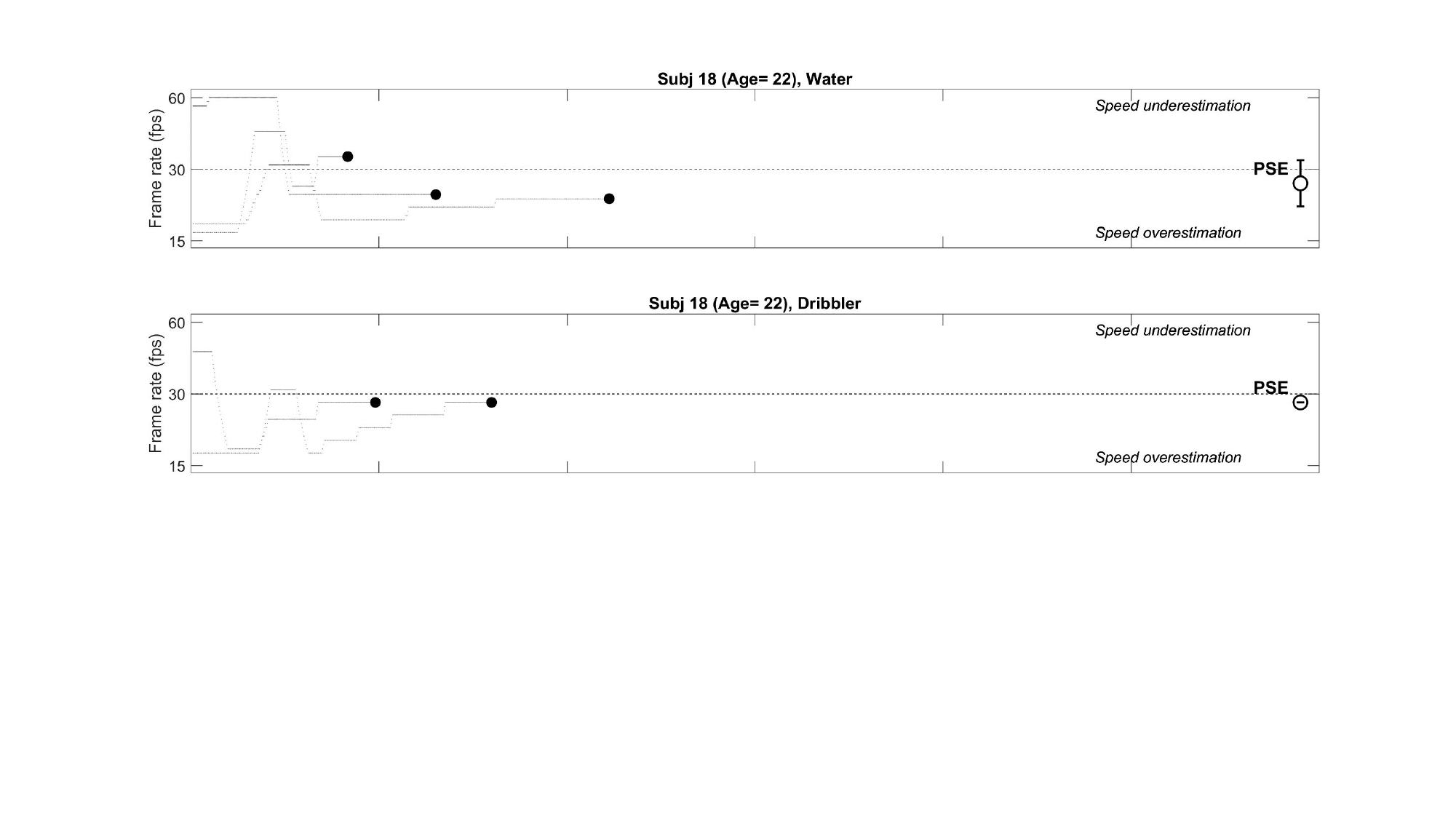

#

## Slide 68
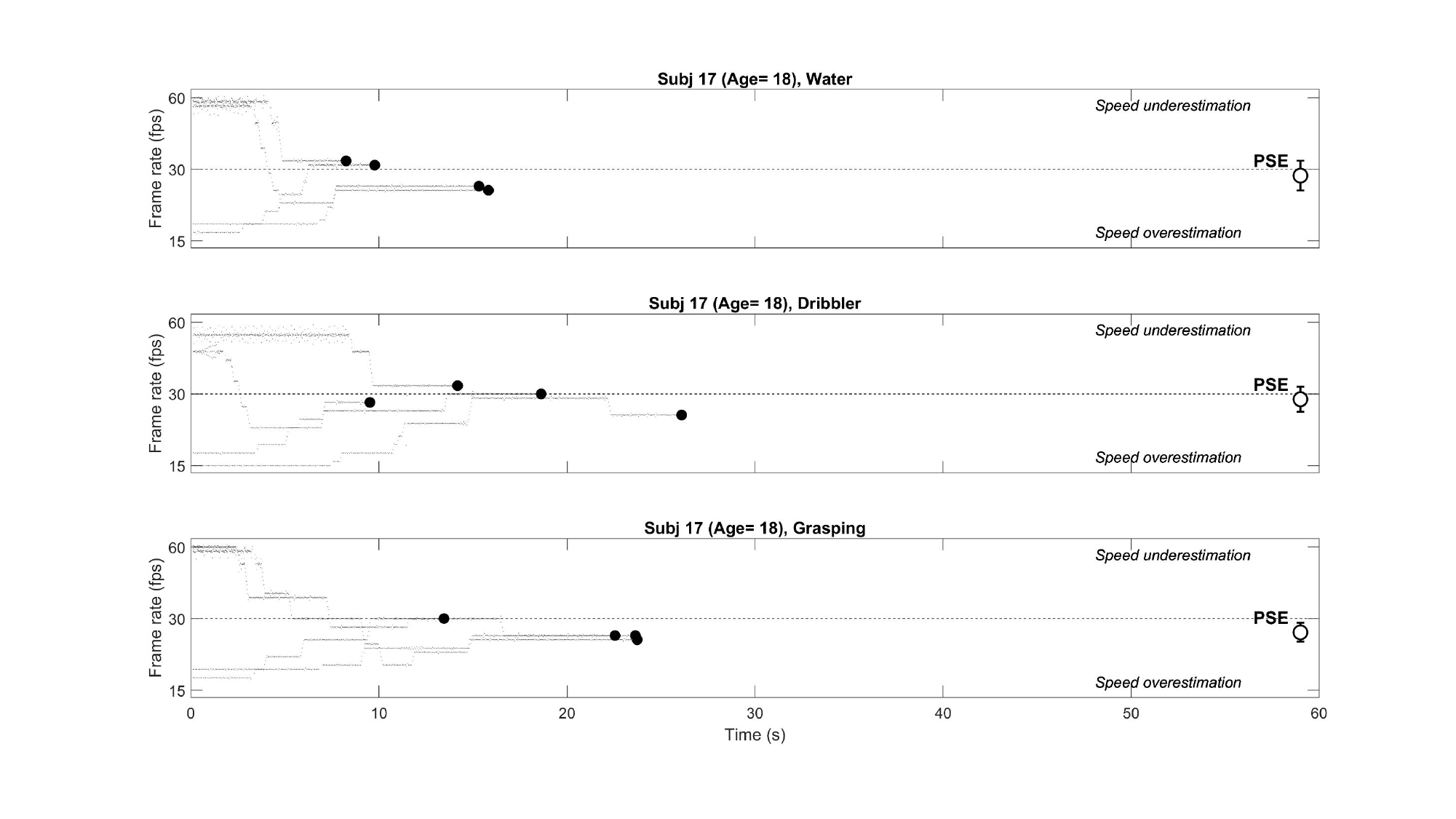

#

## Slide 69
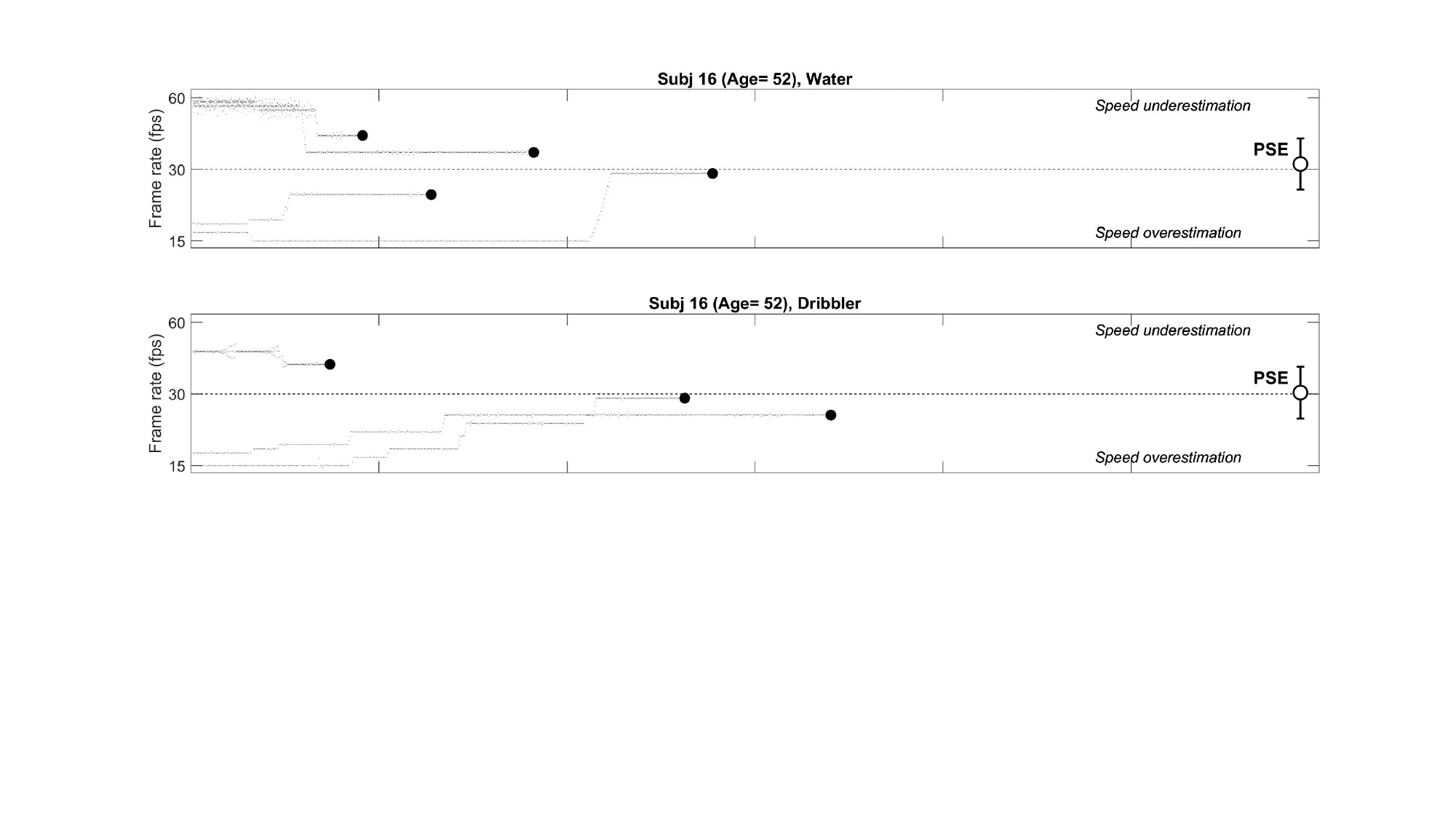

#

## Slide 70
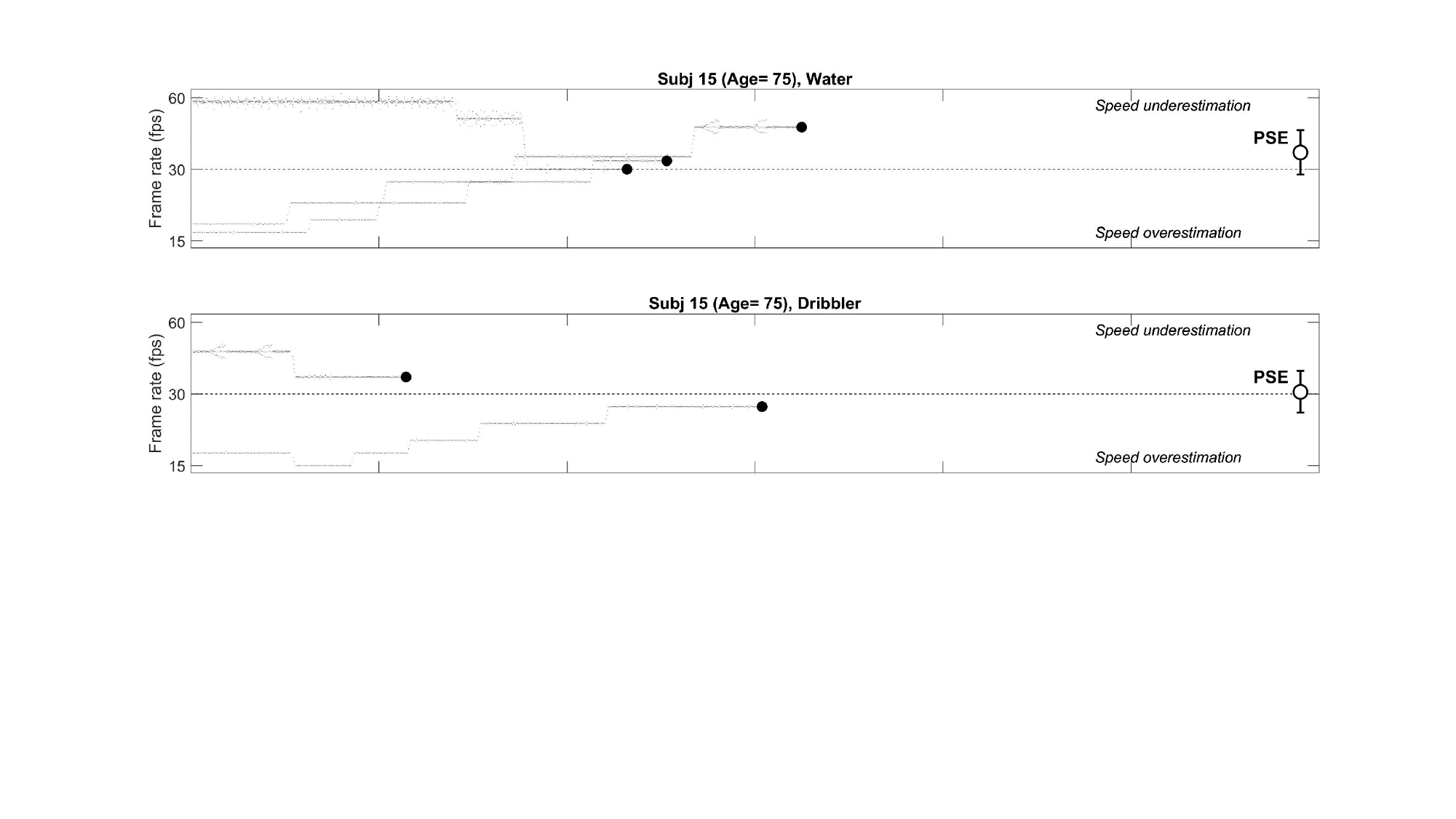

#

## Slide 71
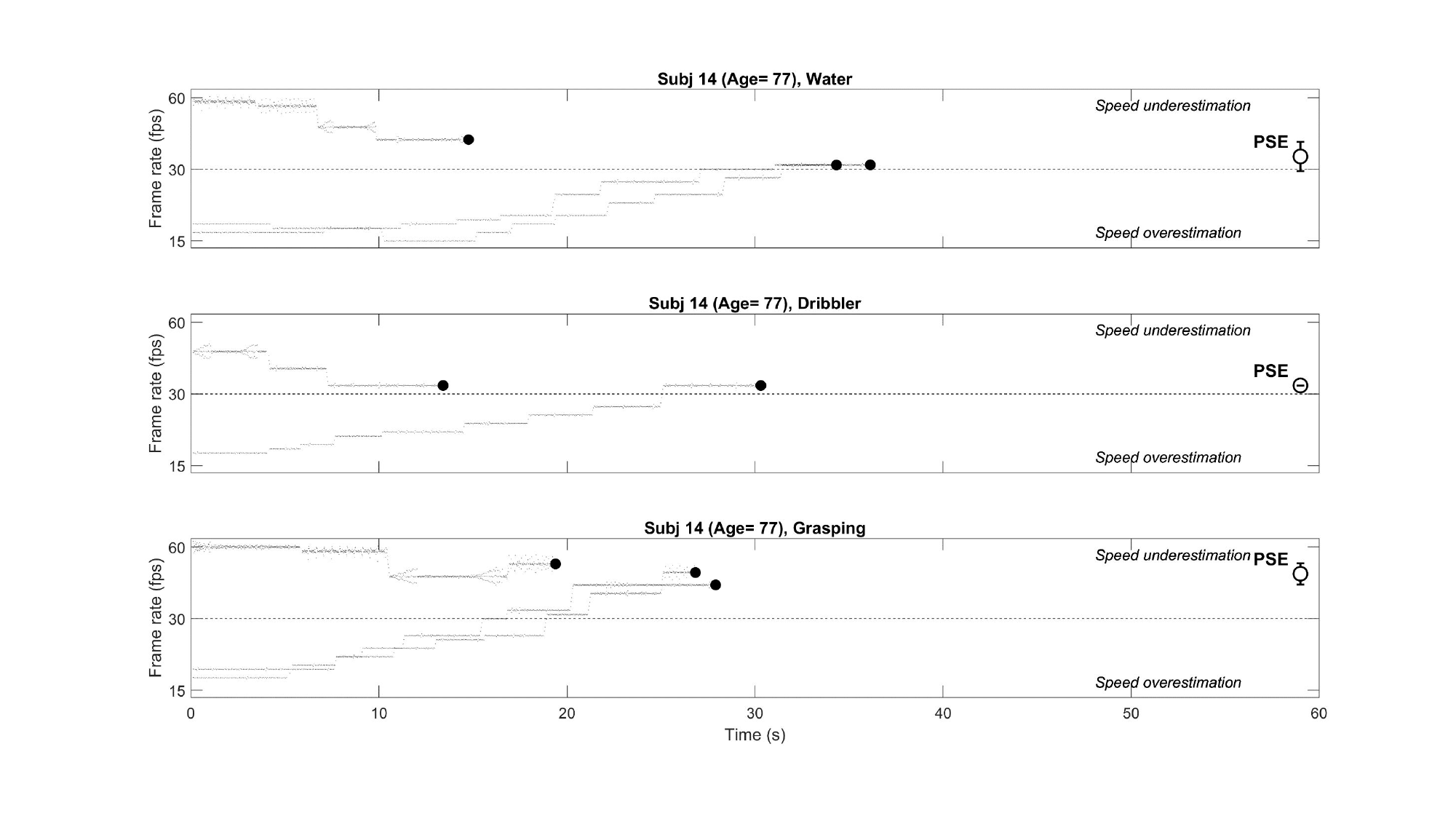

#

## Slide 72
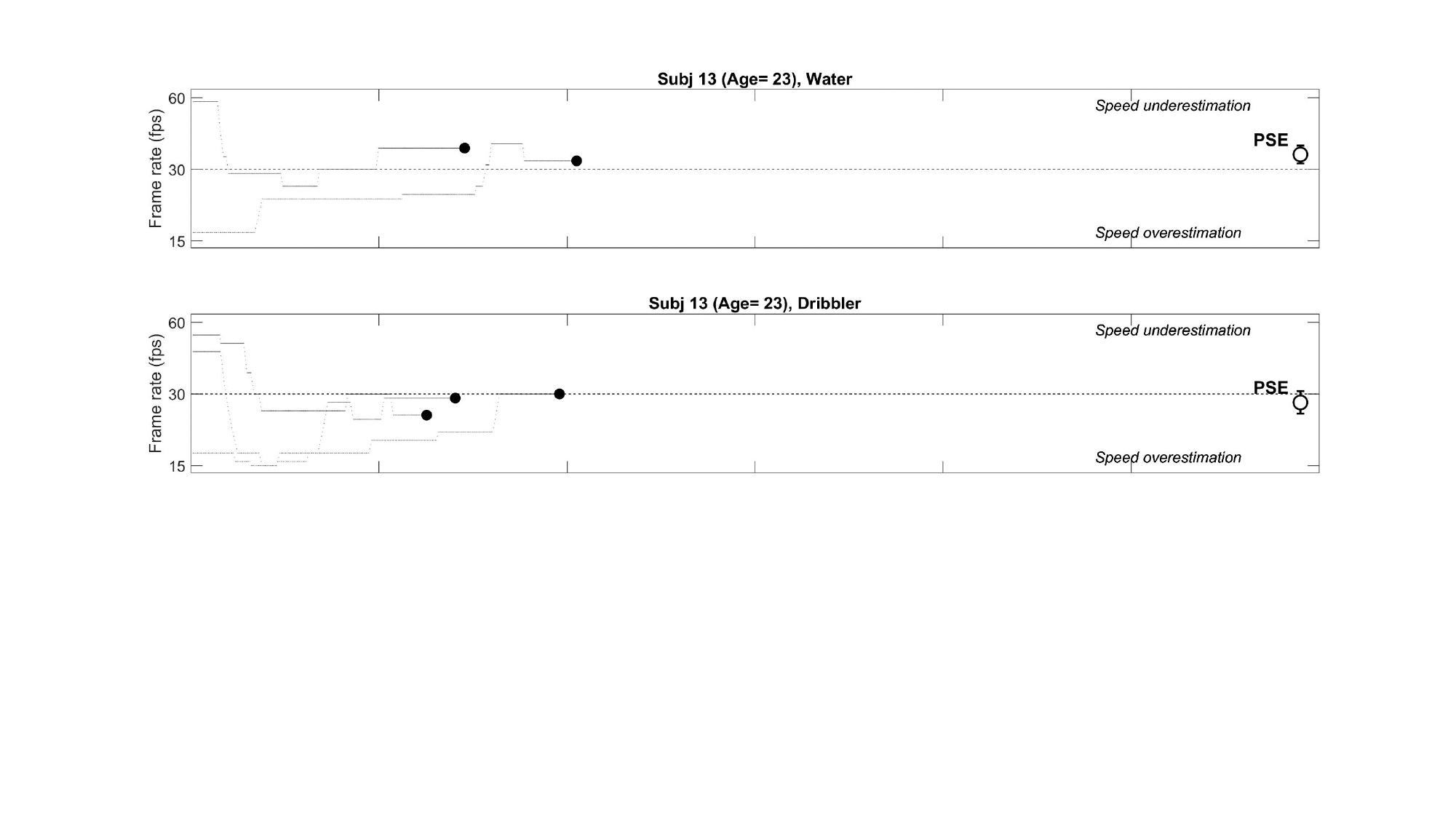

#

## Slide 73
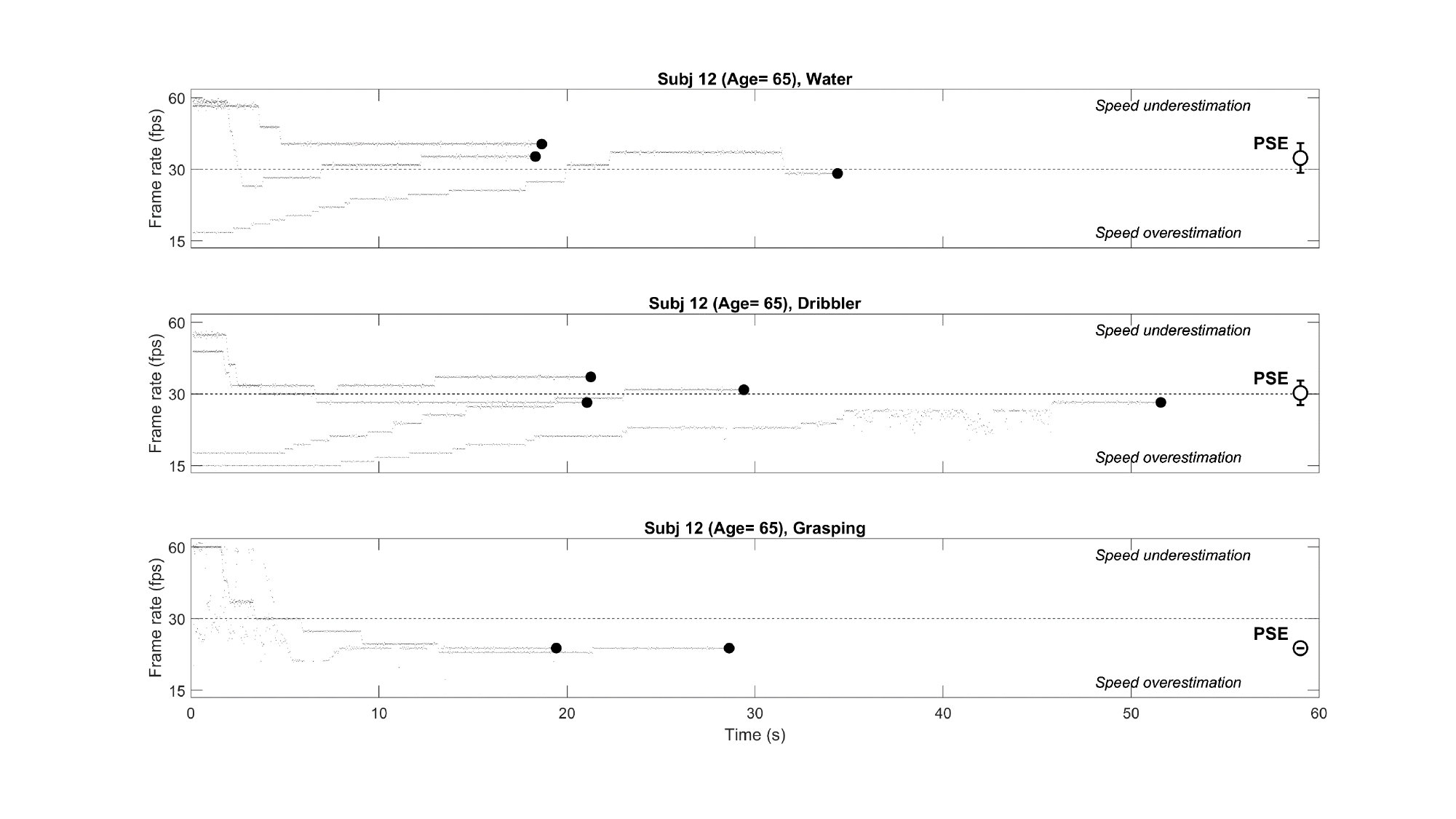

#

## Slide 74
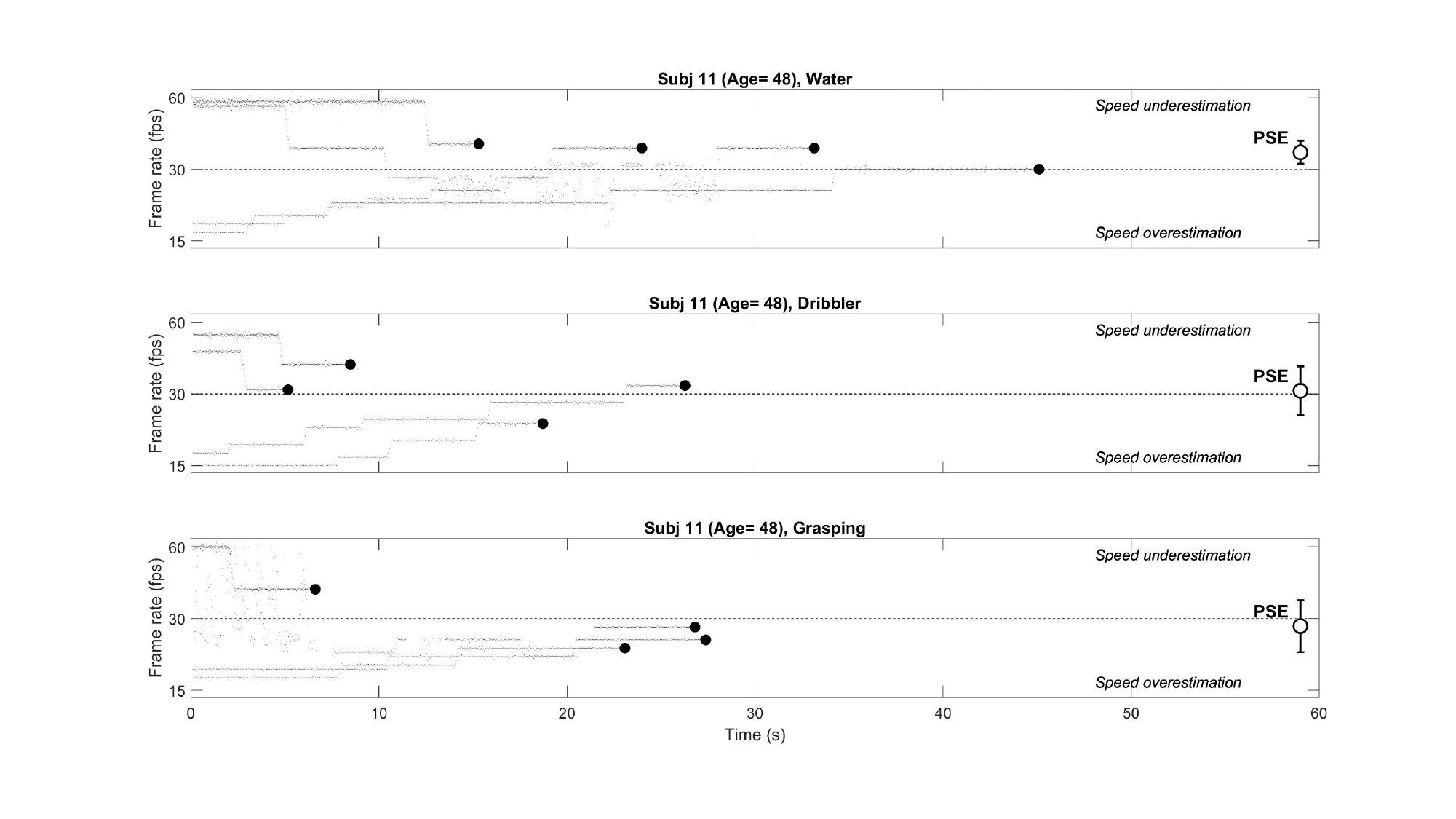

#

## Slide 75
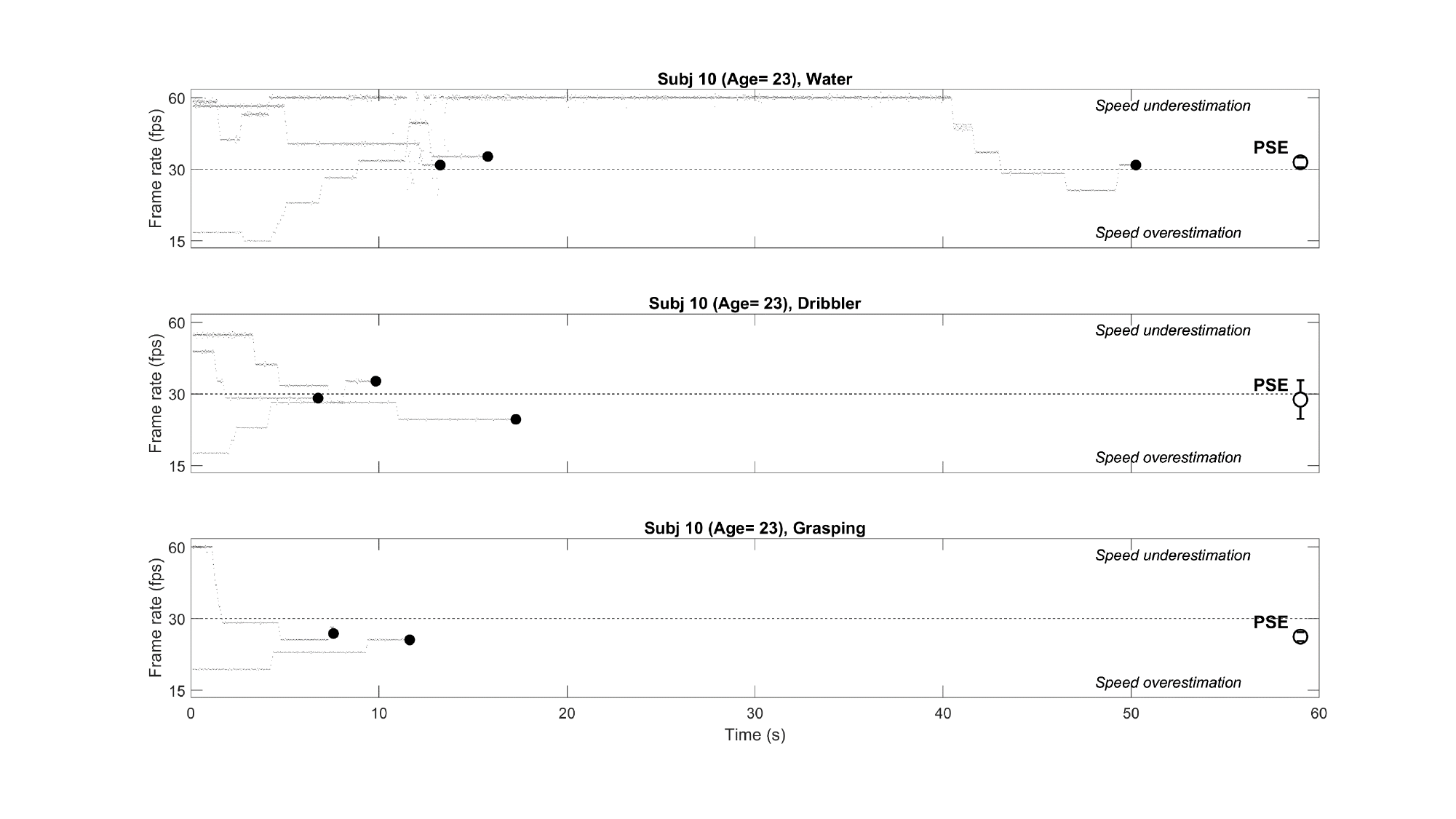

#

## Slide 76
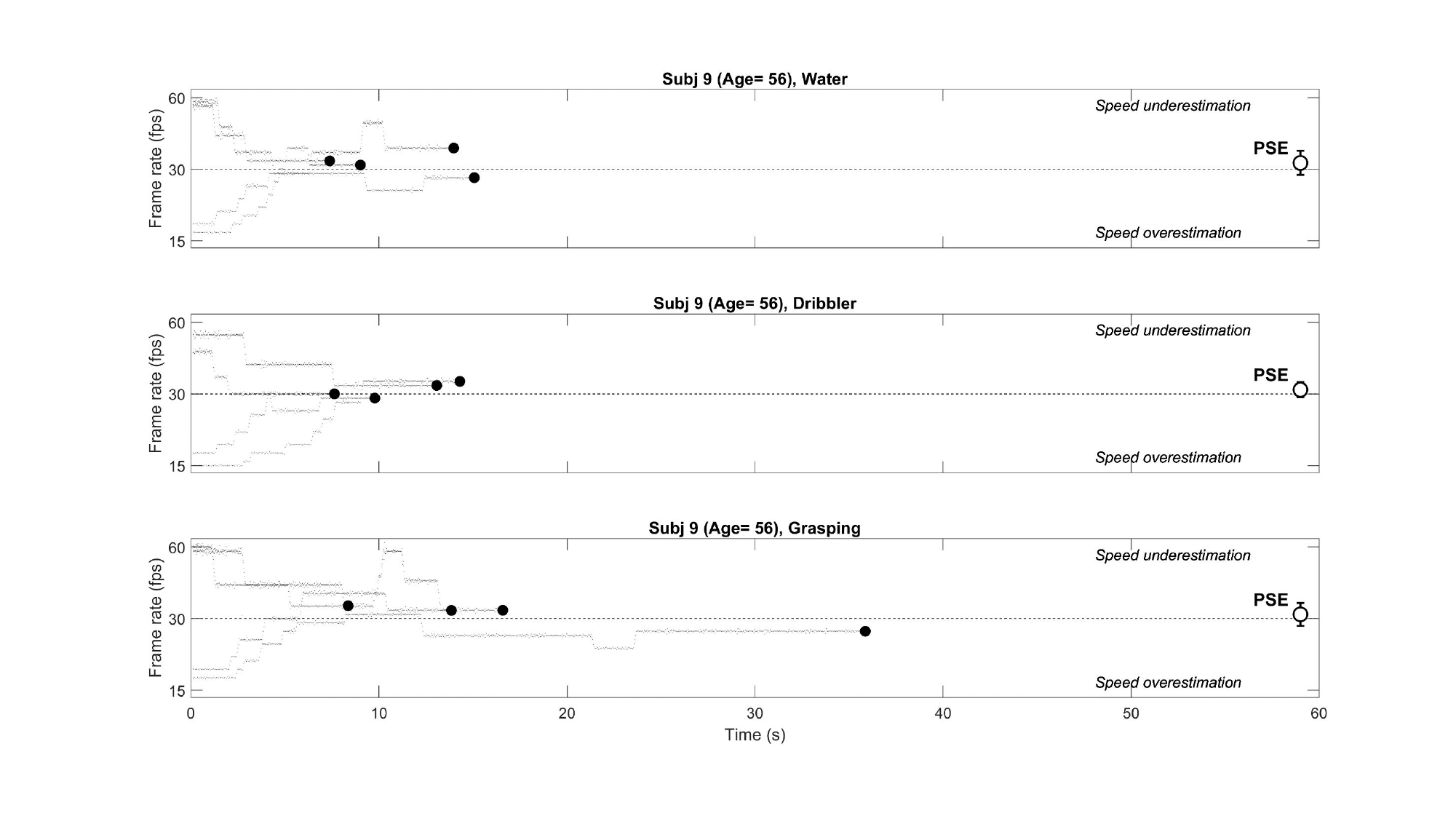

#

## Slide 77
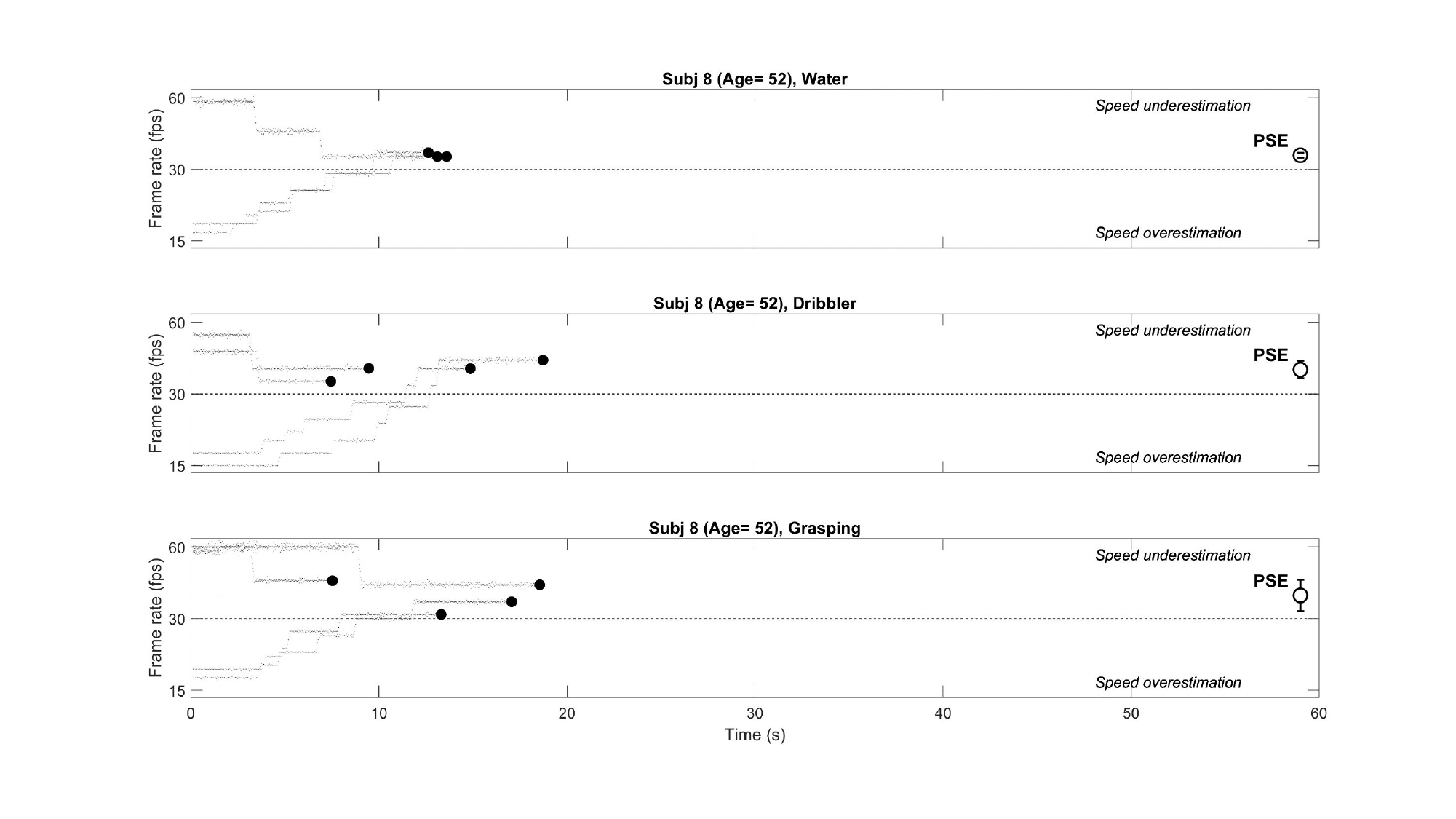

#

## Slide 78
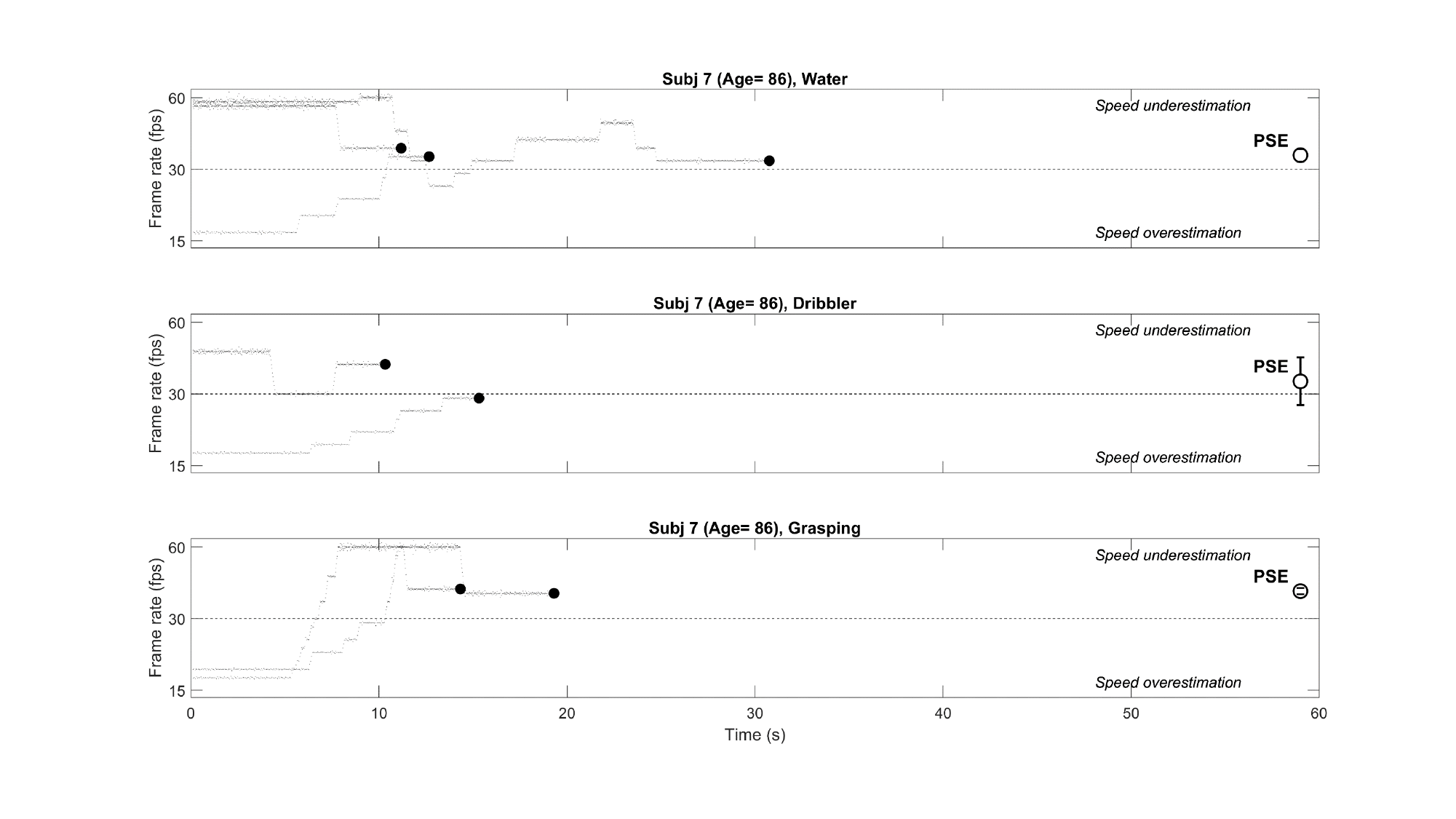

#

## Slide 79
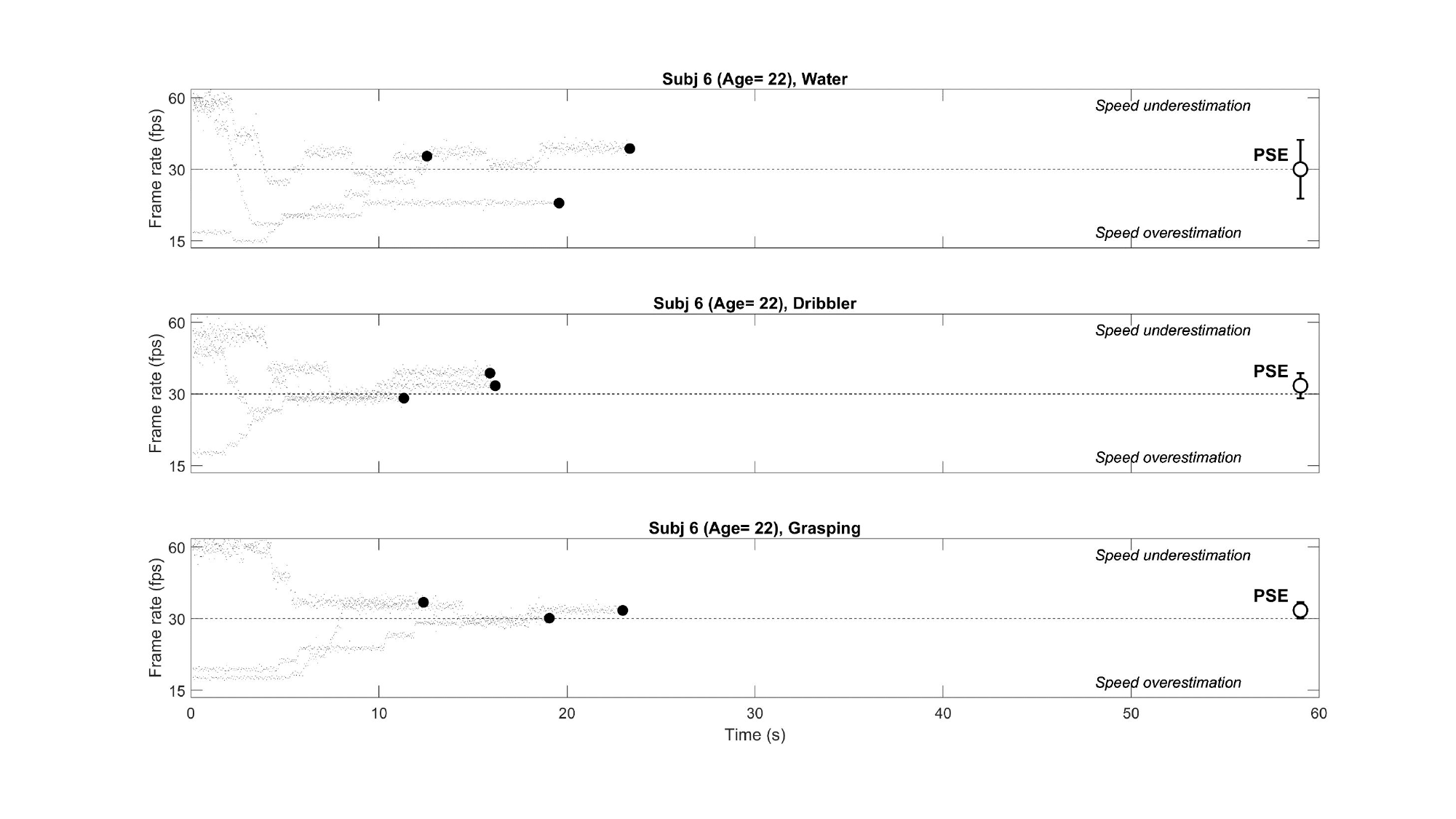

#

## Slide 80
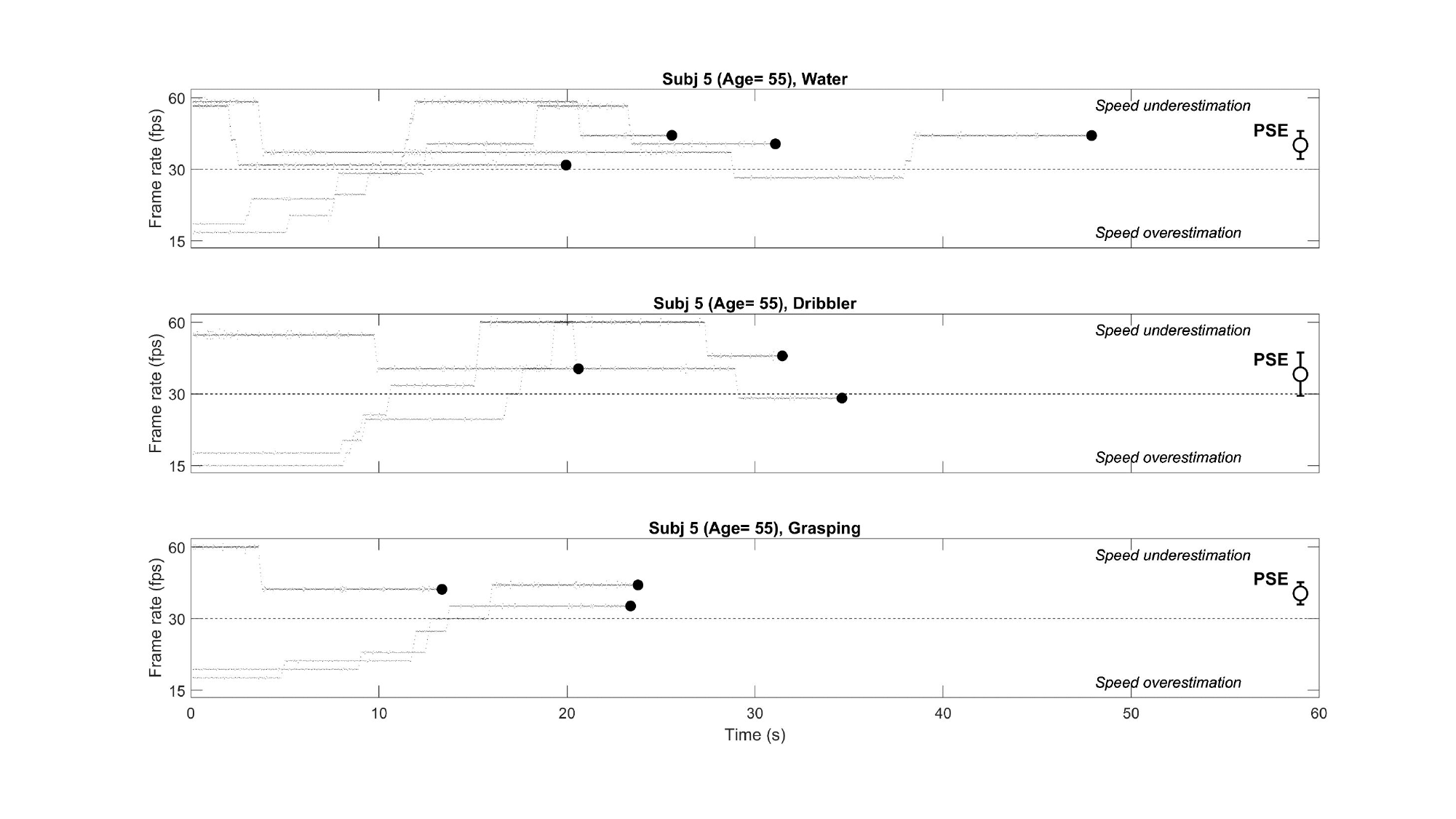

#

## Slide 81
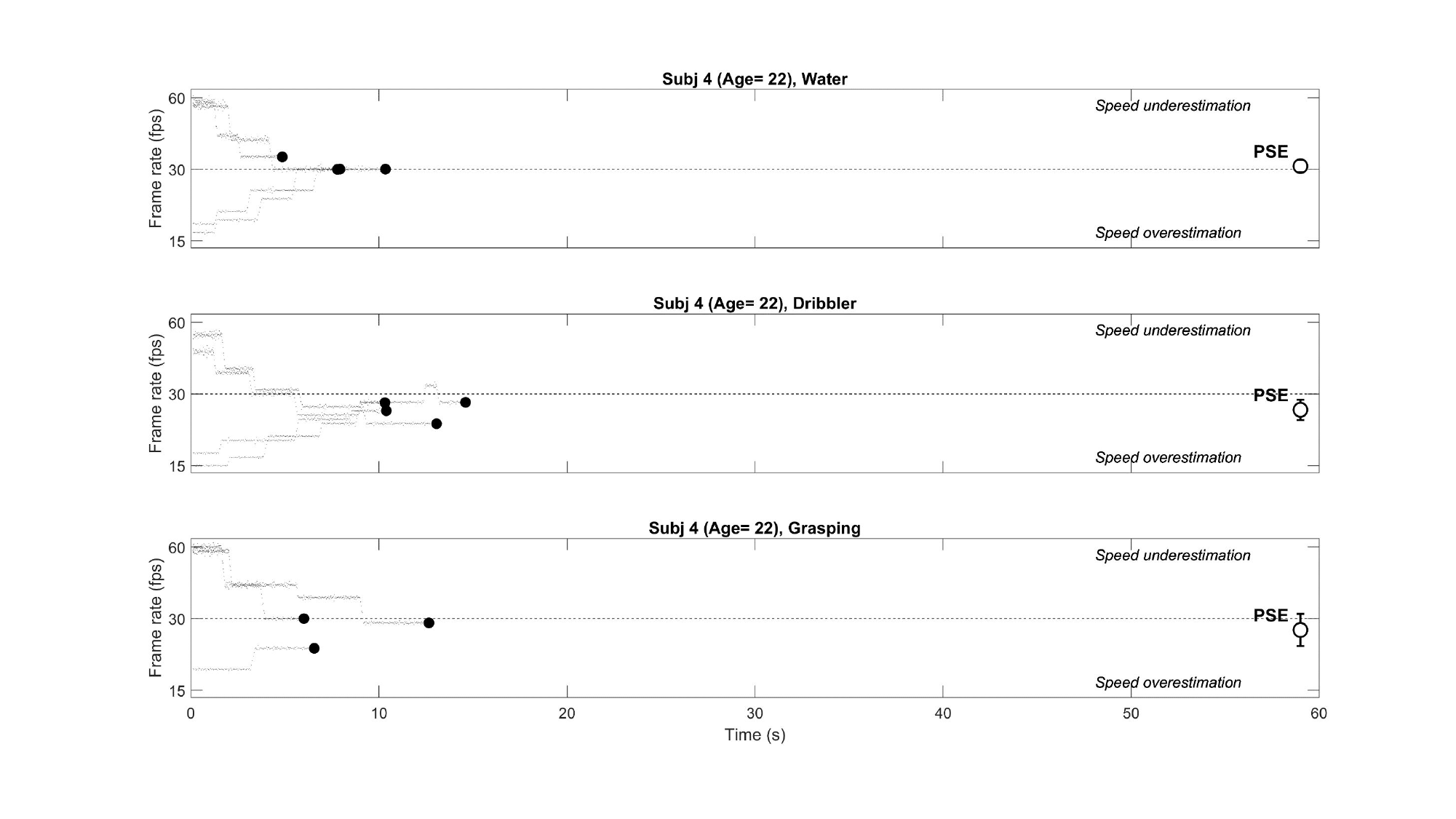

#

## Slide 82
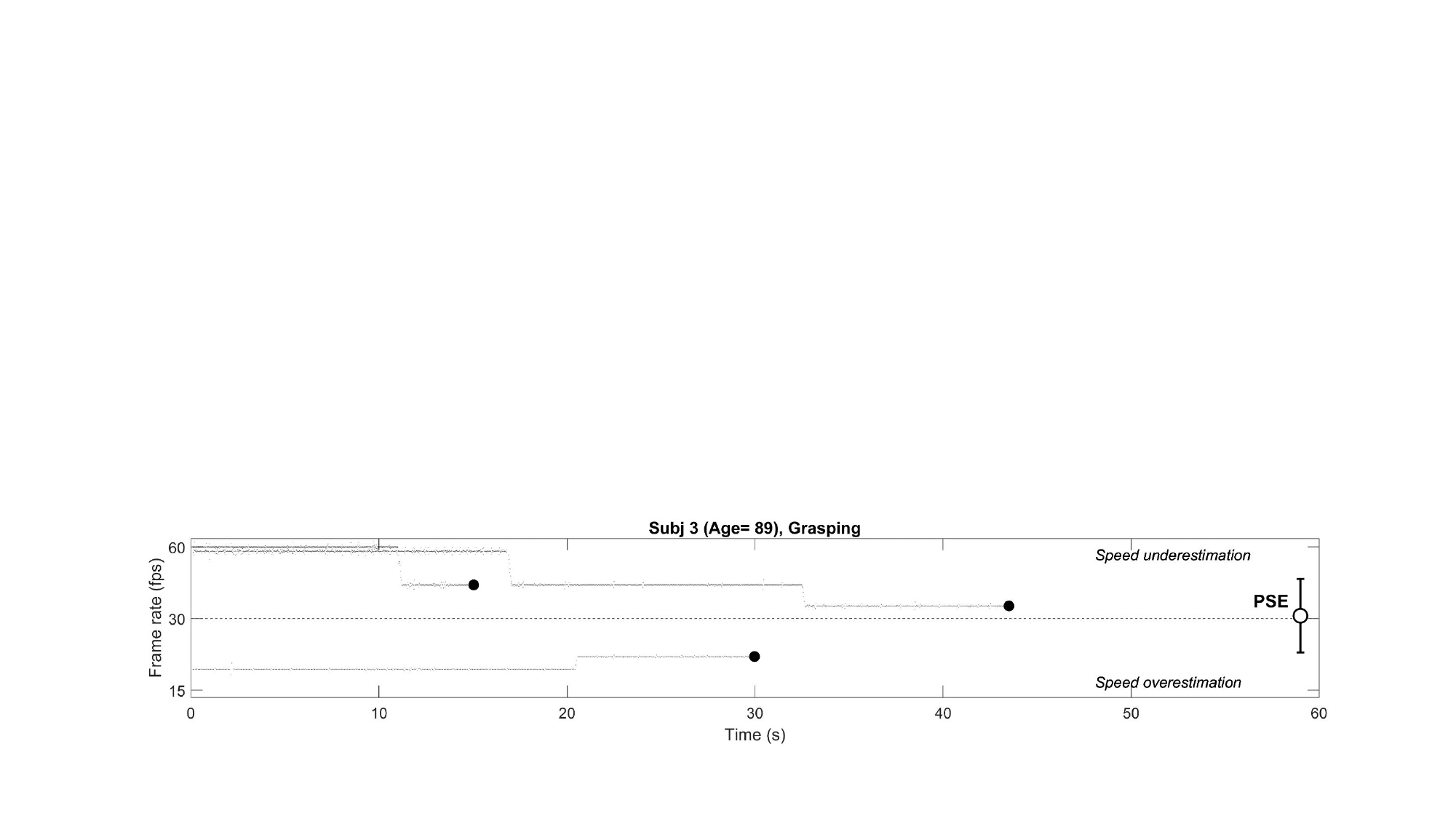

#

## Slide 83
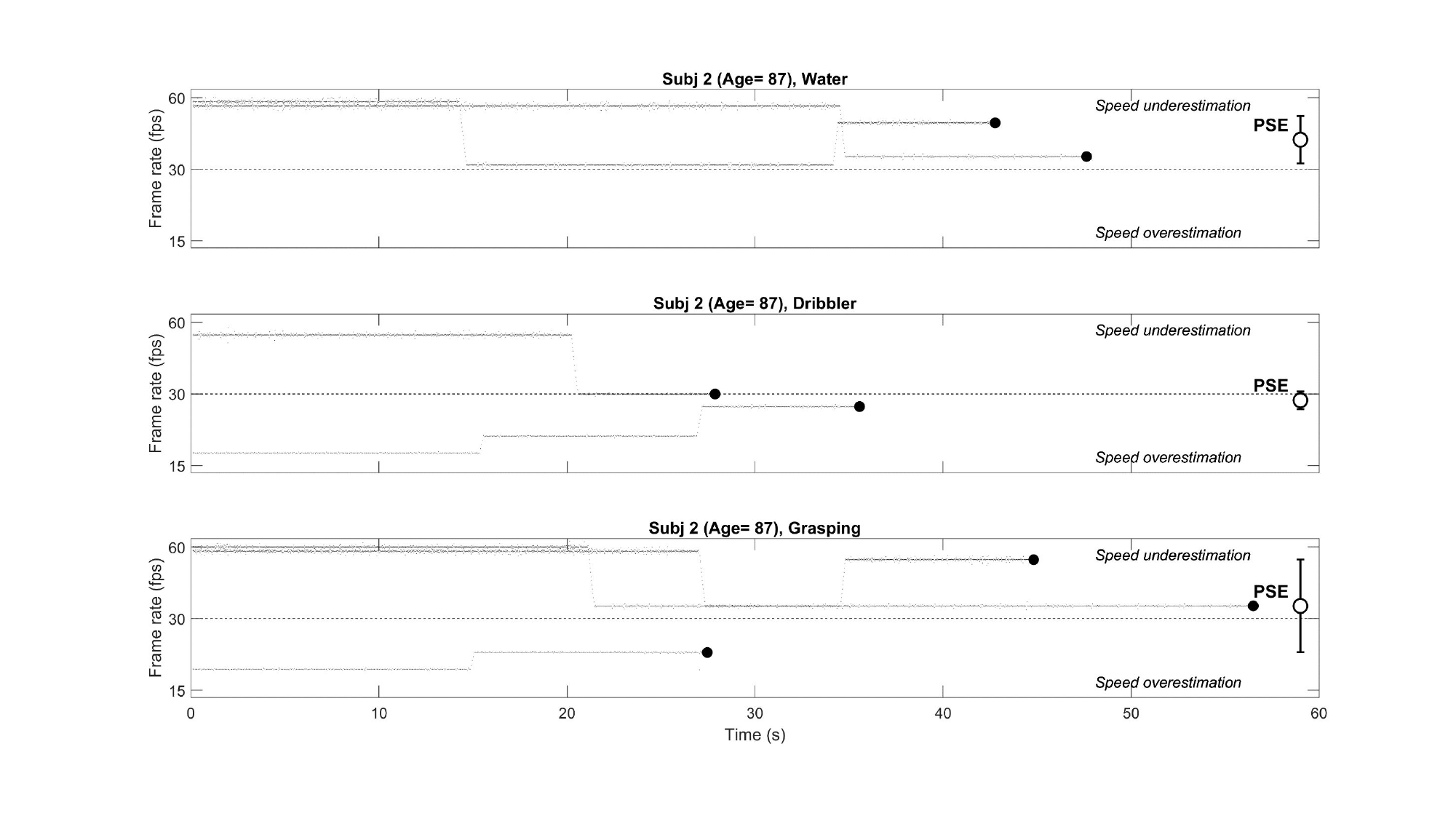

#

## Slide 84
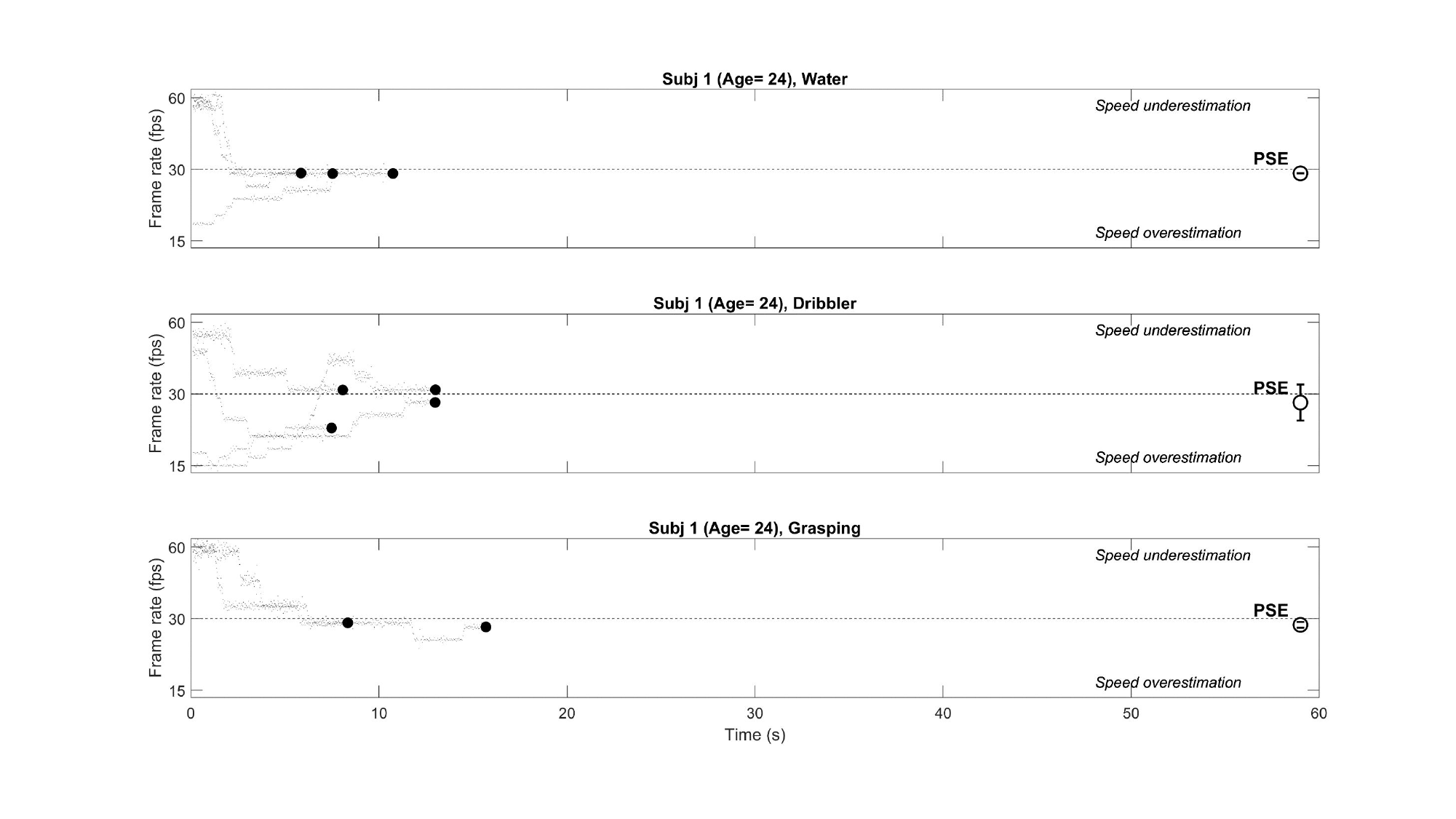

#
