# Supplementary material for: If You Are Old, Videos Look Slow. The Paradoxical Effect of Age-Related Motor Decline on the Kinematic Interpretation of Visual Scenes
Source: Front Hum Neurosci. 2022 Jan 5;15:783090. doi: 10.3389/fnhum.2021.783090 (PMC8766849; doi:10.3389/fnhum.2021.783090)
Supplement: Supplementary file 2 [file Data_Sheet_1.docx]

If you are old, videos look slow. The paradoxical effect of age-related motor decline on the kinematic interpretation of visual scenes.

Claudio de’Sperati, Marco Granato, Michela Moretti

Supplementary Material

**
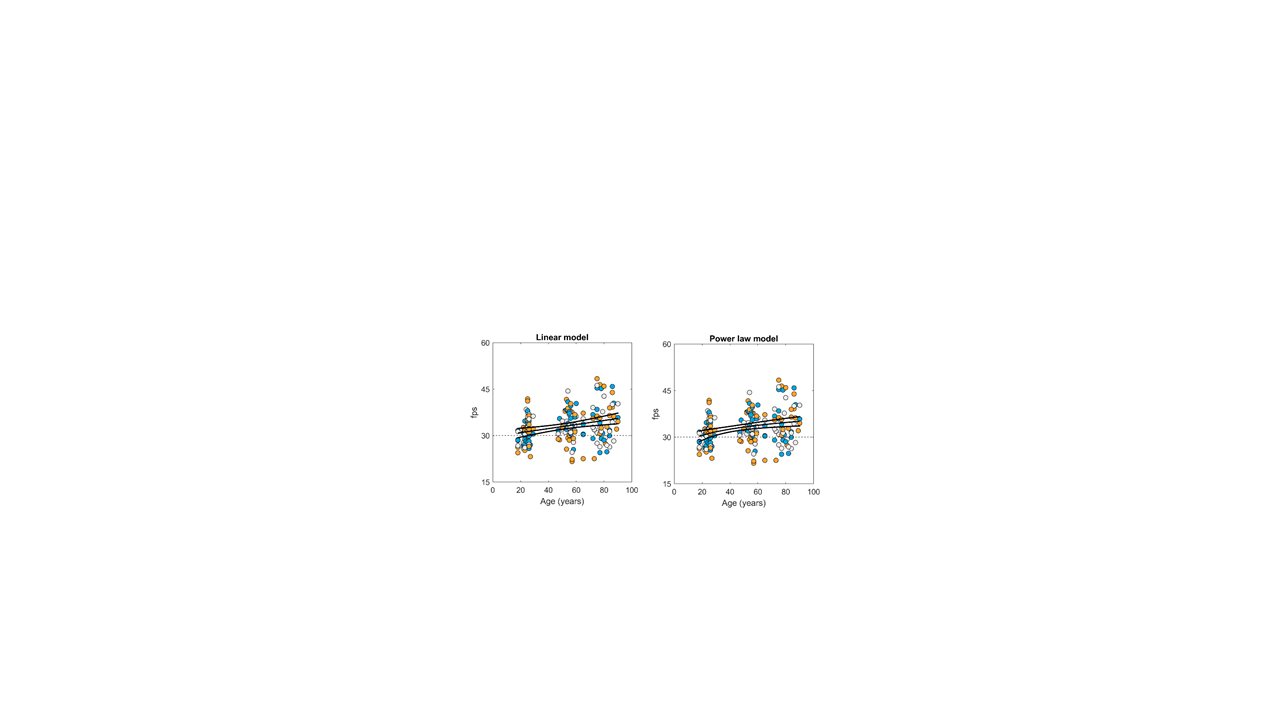
**

**Figure 1S.** Comparison of linear and non-linear (power law: y = a·x^b^) fitting models for the age-dependent PSE increase. Same conventions as in Fig. 2 of the main text.

| Predictor | Estimate | Standard error | T-value | P-value | Lower bound | Upper bound |
| --- | --- | --- | --- | --- | --- | --- |
| Age | -0.029 | 0.050 | -0.570 | 0.568 | -0.128 | 0.070 |
| Gender | 0.739 | 1.437 | 0.515 | 0.607 | -2.083 | 3.562 |
| MMscore | 0.667 | 0.702 | 0.950 | 0.342 | -0.712 | 2.045 |
| ADJ | -0.039 | 0.077 | -0.510 | 0.610 | -0.191 | 0.112 |
| CV | -0.033 | 0.089 | -0.378 | 0.706 | -0.208 | 0.141 |
| CT | -0.007 | 0.033 | -0.204 | 0.839 | -0.073 | 0.059 |
| VelMot | 31.599 | 11.167 | 2.830 | 0.005 | 9.661 | 53.537 |
| AccMot | 22.348 | 21.763 | 1.027 | 0.305 | -20.407 | 65.103 |
| VelAtt | 0.001 | 0.035 | 0.021 | 0.983 | -0.068 | 0.069 |
| AccAtt | -6.487 | 12.338 | -0.526 | 0.599 | -30.726 | 17.751 |
| InitSpeed | 0.057 | 0.012 | 4.625 | 0.000 | 0.033 | 0.081 |
| Selection | 0.204 | 1.453 | 0.140 | 0.888 | -2.650 | 3.058 |

**Table 1S.**Results of the trial-wise LMM analysis using data from the speeded tapping task. The lower and upper bounds indicate the 95% confidence intervals of the coefficients’ estimates. Dependent variable = final video speed. MMscore = MMSE score, ADJ = adjustments, CV = coefficient of variation, CT = completion time, VelMot = motor speed, AccMot = motor accuracy, VelAtt = attentional speed, AccAtt = attentional accuracy, InitSpeed = initial video speed, Selection = trial selection criteria (strict or loose).

| Predictor | Estimate | Standard error | T-value | P-value | Lower bound | Upper bound |
| --- | --- | --- | --- | --- | --- | --- |
| Age | 0.070 | 0.044 | 1.572 | 0.116 | -0.017 | 0.1575 |
| Gender | 0.274 | 1.498 | 0.183 | 0.855 | -2.670 | 3.217 |
| MMSE | 0.330 | 0.685 | 0.481 | 0.630 | -1.016 | 1.676 |
| ADJ | -0.089 | 0.073 | -1.208 | 0.227 | -0.233 | 0.055 |
| CV | -0.027 | 0.092 | -0.291 | 0.771 | -0.208 | 0.154 |
| CT | -0.001 | 0.033 | -0.031 | 0.975 | -0.067 | 0.065 |
| VelMot | 1.763 | 3.557 | 0.496 | 0.620 | -5.224 | 8.751 |
| AccMot | 0.571 | 7.810 | 0.073 | 0.942 | -14.770 | 15.912 |
| VelAtt | 0.007 | 0.037 | 0.209 | 0.834 | -0.065 | 0.080 |
| AccAtt | -6.176 | 13.299 | -0.464 | 0.642 | -32.301 | 19.948 |
| InitSpeed | 0.054 | 0.012 | 4.563 | 0.000 | 0.0310 | 0.078 |
| Selection | 0.094 | 1.473 | 0.064 | 0.949 | -2.801 | 2.989 |

**Table 2S.**Results of the trial-wise LMM analysis using data from the relaxed tapping task. Same conventions as in Table 1S.
